# Effectiveness of digital home rehabilitation and supervision for stroke survivors: A systematic review and meta-analysis

**DOI:** 10.1177/20552076241256861

**Published:** 2024-06-03

**Authors:** Ann Marie Hestetun-Mandrup, Zheng An Toh, Hui Xian Oh, Hong-Gu He, Anne Catrine Trægde Martinsen, Minna Pikkarainen

**Affiliations:** 1 Oslomet -Oslo Metropolitan University, Oslo, Norway; 2 87550 Sunnaas Rehabilitation Hospital, Norway; 3 Alice Lee Centre for Nursing Studies, 63751 Yong Loo Lin School of Medicine, National University of Singapore; 4 37581 Singapore General Hospital, Singapore; 5 6370 University of Oulu, Oulu, Finland; 6 National University Health System, Singapore

**Keywords:** Digital, home, meta-analysis, motor ability, post-stroke, quality of life, telerehabilitation

## Abstract

**Objective:**

Stroke survivors often experience residual impairments and motor decline post-discharge. While digital home rehabilitation combined with supervision could be a promising approach for reducing human resources, increasing motor ability, and supporting rehabilitation persistence there is a lack of reviews synthesizing the effects. Thus, this systematic review and meta-analysis aimed to synthesize the effect of digital home rehabilitation and supervision in improving motor ability of upper limb, static balance, stroke-related quality of life, and self-reported arm function among stroke survivors.

**Methods:**

Six electronic databases, grey literature, ongoing studies, and reference lists were searched for relevant studies. Two investigators independently reviewed titles, abstracts, screened full texts for eligibility and performed data extraction. Meta-analysis of 13 independent studies were grouped into four separate meta-analyses. The Grading of Recommendations, Assessments, Development and Evaluations (GRADE) tool was used for evaluating the overall quality of the evidence.

**Results:**

Meta-analyses showed no statistically significant difference between intervention (digital home rehabilitation) and control groups (home training/clinic-based) of all outcomes including motor ability of upper limb, static balance, stroke-related quality of life, and self-reported arm function. In the sub-group analysis digital home rehabilitation was associated with better quality of arm use (standardized mean difference = 0.68, 95% confidence interval: [0.27, 1.09], *p *= 0.001).

**Conclusions:**

This result indicated that digital home rehabilitation has similar effects and could potentially replace home training or clinic-based services. This review highlights better-targeted digital motor interventions to examine the effects of interventions further. The quality of evidence was moderate to high in motor and self-reported arm outcomes, and low for balance and quality of life.

## Introduction

Stroke, a cerebral infarction, or haemorrhage in the brain, causes physical, cognitive, and somatosensory long-term disabilities for stroke survivors.^1,2^ Globally, it is estimated to affect 11 million people per year worldwide.^3^ Many stroke survivors have residual functional impairments, where motor sequala is reported as the most common disability,^2^ especially decreased unilateral hand function and strength.^4^ An European longitudinal multicentre study showed that 63% of chronic stroke survivors lacked physical therapy and found a significant decline in four motor outcomes between six months and 5 years post-stroke.^5^ While the recovery tends to be more pronounced during the acute and subacute stages of stroke, with remaining disabilities often becoming more permanent, rehabilitation programmes continue to demonstrate effectiveness during the chronic phase.^6^ Previous studies indicated that telerehabilitation technologies in stroke care may serve as an efficient approach to meet these challenges.^7–11^

Remote stroke rehabilitation, or telerehabilitation, enables a possibility for continuous rehabilitation and supervision^7,9,11,12^ between stroke survivors and health care professionals in many fields of care.^13^ Telerehabilitation, when compared to traditional training at health institutions, has been recognized as beneficial due to its convenience and ability to overcome socioeconomic, geographical, and cultural challenges. Additionally, it may help reduce disparities and promote equity. However, further work on infrastructure is necessary to ensure widespread accessibility and opportunity.^14^

Current telerehabilitation technologies have advanced over the last years and span over synchronous solutions (e.g., videoconferencing) or asynchronous (messaging or data collection) towards remote monitoring and programmes on different digital applications.^13,15^ Further, the past Covid-19 pandemic has led to out-sourced digital rehabilitation where an expansion of new technologies have arisen,^15^ characterized as decision support systems, motion detection and sensor-guided home exercises evolved by more volume, velocity, and variety and predictive artificial intelligence.^16–18^ However, the utilization of digital home rehabilitation programmes is advancing at a slow pace, and maintaining consistency in engagement with these solutions presents challenges.^9^ An important criteria for success are integration with care models, deeper patient engagement and patient-centred care.^10^ As a complement to simple telerehabilitation solutions self-managed digital rehabilitation programmes for improving motor function can provide more intensity, adherence, and progress to rehabilitation^9,11,12,16^ and is preferred as it is conducted in real-life environments for better skill transfer.^19^ Also, it can improve self-care and empowerment for the stroke survivors.^8,9^ Digital rehabilitation coupled with telesupervision allows for thorough monitoring and personalized feedback on patients’ performance. It also provides the opportunity to address challenges and rectify ongoing issues related to patient engagement in digital training.^14^ The expansion of digital home rehabilitation programmes also provides an opportunity to reframe the role of therapists with motor practice self-managed at home and therapist time allocated to remote supervision, monitoring, and motivation. In this flipped model of care, self-managed rehabilitation programmes require therapist support through supervision to attain successful behavioural change,^20^ and to handle low adherence to self-rehabilitation.^6^

Telerehabilitation is an emerging area of research, yet the effectiveness is conflicting, and the mode, delivery, content, and processes of digital services vary.^21^ Among existing systematic reviews on digital stroke rehabilitation for motor function, some show results in favour of digital rehabilitation,^22–26^ and others show no difference compared to conventional care^27–29^ (Supplemental File 1 – Existing and ongoing systematic reviews and gaps in these reviews). In these studies, the digital rehabilitation programmes are either entirely self-managed or situated within health institutions, meaning that programmes require time-intensive therapist time.^20^ Despite the extensive range of digital solutions for stroke telerehabilitation, there are currently few systematic reviews examining the effects of a more stable home setting, uniform technological applications, and specific rehabilitation interventions on the improvement of both motor and self-reported outcomes.

Although the research and clinical use of digital services in home settings are progressing^9,11,12,16^ only one study has reviewed interventions conducted at home.^28^ This 2015 review reported equal benefits compared to conventional care but noted variable outcome measures, technologies and methodological issues of various study designs.^28^ Additionally, in all the home interventions provided for stroke survivors none were coupled with telesupervision. Despite research on self-managed digital rehabilitation programmes outcomes that are self-reported are rarely addressed, such as quality of life (QOL).^30^ To date, only one study reviewed QOL for post-stroke survivors and further research is advocated.^31^ The use of self-reported outcomes in motor function rehabilitation for stroke are clinically important for considering the individual strategies towards their attitudes. It provides insights into the subjective impacts of their stroke and establish confidence to adopt healthy behaviours and accelerate rehabilitation.^32^ Another advantage is that self-reported outcomes can be remotely measured in the digital rehabilitation programmes, which might reduce the frequency of the outpatient visits required.^33^ Given the rapid development and limited evidence syntheses, this systematic review and meta-analysis evaluated the effectiveness of digital home rehabilitation and supervision in improving motor ability of upper limb and static balance (primary outcomes), in addition to stroke-related QOL and self-reported arm function (secondary outcomes) for stroke survivors.

## Materials and methods

### Design

This review was guided by The Cochrane Handbook for Systematic Reviews of Interventions^34^ and followed the Preferred Reporting Items for Systematic Reviews and Meta-analysis.^35^ The review protocol was registered in the International Prospective Register of Systematic Reviews (PROSPERO) (CRD42022331724).

### Eligibility criteria

Inclusion- and exclusion criteria based on PICOS format were used to identify studies.

The study population comprised home-dwelling stroke survivors (age ≥ 18 years) with no exclusion based on the type of stroke. Children under 18 were excluded from this review as the incidence of stroke in this population is low, and additional challenges and barriers to paediatric delivery of digital rehabilitation programmes might exist. Studies with participants in late sub-acute or chronic phase (more than 3 months after stroke) were included in accordance with a framework for post-stroke timepoints.^36^ The stroke classification was defined due to this review's focus on a population often challenging to target after hospital discharge, where most rapid brain repair processes in the first weeks-to-months post-stroke have passed and thus many fail to progress.^36^ Studies including mixed neurological populations combined with other conditions than stroke were excluded unless results of the stroke cohort could be extracted separately.

The interventions included digital rehabilitation programmes combined with telesupervision as communication technology (e.g., chat, video-consultation) used on the web or application on the phone conducted in the home setting or simulated home setting. For inclusion the intervention needed to target digital rehabilitation programmes aimed at improving activity and/or mobility, rather than simple communication with health care professionals (e.g., just video conference or education). In including supervision coupled with home-based rehabilitation programmes we attempted to control for amount of time with therapists allocated in conventional care. The intervention also had to be intended for participants with stroke and not just their caregivers. Advanced technologies of robotic technology, exoskeleton-based systems or electrical stimulation systems were not excluded from this review if they were delivered via telerehabilitation technologies and fulfilled additional inclusion criteria. Yet, prediction systems (e.g., risk factor assessment) outside the scope of this review targeting rehabilitation interventions were excluded.

Studies with a comparator of non-digital conventional care provided either at clinic-based settings or as home training was included. Among the post-stroke population, the accessible rehabilitation programmes are often delivered in outpatient settings after discharged to home and often with a combination of interventions for self-management and therapist interaction.

Primary outcome of interest was motor ability. Additionally, studies reporting one or more secondary self-reported outcome(s) were included but was not restricted to report additional outcomes. All study designs were searched, but this review included only randomized controlled trials (RCTs) or pilot RCTs. This is because the review aimed to assess treatment effects, and focusing primarily on randomized trials was considered feasible for the intervention of interest.^34^

### Search strategy

Six electronic databases of MEDLINE, Embase, CINAHL, The Cochrane Library, Scopus and Web of Science were searched from the inception of literature to 6 February 2024. An extensive search strategy was developed with experienced medical librarians consisting of various search terms related to specifications of stroke AND a broad inclusion of digital services, e.g., ‘telerehabilitation’, OR ‘ehealth’ OR ‘mhealth’, OR ‘Internet’, OR ‘fitness trackers’, OR ‘video games’. To specify the intervention towards home settings, a broad list of search terms on home was combined with the search. Subject heading trees were examined for any accurate subject headings to be added. RCT search syntaxes were also used. Secondly, grey sources such as websites from Google, European mHealth Hub, World Stroke Organization, National Stroke Associations, and other databases such as Open Grey, ProQuest Dissertations and Clinical Trials were searched for relevant trials. Additionally, we searched reference lists of single studies and systematic reviews. The search retrieved many protocols and authors of all retrieved protocols were contacted for available data and published results. Full search strategies can be found in Supplemental File 2 – Search strategies.

### Selection process

Search results were exported and managed using EndNote 20 and then Rayyan as a screening tool. Duplicates were removed electronically and then manually. Two investigators (AHM and ZAT) independently reviewed the titles, abstracts, and then independently screened full texts for eligibility and performed data extraction. Both the initial and full-text screening processes were pilot-tested and discussed before further screening. Discrepancies were discussed with a third reviewer (HGH) in the full-text screening to reach consensus on the eligibility of the studies.

### Data extraction and management

Two independent investigators (AHM and HXO) conducted data extraction using a pilot-tested template developed specifically for this systematic review inspired by Cochrane's data collection form for intervention reviews for RCTs only^34^ (Supplemental File 3 – Data Extraction Form and Risk of Bias Tool). Key data on study eligibility, bibliographic data (author, publication year, and country), characteristics (objective, design, population size, and characteristics), intervention and comparison descriptions, all outcome measures and validity and reliability of the instruments used, and descriptive and statistical results were extracted. Authors were contacted by email if important data was missing. Finally, the study data details of the included studies were summarized in a table of characteristics (Table 1).

**Table 1.** Characteristics of the included studies (population and intervention) (*n* = 13).

Study ID/design	Country	Sample size	Attrition	Adherence	Stroke onsets mean (SD)	Stroke subtype mean (SD)	Intervention details IG CG	
Adams, 2023 RCT	USA	Total (*n* = 21) IG (*n* = 12) CG (*n* = 9)	IG: 3 didn’t complete intervention and assessment	IG: recommended 24 h of digital system. Median total hour 13.7 (1,2–27,7). 34/36% synchronous telehealth visits	Total: 14 months IG: 10 (3–420) months CG: 25 (3–200) months	NR	Glove rehabilitation application for stroke (GRASP) virtual reality system -Saebo VR software -Kinect-based motion capture to track the patients’ movements -IADL tasks (shopping, clothing, cooking) -videoconference to give feedback and adjustments	Usual and customary care
Allegue, 2022 FRCT	Canada	Total (*n* = 11) IG (*n* = 6) CG (*n* = 5)	IG: 2/11 (18%) CG: 1/11	IG: 50% CG: 20%	Total: NR IG: 8 (2) years CG: 9,8 (3) years	IG: 67% ischaemic, 33% haemorrhage CG: 60% ischaemic, 40% haemorrhage	VirTele programme (Jintronix Exergames) - 5 games for upper extremity training -Kinect camera - Clinical-adjusted difficulty Reacts app as videoconference	Home training programme -GRASP (strengthening and ROM) -Different equipment from daily life
Chen 2021, RCT	China	Total (*n* = 80) IG (*n* = 40) CG (*n* = 40)	NR	NR	Total: NR (discharged home)	NR	WeChat group (Neurology continued nursing patients”) combined with Follow-up management system. -online lectures -communicate online for rehabilitation expert team and stroke patients -management, reminder function, data collection, tracking, push health education videos	Handbook of health knowledge -physical exercise rehabilitation plan -information about stroke -other educational materials Telephone follow-up Out-patient follow-up 1- and 3-months post-discharge Family follow-up
Conroy 2020, PRCT	USA	Total (*n* = 29) IG (*n* = 15) CG (*n* = 14)	3/29 (10%) in both groups	80% (satisfaction survey)	Total: 6,25 (5,7) years IG: 75,0 (68,4) months CG: 63,24 (37,3) months	IG: 80% ischaemic 20% haemorrhage, CG: 71% ischaemic 29% haemorrhage	E-visits by supporting patient–provider messaging Study-specific exercise video access in portal + Feedback, encouragement, and exercise video link in portal	Clinic-based rehabilitation
Cramer, 2019 RCT	USA	Total (*n* = 124) IG (*n* = 62) CG (*n* = 62)	Total (*n* = 10) IG (*n* = 3) CG (*n* = 7)	IG: 98.3% CG: 93.3%	Total: NR IG: 132 (65) days CG: 129 (59) days	IG: 87,1% ischaemic 12,9% haemorrhage CG: 83,9% ischaemic 16,1% haemorrhage	18 supervised sessions (70 min videoconferencing) -12 games, stroke education, therapist's review of electronic data -15 min daily arm exercises (bank of 88 exercises) on web or videoconferencing 18 unsupervised treatments (content as supervised sessions) -jeopardy game + feedback	18 supervised sessions (70 min clinic-based sessions) -15 min per day of arm exercises (bank of 88 exercises) -stroke education 18 unsupervised (printed homework) -multiple choice questions in paper booklets
Gauthier, 2022 RCT	USA	Total (*n* = 193) IG (*n* = 51) CG (*n* = 44) Other (*n* = 98)	10% (treatment) 25% (follow-up) (occurred in CG)	IG: 37% fully adherent (≥15 h) median: 12 h.	Total: 5 years IG: 3,4 (5,1) years CG: 5,8 (8,1) years	Stroke of any aetiology	15 h of unsupervised CI therapy (self-managed videogame motor practice at home + 6 supervised video consultations (2*6 h) + 4 visits (5 h) over 3 weeks of clinic-based treatment of behavioural change. (Goals, motivational interviewing)	5 h (4 visits) clinic-based sessions (motor training, neuromuscular re-education, functional training, progressive strengthening Home programme
Hernandez2022 RCT	Canada	Total: (*n* = 53) IG: (*n* = 26) CG (*n* = 27)	4% in CG.	IG: 21.5 sessions duration of 527 min. (50% range of 310–673 min. CG: 12 sessions	Total: NR IG: 5.3 years (1.5–8.1) CG: 4.4 years (2.2–7.4)	IG: 14% ischaemic, 7% haemorrhage, 5% unknown CG: 10% ischaemic, 7% haemorrhage, 8% unknow	Remotely supervised home-based programme (Jintronix system) (adjusted 1–2 a week) Microsoft Kinect sensor camera for movement tracking without the need for a handheld controller	Manual for a standardized exercise programme, the GRASP
Kizoni 2013 RCT	Israel	Total (*n* = 20) IG (*n* = 10) CG (*n* = 10)	IC: 1 CG: 1	NR	Total: NR IG: 39,7 (23,2) months CG: 39,4 (19,9) months	NR	Quasi-home based telemotion system (Gertner system) -video-capture VR system (level of difficulty, results, and performance/feedback) - monitoring for compensatory movements of arm and trunk -evaluation protocol to assess ROM of shoulder and elbow -instructional demo	Structured, self-training upper extremity exercises at home
Krpic, 2013 RCT	Slovenia	Total (*n* = 26) IG (*n* = 6) CG (*n* = 11) BG (*n* = 9)	NR	NR	Total: NR Recruited at hospital discharge	IG: 66,7% ischaemic, 33,3% haemorrhage CG: 77,4 ischaemic, 22,6% haemorrhage	Home system (Tele-virtual reality balance training -Balance training weight bearing -3-axial tilt/inclination sensor -Panda3D VR -real-time data exchange -Therapist user interface	Conventional balance training -4 weeks, 5 days a week, 45 min Manual assistance Balance group (BS) on balance frame -4 weeks, 5 days a week, 20 min
Piron 2009 RCT	Italy	Total (*n* = 36) IG (*n* = 18) CG (*n* = 18)	NR	NR	Total: NR IG: 14,7 (6,6) months CG: 11,9 (3,7)	44% ischaemic stroke in left region, 55% ischaemic stroke in right middle cerebral artery	VR rehabilitation system with a 3D motion tracking -5 virtual tasks, comprising simple arm movements for training -patients moved the real object following the trajectory of the corresponding virtual object on the pc in accordance with the requested virtual task -Tracking of movement and correct trajectory pre-recorded virtually (virtual teacher). -tasks’ exactness through the videoconferencing system	Conventional physiotherapy in the local health-district. Specific exercises for the upper limb with a strategy of progressive complexity
Salguiro 2022 (extension of) RCT	Spain	Total (*n* = 49) IG (=20) CG (=29)	IG (*n* = 7) CG (*n* = 4)	IG: 4/13 of completers regularly used the App CG: 12.5% of the exercise programme proposed	Total: NR (hospital discharge) subacute	IG: 75.86% ischaemic, 24.14% haemorrhagic CG: 84.21% ischaemic, 15.79% haemorrhagic	Farmalarm App as a telerehabilitation tool to guide home-based core stability exercises. Neurologic physiotherapist available for producing exercises and available for video calls	Conventional therapy. Therapeutic techniques (muscle stretching, passive and active mobilization of the affected body segments, balance exercises and gait training were included as conventional care
Toh, 2024 FRCT	China	Total (*n* = 12) IG (*n* = 12) CG (*n* = 12 Cross-over	CG: 3 after first round of intervention and before control	IG: 78.25% CG: 70.58%	Total: 85.00 months (64.23) IG: 42.50 (36.82) CG: 127.50 (58.18)	IG: 83.3% right region CG: 50% right region	Smartreminder wristwatch and telerehabilitation app -exercise videos -visual feedback on performance -vibrations and audible signals to remind the wearer to perform the prescribed exercises. -sensors to detect and record range of motion (ROM)	Dose-matched home exercises via handout. One 30-min weekly in-person therapy consultation session
Uswatte, 2021 RCT	USA	Total (*n* = 24) IG (*n* = 12) CG (*n* = 12	IG: 2 (+3 in follow-up) CG: 2 (+3 follow-up)	NR	Total: NR IG: 2.5 (1.7–3.4) years CG: 2.4 (1.6–3.1) years	IG: 58% right region CG: 42% right region	35 h Tele-AutoCITE -CIMT content like CG (based on needs) 10 tasks -remote supervision -built-in sensors for monitoring/training records - software automatically directed the participant -contract of activities (wearing a mitt on less-affected arm) outside Tele-CIMT	35 h of clinic-based CIMT group with face-to-face treatment in clinic

Abbreviations: FRCT: feasibility randomized controlled trial; RCT: randomized controlled trial; PRCT: pilot randomized controlled trial; BL: baseline; IG: intervention group; CG: control group; NR, not reported; GRASP: Graded Repetitive Arm Supplementary Programme; ROM: range of motion; ICN: Internet continuing nursing; CI/CIMT: constraint-induced (movement therapy); IADL: activities of daily living.

### Methodological quality assessment

Using the Cochrane Risk of Bias Tool 1 and 2^34^ (Supplemental File 3) two reviewers (AHM and HXO) independently assessed the methodological quality of the included studies for selection, performance, detection, attrition, and reporting bias. The studies were assessed for low, unclear, or high risk. Justifications for the assessments were documented in the data extraction form.

### Synthesis and statistical analysis

Mean and standard deviations (SDs) were extracted for continuous variables. Differences between the effect of the intervention and the control on outcomes were calculated as standardized mean differences (SMDs) with 95% confidence intervals (CI) in RevMan due to different scales being used to report the same outcome. A weighted average of the intervention's effectiveness in each study was calculated as Hedges’ *g*, which adjusts for small sample bias, where 0.2, 0.5 and 0.8 corresponds to low, moderate, and high effect estimates respectively.^34^ Medians were converted to mean by using equation (37), where *a* and *b* represents the minimum and maximum value respectively, and *m* represents median:

$$\tilde{X}\approx \frac{a+2m+b}{4}$$

SDs were calculated by subtracting the maximum value from the minimum value and dividing it by 4.^37^ Similar studies reporting similar outcomes were pooled for meta-analysis. Review Manager 5.4 (RevMan 5.4) was used to perform meta-analysis for all outcomes and risk of bias assessment summary and graphs. Outcomes measured at the timepoint closest to the post-intervention and timepoints most comparable for the included studies in meta-analysis were selected. For multiple armed RCTs with multiple comparison groups we chose the control group most similar to conventional therapy and other pooled control groups of other included studies. Statistical heterogeneity was assessed using the Chi-square test (χ^2^ test) and *I^2^* statistic. If *I^2^* ≤ 40% it was interpreted as heterogeneity that might not be important; however, 30%–60%, 50%–90%, and 75%–100% indicated moderate, substantial, and considerable heterogeneity, respectively.^34^ Sensitivity and subgroup analysis were performed if heterogeneity was detected. The random-effects model with the generic inverse variance method was used to allow for some within and between variability of studies. The funnel plot and test for funnel plot asymmetry was not used for assessing publication bias as no examined outcome was reported by more than 10 studies.^38^ The Grading of Recommendations, Assessments, Development and Evaluations (GRADE) tool was used to assess the overall quality of the evidence.^39^

## Results

### Study selection

**Figure 1.**
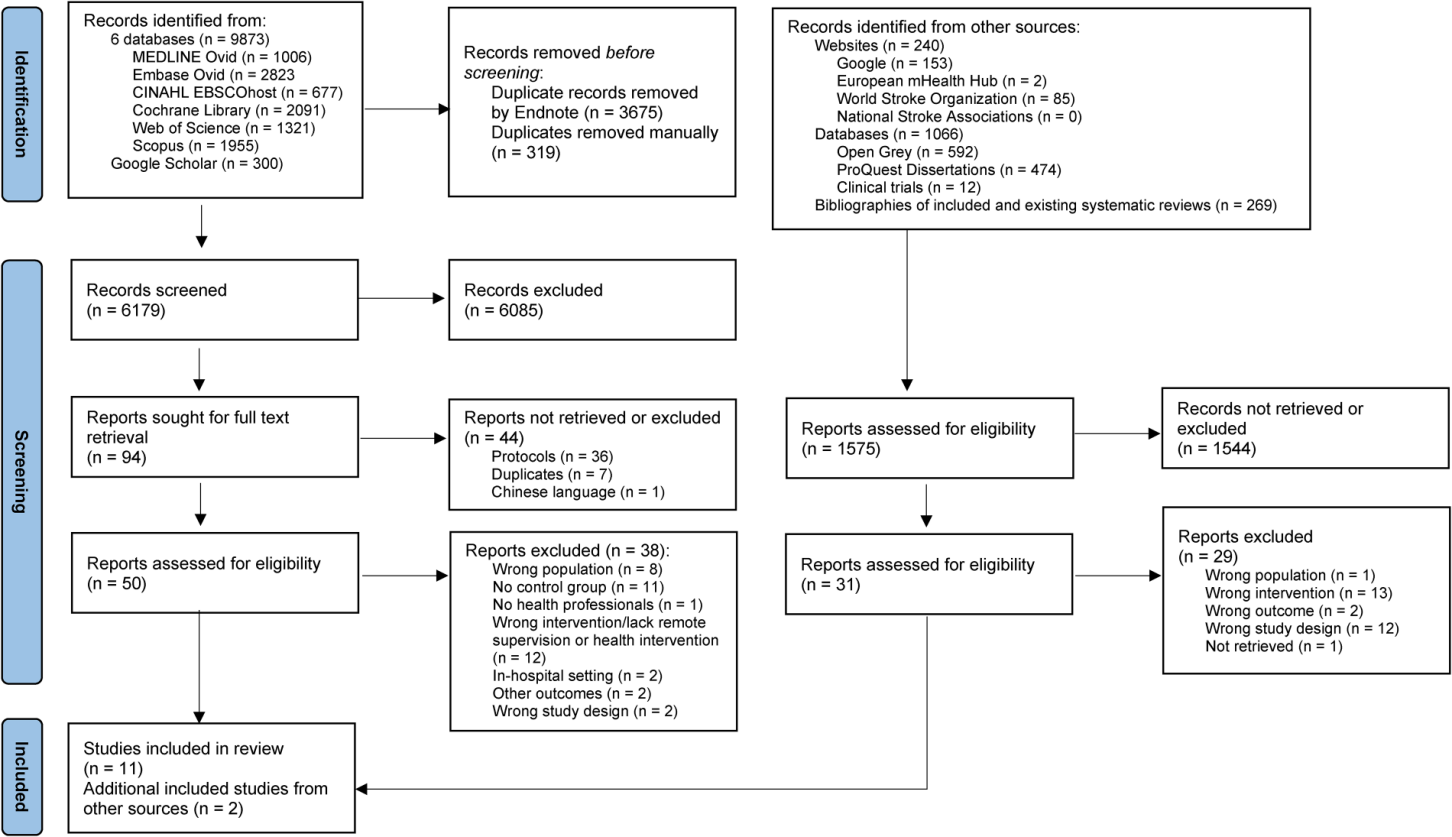
PRISMA flow chart summarizing the search process and outcomes.

A total of 9873 trials were identified through search in six databases and 1575 additional records through other sources from inception of the databases to 6 February 2024. After removal of duplicates 6179 were screened by titles and abstracts. Among these, 6085 were excluded, 94 were sought for full-text retrieval and 50 assessed for eligibility based on different arguments (see Figure 1 PRISMA chart and Supplemental File 4 – Included and excluded articles). Finally, 13 RCTs were included in this review.

### Study characteristics

A total of 571 stroke survivors, 290 in intervention group and 293 in control group, from 13 RCTs from seven different countries were included in this systematic review. For each included study we identified the sample size (with attrition and adherence rate), stroke onset and subtype, descriptions of the technology and intervention details, regime, outcomes, timepoints and results (see Table 1 and Table 2 of included studies).

**Table 2.** Outcomes and results of included studies (*n* = 13).

Study ID	Digital service technology	Regime	Outcome measures	Timepoints	Results
Adams	VR home exercise programme -glove application -videoconference (async+sync)	8 weeks 45 min, 4 times/week	Motor ability of upper limb FMA, WMFT, BBT, MAL	T0: Baseline T1: 8 weeks	+++FMA: 8,6 greater change in IG compared to CG. ++WMFT-time: IG: 0,82 estimated mean ratio. CG: 0,68 estimated mean ++WMFT-ability: 0,53 adjusted greater change in IG compared to CG. ++BBT: 0,34 adjusted greater change in IG than CG. +++MAL-amount: 1,31 greater adjusted change in IG than in the CG.
Allegue	VR/Exergames + app for videoconferences (sync+async)	8 weeks 3 times/week in 2 weeks 2 times/week following 2 weeks 1 time/week remaining 4 weeks	Motor ability of upper limb FMA, SIS-16, MAL_30, TSRQ-1	T1: baseline T2: 8 weeks (2 months) T3: 4 months T4: 6 months	^ FMA. 50% improvement within IG and CG. ++MAL-30. IG: 100% improvement. CG: 80%. -SIS-16 hand function. IG: 0%. CG: 100% improvement. ++SIS-16 mobility. IG: 50% improvement. CG: 40%. ++TSRQ. 75% increase in IG, only one in CG.
Chen	ICN platform (app+follow-up management system) (async)	6 weeks	Motor function MAS, EQA, SSC, SS-QOL	T0: baseline T1: 6 weeks T2: 3 months	+++MAS. IG: 8 change from T0 to T1. CG: 3 change from T0 to T1. +++EAQ. IG: 11 change from T0 to T1. CG: 4 change from T0 to T1. +++SSC. IG: 2 change from T0 to T1. CG: 1 change. +++SS-QOL. IG: 35 change from T0 to T1. CG: 10 change from T0 to T1.
Conroy	Web-based patient portals (secure messaging system+web) (async)	6 weeks	Motor ability of upper limb WMFT, SIS, eHealths, WBLSES	T0: baseline T1: 6 weeks	-WMFT. IG: −1.2 s change from T0 to T1. CG: -3,3 s change. A lower WMFT score indicates more improvement. ++SIS. IG: 9.1 score. CG: -2,3. ++eHealths. IG: 8% score change ++WBLSES IG: 2% change score change
Cramer	Web-based gaming input devices + videoconference (sync+async)	6–8 weeks 36 sessions total	Motor ability of upper limb FMA, Box blocks, SIS	T0: baseline T1: 1-month post-intervention (3 months)	+FMA. IG: 7.86 unadjusted change from T0 to T1. CG: 8.36 change. Noninferiority Adjusted mean change was 0.06. points larger in IG. +Box blocks. IG: 9.5. CG: 8.8. Noninferiority. +SIS hand motor domain. IG: 23.7. CG: 29.2
Gauthier	Telegaming (commercially interactive video gaming, smart watch) + videoconference (async+sync)	3 weeks	Motor ability of upper limb MAL, WMFT	T0: baseline T1: 1 week T2: 5–7 months post-treatment	+++MAL. CG 1,0 (95% CI 0,8 to 1,3) for IG. No-inferiority to CI (other intervention group). ++WMFT. IG: 53% change CG: 45% change
Hernandez	VR/exergames + adjustments by therapist (async)	1 month 5 times/week 20 min per session	Motor ability of upper limb FMA, MAL, SIS	T0: baseline T1: 1 month T2: 2–3 months	+FMA. IG: 2,5 change. CG: -0,52 -SIS: IG: -3,07 change. CG: 2,2 change -MAL.
Kizoni	Video games 3D video capture Kinect camera-based system Support (async+sync)	4 weeks 12 sessions 45 min	Motor ability of upper limb FMA, CAHAI-7, MAL	T0: baseline T1: 1 month T2: 2 months	+FMA: IG: 10.7. CG: 14.0 -CAHAI-7 +++MAL amount. IG:12.3. CG: 2.8 +++MAL quality. IG:15.5, CG: 0.9
Krpic	VR telerehabilitation and balance training -standing frame -videoconference (async+sync)	3 weeks 5 days/week 15 min.	Motor function/static balance BBS, TUG, 10MWT	T0: Baseline T1: 3–4 week	+BBS: IG: 15% change T0 to T1. CG: 54% change +10 MWT: IG: 25,7%. CG:16,5% +TUG. IG: 29,9%. CG: 20,4%.
Piron	Web based VR+videoconference +(Polhemus) 3D motion tracking system via a magnetic receiver attached to a real object (async+sync)	1 month 5 days/week 1 h/day	Motor ability of upper limb FMA, MAS*, ABILHAND	T0: 1 month before intervention T1: 30 days (start of intervention) T2: 60 days after T3: 90 days after	+++FMA. IG: 53,6 score. CG: 49,5 score at T2. Follow-up phase (from T2 to T3) both groups benefit +++MAS*. IG: 1,7 change. CG: 1,0 ++ABILDHAND. Statistically significant change between groups was seen at 3 assessment times (not follow-up)
Salguiro	App for core exercises + video calls in app + telephone (sync+async)	5 weeks 2,5 h sessions/week	Motor function/static balance TIS, FIST, BBS, PASS, 3-BBA	T0: baseline T1: 3 months	++TIS: IG: 0,95 change. CG: 0,65 points ++FIST. IG: 24% change. CG: 1,45% ++BBS. IG: 18,15% change. CG: -5,57%. ++PASS. IG: 9,36% change. CG: 0,36% ++ 3-BBA. 16,29% change in IG vs 1,91% in CG (p = 0,49)
Toh	Wristwatch + telerehabilitation app -motion tracking -remote feedback and monitoring - 30-min consultation with the therapist weekly (sync+async)	4 weeks 5 days/week 45 min	Motor ability of upper limb FMA, ARAT, active ROM, MAL	T0: baseline T1: post-treatment at 4 Weeks T2: after a 3-week washout period at crossover before beginning the second intervention T3: after the second intervention	-FMA: IG: 0,08 points change. CG: 0, 75 +ARAT: IG: 2 points change. CG: 1,09 points change. +++active flexion IG: (8.9±12.7, p = 0.022) and abduction (12.2 ±14.3, p = 0.018) -MAL: IG: 0,06 points change. CG: 0,15 points change.
Uswatte	Tele-CIMT training -CIMT upper extremity training -Remote supervision async+sync	10 days 3,5 h/day. 37.5 min on tasks 142.5 min rest	Motor ability of upper limb MAL, WMFT, POS	T0: baseline T1: 11 day (post-treatment) T2: 1 year	+MAL: IG: 3,7 points change. CG: 3.7. +WMFT: IG: 34 change in repetitions per minute. CG: 33 repetitions. ++POS. IG: 6.7. points change. CG: 6.4

Abbreviations: FRCT: feasibility randomized controlled trial; RCT: randomized controlled trial; PRCT: pilot randomized controlled trial; BL: baseline; IG: intervention group; CG: control group; NR, not reported; GRASP: Graded Repetitive Arm Supplementary Programme; ROM: range of motion; ICN: Internet continuing nursing; CI/CIMT: constraint-induced (movement therapy); IADL: activities of daily living; FMA: Fugl-Meyer Assessment; WMFT: Wolf Motor Function Test; BBT: Box and blocks; MAL: Motor Activity Log-30; SIS: Stroke Impact Scale; TSRQ-15: Treatment Self-Regulation Questionnaire-15; EQA: Functional Exercise Compliance Test; MAS: Motor Assessment Scale; SSC: Self-efficacy; SS-QOL: stroke-specific quality of life; SIS: Stroke Impact Scale; eHealths: Electronic Health Literacy Scale; WBLSES: Web-Based Learning Self-Efficacy Scale; CAHAI-7: Chedoke Arm and Hand Activity Inventory; MAS*: modified Ashworth Scale; TIS: Trunk Impairment Scale; FIST: function in sitting; BBS: Bergs Balance Test; PASS: Postural Assessment Scale for Stroke Patients; 3-BBA: Brunels Balance Assessment (stepping); POS: Participant Opinion Survey; TUG: Timed Up and Go Test; 6MWT: 6 min walk test; MBI: Modified Barthel Index Scale; +++: statistically significant effect; ++: greater improvement in intervention group than control but between group difference not significant; +: significant improvement in both groups but between group difference not reported or not significant; -: no reported change in the group(s) or improvement just in control group; x: effect-related data not shown; ^: within-group improvement not significant.

The sample size of the studies varied from 11^40^ to 193^20^ depending on whether it was a feasibility or multi-site RCT. Among the trials reporting attrition, the mean rate was approximately 17% and below the considered attribution rate of 20%, which raise concerns about the study validity.^41^ There was a large variation in reported adherence from 30% to 98%. All trials included participants after sub-acute or chronic stroke mostly reported in years, except one trial of sub-acute participants of approximately four months onset. The remaining trials included participants with a mean stroke onset of 3.6 years. Across trials, the majority of the included stroke participants had an ischaemic subtype. All included studies targeted motor rehabilitation, with three studies^20,42,43^ also targeting digital stroke education, such as stroke prevention, risk factors and stroke knowledge. Most of the studies targeted upper limb motor training,^13,15,19,20,40,42,44–47^ two studies aimed for static sitting or standing balance,^33,48^ and one trial aimed for motor function without further description of intervention aim.^32^ We categorized the included trials into types of intervention, technology, and control (Supplemental File 5 – Categorization of studies). Most of the included trials used virtual reality (VR) or exergaming as an intervention,^13,20,33,42,44–47^ four used a stand-alone app or platform^15,19,32,48^ and one used app and VR in combination.^40^ One study used a tele-based gaming with constraint-induced movement therapy (CIMT) on a workstation.^47^ Also one of the VR trials had the features of CIMT integrated into the solution.^20^ The four app solutions contained supervision of educational videos, remainder functions, and data tracking. Among these one app was connected to a wearable with sensor to detect range of motion.^19^ Among the gaming programmes most studies used customized rehabilitation software (e.g., Jintronix system, Gertner system and Saebo software), only one used more commercial gaming-based solutions.^42^ Among the gaming-based solutions some used connected devices; one used PlayStation Move controller [Sony] or trackpad, one used magnetic receiver attached to real objects, one used task stations with built-in sensors, one used a standing frame with a build-in 3-axial tilt/inclination sensor and another a Saebo VR-glove. Five studies also used motion capture tracking, such as the Kinect system.^13,40,44–46^

All trials contained interventions combined with telesupervision, where 10/13 used a combination of asynchronous (data storing) and synchronous communication (e.g., videoconferences) and 3/13 only used asynchronous communication via chat.^13,15,49^ Additionally, many rehabilitation systems monitored the patient's data and made asynchronous adjustments in accordance with performance. The aim of the videoconferences were to communicate and observe participant's performance, adjustments on progressive level and intensity, reviewing treatment plans based on needs and goals, communication around barriers in daily life and general encouragement to use the system. Two studies also used motivational interviewing^20,40^ and two other studies used a behavioural contract as behavioural interventions.^42,47^

Total number of weeks of treatment varied from two weeks to a maximum of eight weeks with a mean duration of 5 weeks. The daily duration of the intervention varied from 15 min to 3.5 h. In the more time-consuming CIMT programmes the duration were frequently set to 45 min. Almost all trials had timed-matched controls, and many trials had dose-matched controls. One trial had longer duration and dose in the control group of a conventional balance training programme. The control groups followed programmes either as home training (*n* = 4) or in clinic-based settings (*n* = 4), or where half of the intervention was conducted at home and the other in a clinic-based setting (*n* = 3). Two studies lacked descriptions of the setting and content. The content and intensity in the control groups varied. Whereas some participants in the control group were provided with handbooks of knowledge and exercise, others followed intensive programmes as conventional CIMT, Graded Repetitive Arm Supplementary Programme and task-specific training.

All trials assessed motor ability, either upper limb motor ability through The Fugl-Meyer Assessment of Motor Recovery after Stroke (*n* = 7), Wolf Motor Function Test (*n* = 3), Motor Assessment Scale (*n* = 1) or Berg's Balance Test (*n* = 2). Eleven studies also assessed self-reported outcomes as the QOL by Stroke Impact Scale (*n* = 3), Stroke-Specific QOL (SS-QOL) Scale (*n* = 1), or self-reported arm function by using Motor Activity Log (*n* = 6), or ABILHAND (*n* = 1). Five studies reported more than one self-reported outcome, a combination of the above or other (see Table 2).

Most trials reported positive results in within-group differences except two trials, however three studies reported significant between-groups differences in favour of digital rehabilitation in motor ability outcomes. Yet, in self-reported outcomes five trials reported significant between-group differences or greater improvements in favour of digital rehabilitation.

### Meta-analysis

In three of the included studies mean differences and SDs could be extracted directly.^15,19,42^ In four studies, the mean difference was calculated by subtracting the post-intervention mean from the pre-intervention mean and a combined SD calculated manually.^20,33,46,48^ In two studies full datasets of outcome scores for every individual were provided and could be calculated in excel.^13,40^ In one study median and interquartile range were reported and had to be manually converted to mean difference and SDs.^45^

In two other studies mean difference could be extracted directly, but CIs were used. CIs had to be calculated to SDs by subtracting the upper CI with the mean and then dividing it by t-distribution to receive standard error and then multiplying the standard error with the square root of the number of participants.^44,47^ In one last study SD was not reported, only p-values, sample size and difference in means.^32^ To calculate SD we used RevMan's additional calculator and used numbers calculated from between groups by finding *t*-value, standard error and using standard error to find SD.^34^ Subgroup analyses were performed for the self-reported arm outcome by grouping data with target intervention and duration of intervention.

### Methodological quality and risk of bias assessment

**Figure 2.**
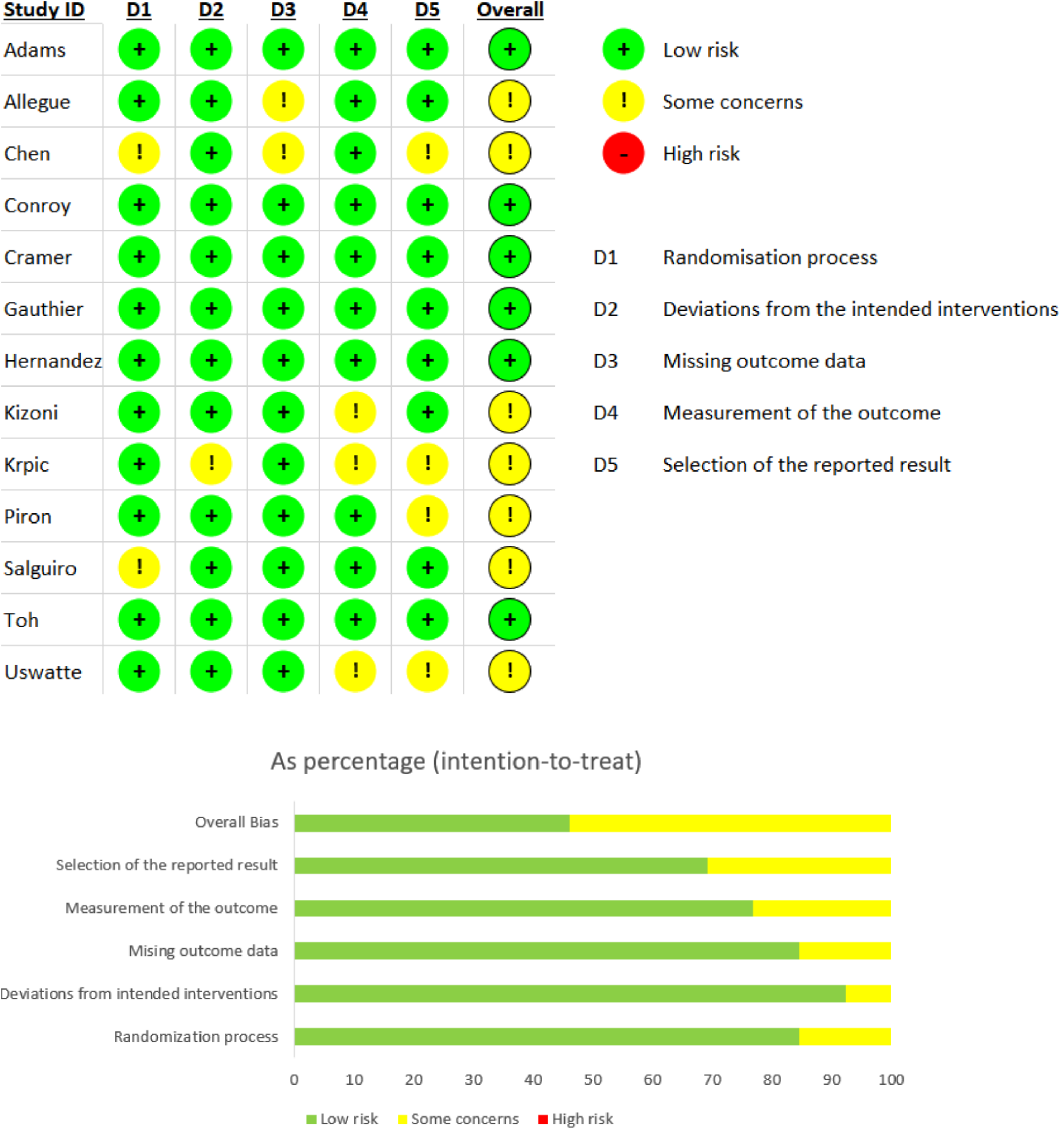
Risk of bias (RoB 2) summary of each included study and overall result (percentage).

Two reviewers independently assessed the methodological quality of the included studies by using Cochrane's Risk of Bias tool (RoB) 1 as the version integrated in Rev Man 5.4 (see Supplemental File 6) and the updated assessment of RoB 2 (see Figure 2). Generally, there were low risks across various items in RoB 2. In RoB 2, low risk of bias was consistent across the studies for randomization process, deviations from the intended interventions and missing outcome data, except in two trials with some concerns related to allocation sequence and lack of appropriate analysis used to estimate the effect of assignment to intervention respectively. More concerns were raised in three studies due to measurement of the outcome and selection of the reported result due to lack of blinded testers and pre-specified analysis plan that was finalized before unblinded outcome data were available for analysis. Almost every trial could not fulfil blinding of participants and personnel due to the nature of interventions, except in one trial where participants were masked to study hypothesis.

### Effects of digital home rehabilitation and supervision for post-stroke survivors

#### Primary outcome: Motor ability for upper limb and static balance

**Figure 3.**
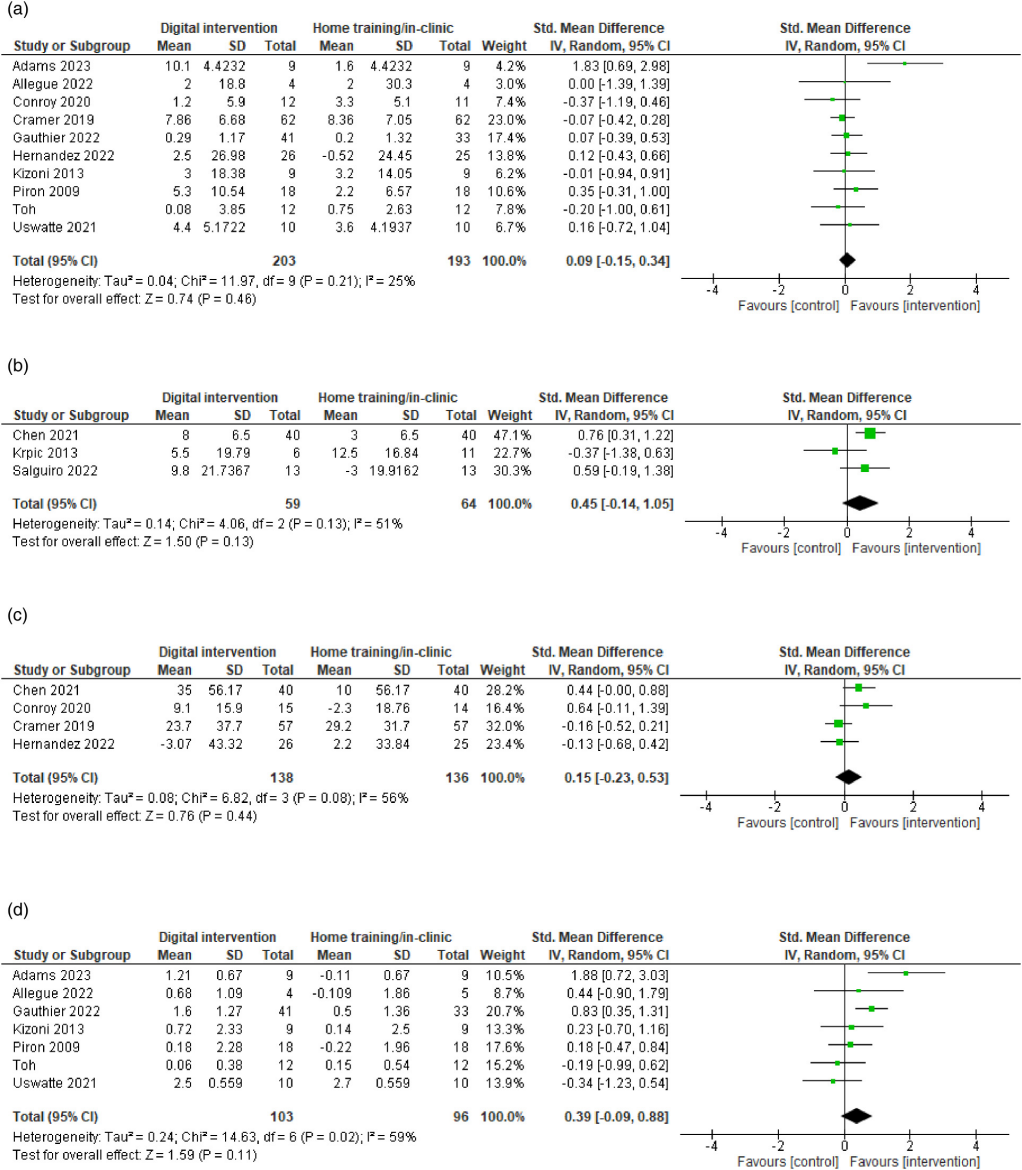
Forest plot: Meta-analysis for all outcomes. (a) Motor ability of upper limb (the higher score the better). (b) Static balance (the higher score the better). (c) Stroke-related quality of life (the higher score the better). (c) Self-reported arm function (the higher score the better).

All 13 studies measured motor ability outcomes, mostly for the upper limb. We separated the motor ability outcomes into two meta-analyses, one analysis for the motor ability of upper limb (*n* = 10), and one analysis for static balance (*n* = 3). One of the studies measured everyday motor function and was therefore pooled with other studies measuring static balance as outcome. Ten studies measuring motor ability of upper limb (Figure 3(A)) were pooled to conduct a meta-analysis with 396 participants. The effect size was 0.09, 95% CI [−0.15 to 0.34] and not significant (*p *= 0.46), yet with low heterogeneity (*I²*=25%). Due to low heterogeneity, no sensitivity analysis was performed. However, a subgroup analysis was performed to provide estimates of treatment effects based on the comparison types of control groups (either located in home, clinic-based or combined settings). There was no statistically significant subgroup effect (*p *= 0.50, analysis only presented in Supplemental File 7), suggesting that the difference between controls in home or clinic-based settings did not modify the effect of digital rehabilitation. However, a smaller number of trials and participants contributed data to the different subgroups, meaning that the analysis may not be able to detect subgroup differences.

**Figure 4.**
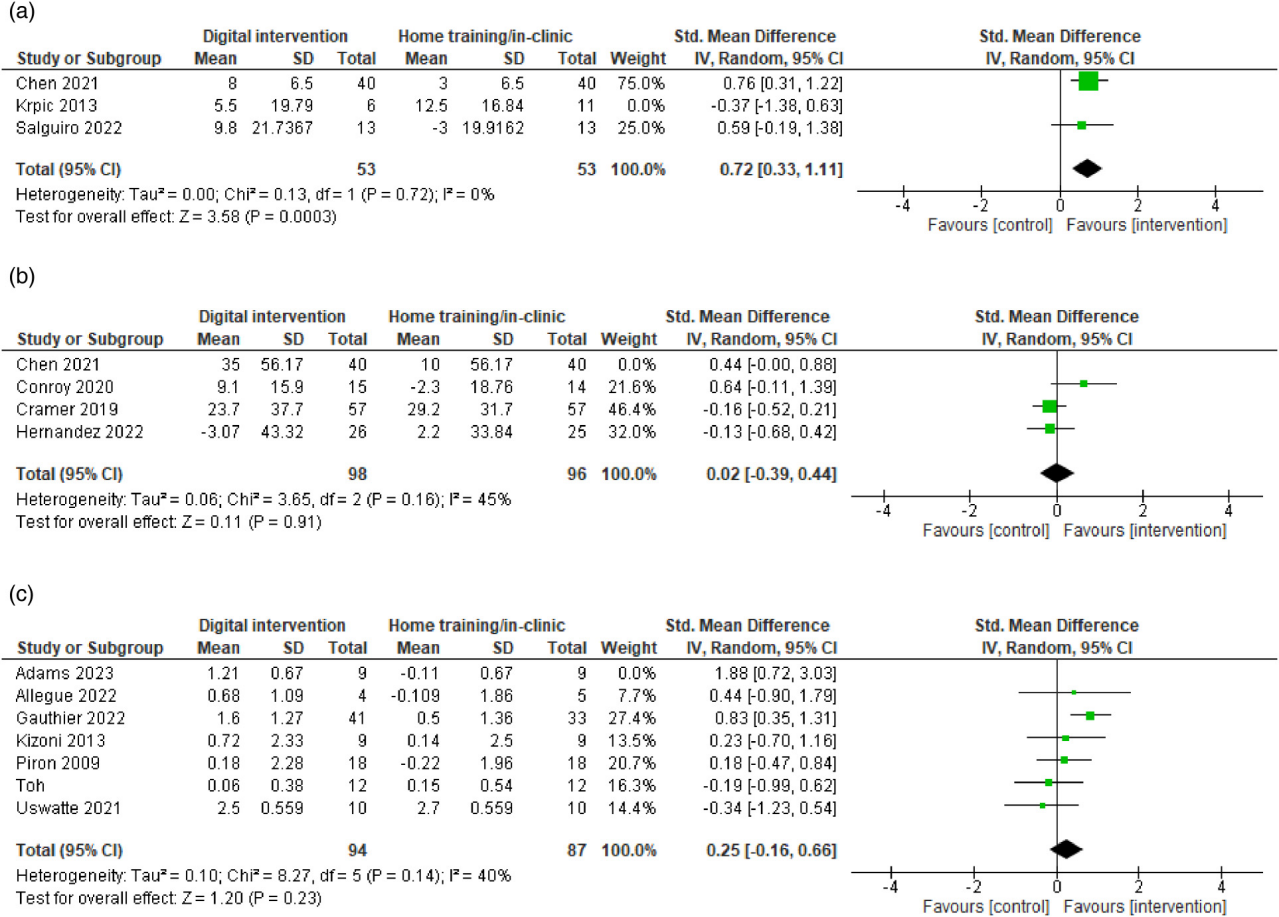
Forest plot: Sensitivity analysis for static balance, stroke-related quality of life and self-reported arm function. (a) Static balance. (b) Stroke-related quality of life. (c) Self-reported arm function.

Three studies measuring static balance (Figure 3(B)) were pooled in another meta-analysis with 123 participants. The effect size was 0.45, 95% CI [−0.14 to 1.05], and not significant (*p *= 0.13), with a moderate heterogeneity (*I²*=51%). It was decided to include studies with control groups with both home-based conventional care and in clinic-based settings as a comparison. In a sensitivity analysis, this was explored to see whether this decision affected findings and caused heterogeneity. Unlike the other studies, Krpic et al. compared their intervention with a control group receiving conventional therapy in a clinic with a larger training dose and duration and removal of this study reduced the heterogeneity to 0%. While a statistically significant effect (SMD = 0.72, 95% CI [0.33 to 1.11], *p *= 0.0003) appeared after sensitivity analysis (Figure 4(A)), this difference should be interpreted with caution as the sensitivity analysis was performed to explore heterogeneity and the removal of the study reduced an already small sample size.

#### Secondary outcome: Stroke-related QOL

Four studies measuring stroke-related QOL (Figure 3(C)) were pooled to conduct a meta-analysis with 274 participants. The effect size was 0.15, 95% CI [−0.23 to 0.53] and not significant (*p *= 0.44), with a moderate heterogeneity (*I²*=56%). Sensitivity analysis of the outlier did not change the heterogeneity significantly, but an exploration of quality-of-life instruments and exclusion of Chen et al. 2022 being the only one using different instruments than the other studies reduced the heterogeneity to 45% (Figure 4(B)). Again, subgroup analysis was performed to provide estimates of treatment effect for clinically relevant subgroups of patients in the control group, which showed subgroup differences were non-significant (*p *= 0.99, see Supplemental File 8). A small number of trials and participants contributed data to the different subgroups potentially hindering to detect subgroup differences.

#### Secondary outcome: Self-reported arm function

Seven studies measuring self-reported arm function (Figure 3(D)) were pooled to conduct a meta-analysis with 199 participants. The use of home-based digital motor rehabilitation had a small, but not statistically significant effect in improving self-reported arm function (SMD = 0.39, 95% CI [−0.09 to 0.88], *p *= 0.11, *I*^2 ^= 59%). Also, there was statistically significant moderate heterogeneity (*p *= 0.02, *I^2^*= 59%). This review included studies with a late subacute and chronic stroke population. In a sensitivity analysis, the first study with the outlier was removed and reduced the heterogeneity to 40%, without changing the effect estimate to a large degree (SMD = 0.25, 95% CI [−0.16 to 0.66], *Z* = 1.20, *p *= 0.23) (Figure 4(C)). We performed subgroup analysis to investigate sources of heterogeneity, to generate hypotheses and to provide estimates of treatment effect for clinically relevant subgroups of patients in the control group (Supplemental File 9). The test for subgroup differences indicated that there was no statistically significant subgroup effect based on comparison types of the control group (*p *= 0.35), instrument use (*p *= 0.40), or length of intervention (*p *= 0.20). However, it is interesting to note that the pooled effect estimate for instruments to measure the quality of arm use showed a significant effect (SMD = 0.68, 95% CI [0.27 to 1.09], *p *= 0.001). An uneven covariate distribution for particularly length of intervention time was present, meaning that the analysis was unlikely to produce useful findings.

In total, these results suggest that digital home rehabilitation and supervision compared to conventional care showed similar effect.

### Overall quality of evidence

Table 3 summarizes the overall quality of evidence in the GRADE assessment of the four different outcomes. In this review, the overall methodological quality is moderate, and the quality of evidence varies from low to high. In all GRADE assessments the outcomes scored low on risk of bias. The motor ability of upper limb showed the highest certainty based on low inconsistency, imprecision and similarity in population, interventions, and outcomes. The exception was some variations in content and dosage in the comparison group as explained earlier, which was evident in the three other evaluations of outcomes as well. The self-reported arm function showed the second highest and moderate certainty of evidence based on low imprecision and indirectness, but moderate inconsistency, which we downgraded due to a moderate heterogeneity explored in subgroup analyses. Static balance and stroke-related QOL showed respectively low and very low certainty of evidence due to downgrades in all or some criteria suggesting for the latter the confidence in the effect estimate was low and future studies may change the effect estimate.

**Table 3.** Overall quality of evidence assessment.

Certainty assessment							№ of patients		Effect		Certainty	Importance
№ of studies	Study design	Risk of bias	Inconsistency	Indirectness	Imprecision	Other considerations	Digital rehabilitation	home training/clinic-based	Relative (95% CI)	Absolute (95% CI)		
*Motor ability of upper limb*												
10	Randomized trials	Not serious	Not serious	Not serious	Not serious	None	203	193	-	SMD **0.09 SD higher** (0.15 lower to 0.34 higher)	⊕⊕⊕⊕ High	IMPORTANT
*Static balance*												
3	Randomized trials	Not serious	Serious^a^	Serious^b^	Serious^c^	None	59	64	-	SMD **0.45 SD higher** (0.14 lower to 1.05 higher)	⊕⊕⊕⊕ Very low	IMPORTANT
*Stroke-related quality of life*												
4	Randomized trials	Not serious	Serious^a^	Not serious	Serious^c^	None	138	136	-	SMD **0.15 SD higher** (0.23 lower to 0.53 higher)	⊕⊕⊕⊕ Low	IMPORTANT
*Self-reported arm function*												
7	Randomized trials	Not serious	Serious^d^	Not serious	Not serious	None	103	96	-	SMD **0.39 higher** (0.09 lower to 0.88 higher)	⊕⊕⊕⊕ Moderate	IMPORTANT

CI: confidence interval; SMD: standardized mean difference; PICO: patients, intervention, comparison, outcome. *Explanations.* (a) We downgraded for inconsistency due to a low number of studies, small sample size, moderate heterogeneity, and variation in point estimates. (b) We downgraded for indirectness due to variation in intervention and outcome. The population and stroke onset are quite similar, but the interventions focus on either sitting or standing static balance or is lacking information of intervention with different technology app versus virtual reality. (c) We downgraded for imprecision due to a small, pooled sample size that may have affected the reported outcome. (d) We downgraded inconsistency due to a moderate heterogeneity, which we explored heterogeneity in subgroup analyses by PICO.

## Discussion

This review demonstrated that in existing studies digital home rehabilitation compared to the control group (home training/clinic-based) showed similar effects to all outcomes. This result indicated that digital home rehabilitation has the potential to replace home training or clinic-based services and was further investigated separately in subgroups for outcomes (see Supplemental Files 7–9). Still, the number of stroke survivors included in these studies were limited, which might influence the results. When comparing this overall similar efficacy with studies with controls situated in clinic-based or combined settings these results are promising. As these studies contained primarily dose-based groups except one study with even with higher training dose among controls, this result indicates that the amount of time and resources therapists interact with patients in clinics are as effective as when digitally self-managed at home with less supervision. However, when comparing the efficacy with the studies where controls trained at home with less or no supervision the results are less encouraging due to expenses related to the digital home rehabilitation programmes. Still, the results indicated a tendency of higher effect in favour of digital home rehabilitation, but with a wide CI. Most of the participants included in our review study, were middle-aged with mostly chronic ischaemic stroke partly adherent to programmes. These characteristics suggest that our results can have implications for elderly stroke participants in the moderate chronic phase.

Consistent with our findings, other systematic reviews and meta-analysis found equivalent effects between digital home rehabilitation compared to the control group on motor ability for the upper limb and balance.^27,50,51^ Also, other reviews with narrower interventions and particular use of VR compared with an alternative intervention or on upper limb function and activity showed no significant difference.^52,53^ Despite observing similar effects, one review by Schröder et al.^51^ calculated a cost-efficient effect of tele-rehabilitation and VR in balance training compared to traditional therapist-supervized care.

Our meta-analysis on static balance and lower limb function only included a few studies^32,33,48^ and similar reviews on interventions for lower limb functions are scarce.^50,54,55^ A possible reason for few studies on lower limb function might be safety precautions as there is 73% increased risk of falling after regaining gait function for stroke survivors.^54^

In our review, the meta-analysis showed inconsistent results and no effects of digital home rehabilitation and supervision in improving levels of QOL. Variation in the measurement of QOL might be one reason to the result's heterogeneity. Secondly, QOL as an outcome assessed on group level is not sensitive to change over intervention periods in between 2 to 8 weeks. To fully evaluate the effect of QOL for stroke survivors, individual-level assessments and possibly in combination with group level is more useful in clinical practice. We also highlight using valid and reliable instruments for stroke survivors and careful consideration into using generic or specific QOL instruments. It has been argued that generic measures fail to measure content validity and the important features of impairments specific for the stroke population.^31^ However, this review evaluating the psychometric properties of both generic and specific QOL instruments for stroke survivors found that EQ5 had highest and moderate quality evidence for 3/10 psychometric properties being test-retest reliability, construct validity and responsiveness. Stroke-specific QOL (SS-QOL), as one of the used QOL instruments in this review, had moderate evidence for internal consistency and conflicting evidence on construct validity.^31^

Our meta-analysis showed a small, not significant, but potential effect in improving self-reported arm function. Subgroup analysis of this outcome was explored to generate hypothesis (Supplemental File 9) and showed diversity in effect estimates for instruments measuring quality of use and quantity of arm use. It was a significant and higher moderate effect in favour of digital home rehabilitation in improving quality of arm use rather than amount of arm using the instrument Motor Activity Log-30. One possible explanation for this result might be a variation of intensity in rehabilitation among studies reporting the different items of the arm function instrument and ability to target effect. Notably, the three studies reporting on quality of arm use all featured high-intensity digital home rehabilitation aiming for repetitive range of motion using either VR or digital constraint-induced therapy. Conversely, the studies focusing on quantity of arm use displayed a wider variation in training intensity, both in the intervention and control groups. This suggests a possible, but inconsistent, relationship between meaningful intensity and challenge in digital rehabilitation and its’ effect on arm use. This is consistent with a systematic review on AR and VR in hand rehabilitation reporting a positive relationship between patients’ motivation and use of digital rehabilitation with challenge.^4^ Yet, few studies were pooled in this subgroup analysis and makes it difficult to identify independent effects. The effect estimate in this meta-analysis exceeds the threshold for minimal detectable change, yet not minimally clinically important difference for patients post-stroke.^56^ There is no other review assessing self-reported arm use. It is an important outcome in terms of activity and participation in the International Classification of Functioning Disability and Health (ICF) framework well adapted in clinical settings and research. So far, most studies have reviewed outcomes related to body function, body structure, and activity, but fewer on participation.^6^

In our review we found no significant effect in the chosen outcomes in favour of digital home rehabilitation, however within the included studies there were other outcomes not in scope of this review that proved significantly effective between groups, for instance, kinematic outcomes with significant effects in shoulder range of motion,^19^ compliance,^32^ self-efficacy,^32,40^ or in motivation.^40^ Self-reported outcomes of self-efficacy and motivation are also often lacking in systematic reviews, yet are highly relevant due to the chronic implication of stroke with individuals needing to persist with long-term rehabilitation despite small and slow progress.^55^

### Digital home rehabilitation in this review

The highlighted principles underlying effective neurorehabilitation are task-specific, goal-oriented, intensive training with implicit and explicit feedback.^57^ Among the included studies in this review, all interventions aimed for task-specific motor rehabilitation. However, the intensity of motor tasks in the rehabilitation programmes varied significantly. The training dosage in the intervention groups varied from low intensity of 15 min five days a week in three consecutive weeks in one study,^33^ to one hour five days a week for a month in another study.^46^ Some research point to that intensity while training has a larger impact on motor learning than duration of intervention.^57^ The duration of interventions was also explored in a subgroup analysis in this review (see Supplemental File 7), indicating no significant difference in effect estimate between whether interventions lasted more than four weeks or less. However, variations in the interventions appear according to motor relearning to have an impact on efficacy.^57^ In this review there was less variation in the rehabilitation programme by Allegue with fewer games^40^ in comparison to many IADL activities in studies by Adams^44^ and Cramer^42^ respectively. Two other studies explicitly also described different tasks based on the type of motion required with varying movement speed, range of motion, target size or level of cognitive demand.^20,45^ Another important principle is the ability to grade the difficulty in terms of progression,^57^ where feedback, challenge, and individualized difficulty are known features of motor learning.^58^ Mostly this was adjusted through telesupervision, yet in two studies^19,40^ level of difficulty, speed, and trajectories of arm movements were remotely and asynchronously adjusted by a therapist to obtain optimal challenge according to the participants’ improvement. In one study this kinematic visual feedback was evident for both the participant and the supervising therapist.^19^ In seven out of the eight studies on exergaming or VR there were feedback in the solutions with both explicit knowledge of results (e.g., game scores) and/or implicit feedback of knowledge of performance (e.g., movement quality).^13,33,40,42,44–46^ Explicit motivational interviewing as feedback was present in four studies.^20,40,42,47^ Half of the studies included also described possibility of monitoring performance and progression, but there were large variations in possibility of reward and automatic level adaptions. The monitoring of performance, such as speed, accuracy, and score, was mainly available for the therapist^33,40,42^ or on company servers,^13^ only few described accessibilities for the patient.^45^ Also, the reward and automatic level adaptions were mainly absent except in three studies.^13,44,45^ The implementation of these suggested principles enhances a disability adaption necessary for scalability of purpose-designed virtual environments and increases the engagement and persistence in the tasks.^6^ This intrinsic motivation can in return lead to higher adherence^55^ as well as providing learning through cognitive processing.^57^

Consistent with motor learning theory it was found in existing systematic reviews and meta-analysis that personalized VR games have more positive impact on motor function of stroke survivors compared to commercial systems due to greater emphasis on number of repetitions, feedback, and motivation.^58,59^ There is some evidence in the effect of VR to induce neural plasticity changes and functional improvements from acute and chronic phase of stroke.^57,60^ More advanced VR, combined with augmented reality (AR) imposing a computer-generated image of the user's real motions, seems to have benefits exceeding traditional VR.^6^ In this current review, AR was accessible in six out of eight included VR studies enabled by motion tracking either by Kinect camera or other 3D motion tracking. A review reported that this feedback in of AR and VR in hand rehabilitation was linked to the patients’ motivation and adherence.^4^ Looking at the results of the individual studies of VR in this review (see Table 1b) suggest a stronger effect in favour of digital home rehabilitation, except in one study^33^ lacking dose-matched training between groups. Interestingly, the study by Adams^44^ contained most of these recommended principles and gained a significant higher positive effect than the other trials in the meta-analysis for evaluating motor ability for upper limb. Similarly to this specific glove rehabilitation interacting with VR^44^ one review^4^ explained the effectiveness of haptic gloves by abilities for free hand movements in natural interactions giving the patient control over the technology. However, more studies with appropriate high intensity would need to be included to confirm this hypothesis.

All included studies in this review used a telesupervision but with only partly descriptions on content. Also, the amount of supervision varied between studies. For instance, one study only provided limited supervision,^13^ yet others supervised close to three times a week during the intervention period.^40^ There is evidence that incorporating motivation and behavioural interventions within motor rehabilitation emits clinically meaningful progression in function and ADL tasks.^20^

Another important factor to consider is whether the included studies compared post-training and retention after a gap of no training. In this review four studies did not have a follow-up testing. Among the studies with follow-up and a continued effect of the intervention suggests how well the trained movements were learned after a retention gap.

### Strengths and limitations

A strength of our review is a highly comprehensive search strategy across six databases covering grey literature and detailed interpretation of the results in the context of other evidence. This review has some limitations related to the chosen eligibility criteria and the quality of studies reviewed. First, most of the included RCTs in this review have small sample sizes which together with a relatively small amount of included RCTs might lead to underpowered results and limited statistical validation. Second, some included studies lacked description of content, dose and setting of interventions mostly related to the control groups, which makes it difficult to compare the training dose of the training programme and the interaction with therapists between intervention and control group. We chose to include stroke survivors living at home, but where some in the control group received training at home and others as outpatients at rehabilitation centres, which is the natural context of post-stroke rehabilitation. However, these two different types of control group and lack of clear descriptions in the studies lower the internal validity to some degree. We excluded studies published in languages other than English and thus some relevant studies might be missed. Due to the context of digital rehabilitation programmes delivered in the home setting we added search terms of the home to the search strategy. We are aware that this choice could lead to missing few relevant articles. To ensure all relevant studies were included we searched reference lists in all relevant existing systematic reviews and included RCT studies. Consistent with findings in this and other reviews digital home rehabilitation can entail various technologies for upper limb and lower limb aimed at improving different outcomes. The amount of heterogeneity in this review was handled by separating outcomes in different meta-analyses.

## Conclusion

This current systematic review and meta-analysis demonstrated that home-based digital motor rehabilitation and supervision lead to similar effects compared to the control group (home training/clinic-based) in improving the motor ability of upper limb, balance, QOL and self-reported arm function. These results suggest that digital home rehabilitation solutions for stroke population have the potential to replace conventional training at home or in clinic-based settings. Studies providing many technological features targeting motor learning principles seems feasible for ensuring motivation. Still, dynamic solutions that encourage and engage participants with home motor rehabilitation are warranted. Rehabilitation games and VR home solutions are better targeted and might be a feasible and promising digital approach for supporting people with post-stroke impairments in the future. Still, there is a need for RCT studies of more advanced motor features (including upper limb, lower limb, social, communication and feedback functions) with particular attention to training dose and relevant and meaningful outcome effects for the stroke survivor. Further investigation of instruments for arm function as a potential rehabilitation outcome might be relevant to assess in solutions targeting upper extremity. Combined with studies of larger sample size and more specific inclusion criteria to obtain more homogenous groups, this can potentially elucidate the direction of effect favouring digital home rehabilitation.

## Supplemental Material

sj-docx-1-dhj-10.1177_20552076241256861 - Supplemental material for Effectiveness of digital home rehabilitation and supervision for stroke survivors: A systematic review and meta-analysis Supplemental material, sj-docx-1-dhj-10.1177_20552076241256861 for Effectiveness of digital home rehabilitation and supervision for stroke survivors: A systematic review and meta-analysis by Ann Marie Hestetun-Mandrup, Zheng An Toh, Hui Xian Oh, Hong-Gu He, Anne Catrine Trægde Martinsen and Minna Pikkarainen in DIGITAL HEALTH

sj-docx-2-dhj-10.1177_20552076241256861 - Supplemental material for Effectiveness of digital home rehabilitation and supervision for stroke survivors: A systematic review and meta-analysis Supplemental material, sj-docx-2-dhj-10.1177_20552076241256861 for Effectiveness of digital home rehabilitation and supervision for stroke survivors: A systematic review and meta-analysis by Ann Marie Hestetun-Mandrup, Zheng An Toh, Hui Xian Oh, Hong-Gu He, Anne Catrine Trægde Martinsen and Minna Pikkarainen in DIGITAL HEALTH

## References

[1] Béjot Y Bailly H Durier J , et al. Epidemiology of stroke in Europe and trends for the 21st century. Presse Med 2016; 45: e391–e3e8. 10.1016/j.lpm.2016.10.003 27816343

[2] Langhorne PF Coupar FB Pollock AP . Motor recovery after stroke: a systematic review. Lancet Neurol 2009; 8: 741–754. 19608100 10.1016/S1474-4422(09)70150-4

[3] Feigin VL Norrving B Mensah GA . Global burden of stroke. Circ Res 2017; 120: 439–448. 28154096 10.1161/CIRCRESAHA.116.308413

[4] Pereira MF Prahm C Kolbenschlag J , et al. Application of AR and VR in hand rehabilitation: a systematic review. J Biomed Inform 2020; 111: 103584. 33011296 10.1016/j.jbi.2020.103584

[5] Meyer S Verheyden G Brinkmann N , et al. Functional and motor outcome 5 years after stroke is equivalent to outcome at 2 months: follow-up of the collaborative evaluation of rehabilitation in stroke across Europe. Stroke 2015; 46: 1613–1619. 25953370 10.1161/STROKEAHA.115.009421

[6] Amorim P Santos BS Dias P , et al. Serious games for stroke telerehabilitation of upper limb-a review for future research. Int J Telerehabil 2020; 12: 65–76. 33520096 10.5195/ijt.2020.6326 PMC7757643

[7] Øra HP Kirmess M Brady MC , et al. The effect of augmented speech-language therapy delivered by telerehabilitation on poststroke aphasia—a pilot randomized controlled trial. Clin Rehabil 2020; 34: 369–381. 31903800 10.1177/0269215519896616

[8] Zhou X Du M Zhou L . Use of mobile applications in post-stroke rehabilitation: a systematic review. Top Stroke Rehabil 2018; 25: 1–11. 30209991 10.1080/10749357.2018.1482446

[9] Pugliese M Ramsay T Shamloul R , et al. Recovernow: a mobile tablet-based therapy platform for early stroke rehabilitation. PLOS ONE 2019; 14: e0210725. 10.1371/journal.pone.0210725 PMC6347149 30682076

[10] Schwamm LH Chumbler N Brown E , et al. Recommendations for the implementation of telehealth in cardiovascular and stroke care: a policy statement from the American heart association. Circulation 2017; 135: e24–e44. 10.1161/CIR.0000000000000475 27998940

[11] Moral-Munoz JA Zhang W Cobo MJ , et al. Smartphone-based systems for physical rehabilitation applications: a systematic review. Assist Technol 2021; 33: 223–236. 31112461 10.1080/10400435.2019.1611676

[12] Burns SP Terblanche M Perea J , et al. Mhealth intervention applications for adults living with the effects of stroke: a scoping review. Arch Rehabil Res Clin Transl 2021; 3: 100095. 33778470 10.1016/j.arrct.2020.100095 PMC7984984

[13] Hernandez A Bubyr L Archambault PS , et al. Virtual reality-based rehabilitation as a feasible and engaging tool for the management of chronic poststroke upper-extremity function recovery: randomized controlled trial. JMIR Serious Games 2022; 10: e37506. 10.2196/37506 PMC9555337 36166289

[14] Perle JG Zheng W . A primer for understanding and utilizing telesupervision with healthcare trainees. J Technol Behav Sci 2023: 1–7. 10.1007/s41347-023-00322-5 PMC10196304 37362064

[15] Conroy SS Harcum S Keldsen L , et al. Novel use of existing technology: a preliminary study of patient portal use for telerehabilitation. J Telemed Telecare 2022; 28: 380–388. 32869689 10.1177/1357633X20950172

[16] Jones M Collier G Reinkensmeyer DJ , et al. Big Data Analytics and Sensor-Enhanced Activity Management to Improve Effectiveness and Efficiency of Outpatient Medical Rehabilitation. Int J Environ Res Public Health 2020; 17: 748. 31991582 10.3390/ijerph17030748 PMC7037379

[17] Farrahi V Niemelä M Kangas M , et al. Calibration and validation of accelerometer-based activity monitors: a systematic review of machine-learning approaches. Gait Posture 2019; 68: 285–299. 30579037 10.1016/j.gaitpost.2018.12.003

[18] Man DW Tam SF Hui-Chan CW . Learning to live independently with expert systems in memory rehabilitation. NeuroRehabilitation 2003; 18: 21–29. 12719618

[19] Toh FM Lam WW Gonzalez PC , et al. ‘Smart reminder': a feasibility pilot study on the effects of a wearable device treatment on the hemiplegic upper limb in persons with stroke. J Telemed Telecare 2024: 1357633X231222297. 10.1177/1357633X231222297 38196179

[20] Gauthier LV Nichols-Larsen DS Uswatte G , et al. Video game rehabilitation for outpatient stroke (VIGoROUS): a multi-site randomized controlled trial of in-home, self-managed, upper-extremity therapy. eClinicalMedicine 2022; 43: 101239. 34977516 10.1016/j.eclinm.2021.101239 PMC8688168

[21] Tornivuori A Tuominen O Salanterä S , et al. A systematic review on randomized controlled trials: coaching elements of digital services to support chronically ill adolescents during transition of care. J Adv Nurs 2020; 76: 1293–1306. 32030792 10.1111/jan.14323

[22] Saragih ID Tarihoran D Batubara SO , et al. Effects of telehealth interventions on performing activities of daily living and maintaining balance in stroke survivors: a systematic review and meta-analysis of randomised controlled studies. J Clin Nurs 2021; 31: 2678–2690. 34873756 10.1111/jocn.16142

[23] Hwang NK Park JS Chang MY . Telehealth Interventions to Support Self-Management in Stroke Survivors: a Systematic Review. Healthcare (Basel, Switzerland) 2021; 9: 472. 33921183 10.3390/healthcare9040472 PMC8071480

[24] Oh HX De Silva DA Toh ZA , et al. The effectiveness of self-management interventions with action-taking components in improving health-related outcomes for adult stroke survivors: a systematic review and meta-analysis. Disabil Rehabil 2021; 44: 1–16. 10.1080/09638288.2021.2001057 34757862

[25] Sarfo FS Ulasavets U Opare-Sem OK , et al. Tele-Rehabilitation after stroke: an updated systematic review of the literature. Journal of Stroke and Cerebrovascular Diseases : The Official Journal of National Stroke Association 2018; 27: 2306–2318. 29880211 10.1016/j.jstrokecerebrovasdis.2018.05.013 PMC6087671

[26] Freund M Carey M Dilworth S , et al. Effectiveness of information and communications technology interventions for stroke survivors and their support people: a systematic review. Disabil Rehabil 2021; 44: 1–16. 10.1080/09638288.2021.1913245 33905279

[27] Laver KE Adey-Wakeling Z Crotty M , et al. Telerehabilitation services for stroke. Cochrane Database Syst Rev 2020; 1: CD010255. 10.1002/14651858.CD010255.pub3 PMC6992923 32002991

[28] Chen J Jin W Zhang XX , et al. Telerehabilitation approaches for stroke patients: systematic review and meta-analysis of randomized controlled trials. Journal of Stroke and Cerebrovascular Diseases: The Official Journal of National Stroke Association 2015; 24: 2660–2668. 26483155 10.1016/j.jstrokecerebrovasdis.2015.09.014

[29] Da-Silva RH Moore SA Price CI . Self-directed therapy programmes for arm rehabilitation after stroke: a systematic review. Clin Rehabil 2018; 32: 1022–1036. 29756513 10.1177/0269215518775170

[30] Heiberg G Pedersen SG Friborg O , et al. Can the health related quality of life measure QOLIBRI-overall scale (OS) be of use after stroke? A validation study. BMC Neurol 2018; 18: 1–10. 30021558 10.1186/s12883-018-1101-9 PMC6052666

[31] Cameron LJ Wales K Casey A , et al. Self-reported quality of life following stroke: a systematic review of instruments with a focus on their psychometric properties. Qual Life Res 2021; 31: 1–14. 34247327 10.1007/s11136-021-02944-9

[32] Chen S Huang J Yao L , et al. Internet + continuing nursing (ICN) program promotes motor function rehabilitation of patients with ischemic stroke. Neurologist 2022; 27: 56–60. 10.1097/NRL.0000000000000364 34842574

[33] Krpic A Savanovic A Cikajlo I . Telerehabilitation: remote multimedia-supported assistance and mobile monitoring of balance training outcomes can facilitate the clinical staff’s effort. Int J Rehabil Res 2013; 36: 162–171. 23337324 10.1097/MRR.0b013e32835dd63b

[34] Higgins JPTTJ Chandler J Cumpston M , et al. Cochrane handbook for systematic reviews of interventions version 2021; 6: p. 22021.

[35] Liberati A Altman DG Tetzlaff J , et al. The PRISMA statement for reporting systematic reviews and meta-analyses of studies that evaluate healthcare interventions: explanation and elaboration. Br Med J 2009; 339: b2700. 10.1136/bmj.b2700 PMC2714672 19622552

[36] Bernhardt J Hayward KS Kwakkel G , et al. Agreed definitions and a shared vision for new standards in stroke recovery research: the stroke recovery and rehabilitation roundtable taskforce. Neurorehabil Neural Repair 2017; 31: 793–799. 28934920 10.1177/1545968317732668

[37] Hozo SP Djulbegovic B Hozo I . Estimating the mean and variance from the median, range, and the size of a sample. BMC Med Res Methodol 2005; 5: 1–10. 15840177 10.1186/1471-2288-5-13 PMC1097734

[38] Sterne JA Sutton AJ Ioannidis JP , et al. Recommendations for examining and interpreting funnel plot asymmetry in meta-analyses of randomised controlled trials. Br Med J 2011; 343: d4002. doi: 10.1136/bmj.d4002 21784880

[39] Guyatt GH Oxman AD Kunz R , et al. What is “quality of evidence” and why is it important to clinicians? Br Med J 2008; 336: 995–998. 18456631 10.1136/bmj.39490.551019.BE PMC2364804

[40] Allegue DR Higgins J Sweet SN , et al. Rehabilitation of Upper Extremity by Telerehabilitation Combined with Exergames in Survivors of Chronic Stroke: preliminary Findings from a Feasibility Clinical Trial. JMIR Rehabil Assist Technol 2022; 9: e33745. 10.2196/33745 PMC9260524 35731560

[41] Babic A Tokalic R . Amílcar Silva Cunha J ; ., et al. Assessments of attrition bias in cochrane systematic reviews are highly inconsistent and thus hindering trial comparability. BMC Med Res Methodol 2019; 19: 76. 30953448 10.1186/s12874-019-0717-9 PMC6451283

[42] Cramer SC Dodakian L Le V , et al. Efficacy of home-based telerehabilitation vs in-clinic therapy for adults after stroke: a randomized clinical trial. JAMA Neurol 2019; 76: 1079–1087. 31233135 10.1001/jamaneurol.2019.1604 PMC6593624

[43] Chen SQ Huang JH Yao L , et al. Internet plus continuing nursing (ICN) program promotes motor function rehabilitation of patients with ischemic stroke. Neurologist 2022; 27: 56–60. 10.1097/NRL.0000000000000364 34842574

[44] Adams RJ Ellington AL Kuccera KA , et al. Telehealth-Guided virtual reality for recovery of upper extremity function following stroke. OTJR-Occup. Part. Heal. 2023; 43: 15394492231158375. 10.1177/15394492231158375 PMC10499117 36960762

[45] Kizony R Weiss PL Feldman Y et al (eds) Evaluation of a Tele-Health System for upper extremity stroke rehabilitation 2013. 2013 International Conference on Virtual Rehabilitation (ICVR), Philadelphia, PA, USA: IEEE Computer Society, 2013.

[46] Piron L Turolla A Agostini M , et al. Exercises for paretic upper limb after stroke: a combined virtual-reality and telemedicine approach. J Rehabil Med 2009; 41: 1016–1020. 19841835 10.2340/16501977-0459

[47] Uswatte G Taub E Lum P , et al. Tele-rehabilitation of upper-extremity hemiparesis after stroke: proof-of-concept randomized controlled trial of in-home constraint-induced movement therapy. Restor Neurol Neurosci 2021; 39: 303–318. 34459426 10.3233/RNN-201100

[48] Salgueiro C Urrútia G Cabanas-Valdés R . Telerehabilitation for balance rehabilitation in the subacute stage of stroke: a pilot controlled trial. NeuroRehabilitation 2022; 51: 91–99. 35311721 10.3233/NRE-210332

[49] Chen SQ Huang JH Yao L , et al. Internet plus continuing nursing (ICN) program promotes motor function rehabilitation of patients with ischemic stroke. Neurologist 2022; 27: 56–60. 10.1097/NRL.0000000000000364 34842574

[50] Rintala A Hakala S Paltamaa J , et al. Effectiveness of technology-based distance physical rehabilitation interventions on physical activity and walking in multiple sclerosis: a systematic review and meta-analysis of randomized controlled trials. Disabil Rehabil 2018; 40: 373–387. 27973919 10.1080/09638288.2016.1260649

[51] Schröder J Van Criekinge T Embrechts E , et al. Combining the benefits of tele-rehabilitation and virtual reality-based balance training: a systematic review on feasibility and effectiveness. Disability and Rehabilitation: Assistive Technology 2019; 14: 2–11. 30318952 10.1080/17483107.2018.1503738

[52] Laver KE Lange B George S , et al. Virtual reality for stroke rehabilitation. Cochrane Database Syst Rev 2017; 11: CD008349. 10.1002/14651858.CD008349.pub4 PMC6485957 29156493

[53] Hao J Pu Y Chen Z , et al. Effects of virtual reality-based telerehabilitation for stroke patients: a systematic review and meta-analysis of randomized controlled trials. J Stroke Cerebrovasc Dis 2023; 32: 106960. 36586244 10.1016/j.jstrokecerebrovasdis.2022.106960

[54] Park S Tang A Pollock C , et al. Telerehabilitation for lower extremity recovery poststroke: a systematic review and meta-analysis protocol. BMJ Open 2022; 12: e055527. 10.1136/bmjopen-2021-055527 PMC8915270 35264359

[55] Chan KGF Jiang Y Choo WT , et al. Effects of exergaming on functional outcomes in people with chronic stroke: a systematic review and meta-analysis. J Adv Nurs 2022; 78: 929–946. 34877698 10.1111/jan.15125

[56] Simpson LA Eng JJ . Functional recovery following stroke: capturing changes in upper-extremity function. Neurorehabil Neural Repair 2013; 27: 240–250. 23077144 10.1177/1545968312461719 PMC4486379

[57] Maier M Ballester BR Verschure P . Principles of Neurorehabilitation After Stroke Based on Motor Learning and Brain Plasticity Mechanisms. Front Syst Neurosci 2019; 13: 00074. 10.3389/fnsys.2019.00074 PMC6928101 31920570

[58] Aminov A Rogers JM Middleton S , et al. What do randomized controlled trials say about virtual rehabilitation in stroke? A systematic literature review and meta-analysis of upper-limb and cognitive outcomes. J Neuroeng Rehabil 2018; 15: 29. 29587853 10.1186/s12984-018-0370-2 PMC5870176

[59] Doumas I Everard G Dehem S , et al. Serious games for upper limb rehabilitation after stroke: a meta-analysis. J Neuroeng Rehabil 2021; 18: 100. 34130713 10.1186/s12984-021-00889-1 PMC8204490

[60] Hao J Xie H Harp K , et al. Effects of virtual reality intervention on neural plasticity in stroke rehabilitation: a systematic review. Arch Phys Med Rehabil 2022; 103: 523–541. 34352269 10.1016/j.apmr.2021.06.024

