# Supplementary material for: Effectiveness of digital home rehabilitation and supervision for stroke survivors: A systematic review and meta-analysis
Source: Digit Health. 2024 Jun 3;10:20552076241256861. doi: 10.1177/20552076241256861 (PMC11146002; doi:10.1177/20552076241256861)

**SUPPLEMENTARY MATERIALS**

**Table of contents**

[SUPPLEMENTARY FILE 1: Existing and ongoing systematic reviews and gaps in these reviews 2](#_Toc161999668)

[SUPPLEMENTAL FILE 2: Full search strategies 22](#_Toc161999669)

[SUPPLEMENTAL FILE 3: Data extraction form and risk of bias tool 46](#_Toc161999670)

[Risk of Bias assessment 53](#_Toc161999671)

[SUPPLEMENTARY FILE 4: Included and excluded studies 54](#_Toc161999672)

[SUPPLEMENTARY FILE 5: Categorization of studies 64](#_Toc161999673)

[SUPPLEMENTARY FILE 6: Risk of bias 1 65](#_Toc161999674)

[SUPPLEMENTARY FILE 7: Subgroup analysis motor ability of upper limb 67](#_Toc161999675)

[SUPPLEMENTARY FILE 8: Subgroup analysis stroke-related quality of life 67](#_Toc161999676)

[SUPPLEMENTARY FILE 9: Subgroup analysis self-reported arm function A. Comparator B. Instrument use C. Length of intervention time 68](#_Toc161999677)

# **SUPPLEMENTARY FILE 1: Existing and ongoing systematic reviews and gaps in these reviews**

| Title | Aim | PICO | Method/results | Database searched | Remarks | Gaps in the review | Differences from my SR |
| --- | --- | --- | --- | --- | --- | --- | --- |
| Effectiveness of information and communication technology interventions for stroke survivors and their support people: a systematic review (2021)  Freund et al. | To examine the effectiveness of self-directed, off-the-shelf information and communications technology (ICT)-based interventions in improving the quality of life, physical and psychosocial outcomes of community-dwelling stroke survivors and their support persons | P: community-dwelling stroke survivors over 18 and stroke + support people over 18  I: self-directed ICT in home – tablet, desktop, smart phone, text messaging, apps, gaming consoles  C:  O: all outcomes deemed eligible such as stroke outcomes (speech, hemiparesis, cognition), physical outcomes (e.g., BT), behavioural outcomes (e.g., medication adherence, physical activity), psychosocial outcomes (e.g., depression, QOL) and health service | 8/9 reported positive outcome of the computer programs in improving stroke outcomes  5/6 of the studies investigating mobile phone-interventions reported at least one positive outcome  Few studies reported on intervention acceptability or adherence. 15 studies reported significant effect for at least one outcome on stroke specific outcomes, physical outcomes, behavioural outcomes, and health service use  Different telehealth interventions:  -linguistic + phonetic  -speech + language  -cost-eff  -cognitive rehab  Memory training  Hand + finger function  -cognition  -neuropsychological  -education programs  -medication reminders  BT+ text  -coaching system | Medline, Embase, CINAHL + Cochrane | Also includes ICT in community settings | Limited databases searched  Heterogenous outcome measures  Narrative/integrative review | My SR will include more databases in search  My SR will not focus on psychosocial or speech and language interventions (be a little less heterogenous in included outcomes)  My SR will include a transparent quality assessment and hopefully a meta-analysis |
| Effects of telehealth interventions on performing activities of daily living and maintaining balance in stroke survivors: A systematic review and meta-analysis of randomised controlled studies (2021)  Saragih et al. | Identify the effects of telehealth interventions on ability to perform ADL and maintain balance for stroke | P: stroke survivors (1367)  I: telehealth interventions (videoconference, text message, telephone calls)  C: routine or usual care  O: ADL:  -Modified Barthel Index  -Barthel Index  -Late Life Function and Disability  Balance:  -Berg Balance Scale | SR and meta-analysis of 14 RCTs  Used Cochrane risk of bias tool, 3/7 considered a high risk. In total: low risk/variance of bias  Used mean, SD and standard mean diff (SMD) with 95 % CI  Telehealth interventions: physical exercise + promotion of healthy behaviours 1-12 months  Follow-up 3,6, 12 months  Telehealth signs effective in improving ADL (101 stroke) (pooled SMD 0,45 (95% CI:0,12-0,78 p:0,01), not balance (90 stroke) (pooled 0,03 (95% CI:0,38-0,45 p:0,87) | 7 databases:  -Academic Search Complete  -CINAHL  -Embase  -Medline  -PubMed  -Ovid  -WOS | Intervention settings: community (5), home (2), long-term care (1), hospital (4) rehab centre (1), national stroke association (1)  Interventions providers: nurses (2), therapists (5), physicians (5), researcher (1)  1-12 mounts intervention time  Intervention technology:  -standard rehab program + tele rehab (sensors, hardware and recording exercises)  -Physical and occupational therapy programs + electrical stimulation  -home visits+messaging+  telephone calls  -apps: Fitlab with monitor walking  -video-conference pool exercise  -positive airway pressure  -3D animation exercise video  -telemonitoring, telephone calls + remote interventions  -DVD of standard rehab  -Bluetooth blood pressure device + smartphone with app  -video conference including CT scan and exam.  -education videos, online chat, resource room  -Bluetooth sphygmomanometer BP and wearable bracelet | High heterogeneity/variation among population (stroke survivors in community, home, long-term care, rehab centre, national stroke association)  Does not specify follow up from reference lists, grey literature or unpublished as well as published studies (uncertainty of all relevant studies were included)  Does not search in Scopus | This SR focused on various intervention settings. However, my SR will focus on stroke telerehabilitation follow-up at home (not rehab settings) and more focus to long-term rehab intervention time  This SR have multiple telehealth interventions. My SR will exclude intervention only focusing on video-conf, text message, telephone calls without a software remote care |
| Telerehabilitation services for stroke (2020)  Laver et al. (Cochrane Review) | Does the use of tele rehab lead to improved ADL among stroke survivors compared to in-person rehab or no care? | P: stroke survivors  I (see remarks)  C: 1) in-patient care 2) no care or usual care  O:  ADL (ICF)  FIM, Barthel, Lawton Instrumental ADL, Frenchay Activity index, Nottingham Extended ADL  Self-care, mobility, balance, QOL, depression, upper limb function, cognitive function, or functional communication  Report also on cost-effectiveness, feasibility, user satisfaction  Does not conclude on self-care, mobility, satisfaction, and domestic life and cognitive function  Four studies evaluated cost-effectiveness, but data on service utilisation or costs | GRADE to assess quality of evidence  22 RCTs (1937 stroke) 14 metanalysis  Moderate-quality evidence that there was no difference in 2 studies with 661 strokes on ADL between people who received a post-hospital discharge telerehabilitation intervention and those who received usual care amongst stroke survivors (SMD 0,00, 95 % CI -0,15-0,15)  Low-moderate quality of evidence that there was no difference in 2. outcomes of balance outcomes (SMD 0,08,95 % CI -0,30-0,46), depression, QOL (SMD 0,03 95 % CI. -0,14-0,20), upper limb function, mobility. | 12 databases:  -Cochrane Stroke Group Trials Register  -Cochrane Lib.  -Medline  -Embase  -8 extra  Ongoing trials + grey lit + scanned ref lists | Included RCTs that compared two different methods of tele rehab without control  Rehab programs with combinations of tele rehab (>) and in-person rehab  22 ongoing studies (Gap?)  Intervention approach:  -post-hospital support programs  -upper limb training  -lower limb  -mobility retraining  -communication therapy  Varied population and all stages post-stroke: after discharge from hospital, subacute phase, chronic  Also included subarachnoid haemorrhage  Comparator: 1) in-patient care 2) no care or usual care. Sub-grouped ADL vs usual care and ADL vs in-patient care. What’s the difference?  All interventions in their homes, except one with homecare  Interventions:  -goal settings, education on secondary prevention, family therapy, improve function (also customised computer-based training programmes), balance using customised tele rehab systems and communication, exercises + el. stimulations, combination of occupational+physiotherapy rehabilitation + speech and language therapy with aphasia  Technologies:  Telephone, videoconference hardware and software, desktop videophones, combinations (calls+message+videoconference+monitoring, email+chat  4 studies combination of ICT + home visits | Interventions and comparators varied/heterogeneity  Few powered studies and studies with risk of bias | This SR also included RCTs that compared 2 different tele rehab without control. My SR will include RCTs with a control. This SR had a high heterogeny of interventions and technology. My SR will have a more constant intervention and technology.  My SR will preferably aim for purpose-designed tele rehab systems |
| Telerehabilitation Approaches for Stroke Patients: Systematic Review and Meta-analysis of Randomized Controlled Trials (2015)  Chen et al. | Does tele rehab lead to improvement of ADL for stroke patients?  Long and short term tele rehab with/+- without VR-training was not superior/equal to conventional rehab in improving ADL and motor function for stroke | P: stroke  I: varied, not described  O: Barthel Index  Bergs balance and FIM  Fugl-Meyer  Wolf-Motor-Function, TUG, Nine-Hole-Peg, ARAT, walking speed, Ashworth, Mini Mental State Exam, QOL, SF, satisfaction, caregiver stress, cost-eff., adverse events | SR of 11 RCTs and meta-analysis of 7 pooled RCTs on tele rehab approaches (VR or strengthened training via telephone or web)  Sub-group short/longer 6 weeks  Hypothesis failed equal effect tele rehab (both groups improved effects). With conventional care on Barthel index (SMD-0,05 95 % IC: -0,24-0,13, Berg’s balance scale (SMD -0,05 95 % CI:-0,7-0,37), functional independence measure as 1.outcome. Meta-analysis pooled data from 4 trials which used BI as outcome measure of ADL, and 2 trials Fugl-Meyer Upper extremity scale, 2 trials of Bergs balance test | 7 databases:  Searched for referenced 2000-2015  -Cochrane Library  -AMED  -Medline  -Embase  -CINAHL  -PsycINFO  -WOS | Wanted to make an updated SR from the Cochrane one in 2013.  One study Llorens et al reported data on cost-eff. – VR telerehabilitation lower cost than conventional | Requires an update of evidence since 2015  Does not search in Scopus  High heterogeneity in 4 trails assessing BI to measure ADL  Variety among telerehabilitation approaches – lack of detailed description of intervention and technology  Studies used FIM to measure ADL were heterogenous + did no pool data and no analysis | This SR focused on telerehabilitation interventions with/without VR. My SR will only include VR if it is in combination with software remote rehab. |
| Telehealth Interventions to support self-management in stroke survivors: a systematic review (2021)  Na-Kyoung Hwang et al. | Identifying tele-health self-management support focus, delivery, and effects in stroke survivors | P: clinical diagnose of stroke  I:SM support – telephone calls, web- or mobile-app-based interventions  C: not all studies  O: (1) SM behaviour, goal attainment, ADL, participation, and medical adherence. 2) clinical outcome, disability, function, fatigue 3) self-efficacy 4) QOL 5) acceptance, satisfaction | Identifies RCTs, quasi-experimental, re-post study.  10 broad tele-health self-management focus, components, delivery type+effect also without control group)  Messaging, next video-conf. most used TH delivery type (also use in-home messaging service or platform)  Education on recovery, prevention, next relief of depressive symptoms and lifestyle support (8/10) most used component | Searched from 2005-2020  5 databases:  -Medline  -Embase  -CINAHL  -PsycINFO  -WOS | Post-stroke depression  Obesity management  Participation  Functional mobility  ADL  Assess quality of RCTs by Pedro scale. ¾ studies high quality  Risk of Bias assessment tool for non-randomised studies (RoBANS)  Does give a good description different studies TH delivery | Integrative/narrative review and therefore limit to confirm consistent improvement in SM outcomes (no meta-analysis)  Excludes specific interventions, only includes broad interventions  Uses vote-counting to show how many positive results. Limited presented evidence to suggest effects and draw conclusions | This SR is focused on broad interventions and excludes studies with more consistent interventions. My SR will have more constant intervention and technology.  This SR have low methodological quality.  My SR will have higher methodological quality. |
| Self-directed therapy programmes  for arm rehabilitation after stroke:  a systematic review (2018)  Ruth H Da-Silva et al. | To investigate the effectiveness of self-directed arm interventions in adult stroke survivors. | P: stroke patients over 18 years + populations with mixed impairment  aetiology was included if at least 50% of participants  had experienced a stroke.  I: self-directed arm interventions. Self-directed if more than 50% of  the overall intended duration of therapy practice,  was independently initiated and carried out by the  participant outside of direct contact sessions  C: not all studies  O: many outcomes-function/impairment, independence/self-care | A systematic review of 40 studies (1172 participants)  for inclusion. 19 randomized controlled trials (RCTs) and 21 before–after studies) on effectiveness of self-directed (home) arm interventions in adult stroke survivors with/without technology  16 RCTs in metanalysis  Constraint-induced movement therapy and therapy programmes without technology improved independence in ADL  No clear effect other interventions interactive gaming, robotic and dynamic orthotic devices, mirror therapy, telerehabilitation *and* wearable devices  Sensitivity analysis demonstrated arm function benefit for patients  >12 months poststroke (*n* = 145; SMD 0.52, 95% CI 0.21–0.82) but not at 0–3, 3–6 or 6–12 months. | 5 databases:  Medline, EMBASE, CINAHL, SCOPUS and IEEE Xplore | Only one telerehabilitation study met the criteria  for meta-analysis;27 however, as telerehabilitation  was not the intervention being tested but rather  a means of delivering the therapy remotely, this  study has been included in the robotic devices’ subgroup  of the analysis. | Not within the telerehabilitation overall field  Not a constant intervention – lots of mixes of dosage and overlap of type interventions  Low methodological quality, only one person did the screening + data extraction  Overall heterogeneity  was substantial in terms of the types of  interventions studied, reporting of the amount of  self-directed practice and the time poststroke of  participants potentially limiting findings | Different intervention, not merely in telerehabilitation.  This SR will focus on digital home technology and more than just arm interventions |
| mHealth Intervention Applications for  Adults Living With the Effects of Stroke: A  Scoping Review (2021)  Burns et al. | To conduct a scoping review of mobile health (mHealth) application  (app) interventions to support needs of adults living with the effects of stroke reported in  the literature | Does not explicit its PICOs  Only search terms included: stroke, cerebral vascular  accident, recovery, rehabilitation, smartphone, mobile  applications, mHealth, telerehabilitation, and  telemedicine | 49 were included for data extraction  38 mHealth apps for use by individuals after stroke. The review revealed (1) the studies were generally describing development and refinement or pilot/feasibility trials (61%) 10 articles (20 %) RCTs.  Broad focus on upper ex function (31 %), lower ex (5 %), general exercise, physical activity, and mobility (23,7 %), trunk control (5 %), medical management and secondary prevention (26 %), language (20 %), cognitive skills (8 %), general disability and activities of daily living (5 %), home safety (2,6%). | 3 databases:  -PubMed,  -CINAHL  -Scopus |  | Limited databases searched  Scoping review  No/Limited evidence to suggest benefits of mHealth applications  No comparator or outcomes | My SR will search more databases  My SR will have a quality assessment of included articles and evaluate effect (by narrative or meta-analysis) |
| Activity monitors for increasing physical activity in adult stroke survivors (Cochrane Review 2019)  Lynch et al. | To summarise the available evidence regarding the effectiveness of commercially available, wearable activity monitors and smartphone applications for increasing physical activity levels in people with stroke | P: stroke in hospital or living in the community  I: any wearable or portable electronic device that provided feedback (in either real time, or on a regular basis, e.g., daily, or weekly) on physical activity.  C: no intervention, another type of intervention, or other activity monitor.  O: 1) steps per day and time in moderate-to-vigorous intensity activity 2) sedentary time, time spent in light intensity physical activity, walking duration, fatigue, mood, quality of life, community participation and adverse events | 4 RCTs in 11 reports  Low quality evidence of no clear effect for the use of wearable activity monitors and smartphone applications in 4 RCTs compared with other interventions on step counts per day in a community or inpatient setting for stroke patients  One small study showed sign effect of activity monitors on time spent in moderate-intensity physical activity but no significant effect on time spent in vigorous-intensity physical activity in inpatient settings | Cochrane Stroke Group Trials Register, CENTRAL, MEDLINE, Embase, CINAHL, SPORT Discus + clinical trial registers | Activity monitors used were triaxial accelerometers (Gulf Coast Data Concepts) worn with one sensor on each ankle (Dorsch 2015), one sensor on each limb (Mansfield 2015), Fitbit One (Kanai 2018), and the Step Watch activity monitor (Danks 2016). | Few RCTs  Only able to pool data on one outcome measure from two studies | Different aim. This SR more focused on monitoring than the telehealth solution. My SR will only include monitoring combined with remote software rehab solutions.  This SR focus on in-patient settings. My SR will exclude in-patient settings.  This SR focus on mostly clinical outcomes, and few self-reported outcomes (e.g., on self-management) My SR will focus on both clinical and self-reported outcomes. |
| Tele-Rehabilitation after Stroke: An Updated Systematic  Review of the Literature (2018)  Sarfo et al. | Efficacy of tele-rehabilitation interventions for recovery from motor,  higher cortical dysfunction, and poststroke depression among stroke survivors. | P: stroke  I: telemedicine, telecommunication media,  and intervention programs including phone,  videoconferencing, tele-rehabilitation system, robot assisted  rehabilitation, and virtual and augmented  reality therapy.  C: conventional or no  O: Barthel Index scale, Berg Balance Scale, and Functional Independence Measure scale. | A systematic review of 22 studies (RCTs, pilot trials, or feasibility trials) on tele-rehabilitation for motor and higher cortical deficits as well as poststroke depression appear to be as effective as in-person therapies, if not better.  18 studies effect of tele-rehabilitation on motor function, 2 were on depression and higher cortical dysfunction respectively (aphasia and hemi-neglect. | 2 databases:  PubMed and Cochrane library | States that tele-rehabilitation for motor and higher cortical deficits  as well as poststroke depression appear to be as effective  as in-person therapies, if not better. | Limited databases searched  Positive result, but does not show any statistics from the included studies, and includes feasibility, pilot RCTs | This SR focus on combination of telerehabilitation technology. My SR will focus on software remote rehab.  My SR will include only RCTs and will be able to draw a more evidence-based conclusion. |
| The effectiveness of self-management interventions with action-taking components in improving health-related outcomes for adult stroke survivors: a systematic review (2021)  Hui Xian Oh et al. | Effectiveness of self-management interventions with action-taking components for community-dwelling adult stroke survivors’ self-efficacy, HRQoL, BADL, IADL and depression compared to usual care by institutions, placebos, or inactive care | P: stroke survivors  I: self-management interventions with action-taking components  C: usual care by institutions, placebos, or inactive care  O: self-efficacy, HRQoL, BADL, IADL and depression | 17 studies included (RCTs, pilot RCTs)  7 in meta-analysis  Statistically sign effect I self-management and BADL with moderate heterogeneity | 7 databases | Coaching or reinforcing on effective goal-setting skills, facilitating the action-planning process  Excluded studies with stroke specific symptoms or disability (depression only, aphasia-only), disability management (upper limb or gait training or blood pressure management) | The included studies had high risk of bias  Unclear if this is within the scope of telerehabilitation and remote care. Seems like it is combinations | Unclear if this is within the scope of telerehabilitation and remote care. Seems like it is combination. My SR will be on remote telerehabilitation context without combination of this and home visits.  This SR focus on self-management and not clinical function outcomes. My SR will focus on outcomes of disability management, and include studies with single interventions if it fulfils the rest of the inclusion crit. |
| Ongoing: Clinical impact of mobile applications for stroke rehabilitation: a systematic review  Stephen G. Szeto et al. | To evaluate the clinical impact of mobile applications on the rehabilitation of adults with stroke | P: stroke survivors  I: mobile applications (phone, tablet, PC) on all operating systems (iOS, Android, Windows) and features applicable to mobile applications (web-based applications, message reminders e.g., SMS texts, video display)  C:  standard of care or no comparisons  O: 1. ADLs (e.g., FIM, modified Barthel index, modified Rankin Scale, Chedoke arm and hand activity inventory, activity measure-post acute care short form, iADL scale, or similar) 2. Motor function including gait (e.g., muscle strength, ROM, action research arm test, Fugl-Meyer Motor Assessment, functional dysphagia scale, Wolf motor function test, finger dexterity, or similar) 3. Adherence (e.g., medication via vascular RF control, exercise, or similar) 4. Quality of Life (e.g., EuroQol instrument EQ-5D-5L, Stroke specific QoL scale, visual analogue scale, or similar |  |  | Excludes app designed to control robotics devices, functional electrical stimulation, virtual reality headset, telerehabilitation, brain-computer interfaces |  | unsure |
| Does Physiotherapy via telerehabilitation improve physical outcomes for patients’ post-stroke?  Melissa Prause et al. |  | P: post-stroke patients in community  I: Physiotherapy (i.e., assessment, treatment, management, review) via telerehabilitation (e.g.: video conferencing through computer/iPad/smartphone  C: Face to face / traditional gym-based Physiotherapy  O: Mobility status, strength, balance, endurance, gait quality, coordination |  |  | No restriction of studies (not just RCTs) |  |  |
| Evidence of effects of telerehabilitation on function compared to usual model of care in the management of stroke patients: a systematic review  Appleby et al. | What effect does telerehabilitation have on function, independence, activities of daily living and other outcomes, when compared to conventional model of care, in the management of stroke patients? | P: adult stroke  I: Rehabilitation with health professional/s delivered and/or conducted via videoconferencing technology.  Also, commonly referred to as Telerehabilitation, E-rehabilitation, Mhealth or telemedicine.  C: Usual care; face to face rehabilitation, conventional rehabilitation  O: Function and activities of daily living |  |  | Exclusion: Virtual reality used for rehabilitation without therapist input, video games used for rehabilitation without therapist input, therapy delivered only via telephone consultations without video conferencing. |  |  |
| (completed in Chinese) Home-based telerehabilitation for stroke survivors: a systematic review  Xiaoyan Zhang et al. | What effect does home-based telerehabilitation have on function, the activities of daily life, independence, balance, quality of life and other outcomes, when compared to traditional models of care, in the management of stroke survivors? | P: adult stroke  I: Inclusion: home-based rehabilitation with health professional/s delivered and/or conducted via telerehabilitation, e-Rehabilitation, mHealth or telemedicine systems (computers, robotic assistance techniques, virtual reality and video conferencing)  C: usual care; outpatient rehabilitation; community rehabilitation; conventional rehabilitation  O: Daily life ability, motor function, balance function, caregiver stress, patient quality of life, incidence of adverse reactions |  |  | Exclusion: Virtual reality used for rehabilitation without therapist input, video games used for rehabilitation without therapist input, therapy delivered only via telephone consultations without video conferencing. |  |  |
| A systematic review on the effects of telerehabilitation on walking function in adult stroke survivors | What effects does telerehabilitation have on walking function, gait parameters and balance in the management of adult stroke survivors? | (P)opulation: Adult stroke survivors  (I)ntervention: tele-rehabilitation  (C)omparator: none  (O)utcome: walking function (gait parameters, endurance, balance and lower limb impairment)  (S)tudies: all except systematic reviews and meta-analysis studies |  |  | Studies using videoconferencing using the internet as a mode of delivery for rehabilitation. |  |  |
| Multidisciplinary team roles using telemedicine to prevent secondary stroke | How can a multidisciplinary team adopt a tele-medicine approach to post-stroke care to prevent post-stroke complications and a secondary stroke? | I: Use of telemedicine technology to provide real-time guidance from a multidisciplinary team to adults following stroke to reduce the incidence of complications and prevent secondary stroke. This includes phone support to patients and caregivers in the home and videoconferencing  C: Use of virtual reality, computer applications/software, or recordings prepared for either supervised or independent home use were excluded. The standard of care is an in-person office/clinic visit. |  |  | There are no restrictions on the types of study design eligible for inclusion |  |  |
| Stroke Telerehabilitation for Lower Extremity Recovery | The use of telerehabilitation for lower extremity recovery post-stroke. We aimed to quantitatively assess their effects on clinical outcomes of physical function and impairment, activities, and participation. | I: Telerehabilitation interventions (i.e., rehabilitation programs that are delivered virtually) focusing on lower extremity recovery. If mixed with in-person, at least 50% of the intervention must be provided via telerehabilitation. Studies can be completed during any phase of the post-stroke recovery process, and any physical or geographical location. Interventions that have only one session or primarily address problems related to upper extremity, cognition, communication, or swelling dysfunction will be excluded. |  |  |  |  |  |
| Telerehabilitation for stroke: a rapid review | In light of Covid-19, what are the options for clinicians regarding telerehabilitation for stroke in terms of the evidence regarding types of systems used, their technical requirements and costs, their translation into stroke practice including their uptake and sustainability (appointment attendance, duration of use), and facilitators and barriers to use. | outcomes related to satisfaction (patient, carer, clinician), usability and acceptability of telerehabilitation interventions. |  |  |  |  |  |
| Telerehabilitation interventions in patients after stroke: A systematic review | What is the efficacy of telerehabilitation therapy comparing with face-to-face therapy in patient with stroke? | The study will include different forms of telerehabilitation therapy including:  • Telephone  • Videoconferencing  • Virtual Reality (Non-Immersive applications) |  |  |  |  |  |
| The effect of Tele-rehabilitation on balance in stroke survivor. A systemic review and Meta-analysis | Does Tele-rehabilitation effective in individual with stroke to improve balance? | Participants/population  RCT measured the balance after the telerehabilitation in stroke patients  Intervention(s), exposure(s)  broad range of tele-rehabilitation interventions |  |  |  |  |  |
| The effectiveness of lower-limb wearable technology for improving activity and participation in adult stroke survivors: a systematic review | How effective are currently available wearable technologies for improving gait through rehabilitation in adults following stroke? | P: Adults post stroke  Types of study to be included  Inclusion: RCTs Exclusion: not RCTs, not quasi RCTs  Intervention(s), exposure(s)  Studies included:  Technological interventions that effect gait following stroke.  Technological interventions that involved wearable devices |  |  |  |  |  |
| The effectiveness of telerehabilitation on treatment adherence in poststroke patients: a systematic review. | What is the effectiveness of telerehabilitation on adherence to rehabilitation therapy in poststroke patients? | The inclusion criteria are adult patients, above the age of eighteen, that had experienced either SAH, ICH, Cerebral Ischemic Stroke, or Stroke not known if ischemic or haemorrhagic and are in the post-acute phase of stroke.  Inclusion criteria for studies would be any rehabilitation provisioned via telehealth technologies. In addition, trials that are conducted in person, but explicitly states the feasibility to be conducted remotely would be included as well |  |  |  |  |  |

# **SUPPLEMENTAL FILE 2: Full search strategies**

**Database**: Ovid MEDLINE(R) ALL 1946 to April 2023

**Date**: 19.04.2023

**Hits**: 836

| **#** | **Searches** | **Results** |
| --- | --- | --- |
| 1 | stroke/ | 120759 |
| 2 | stroke rehabilitation/ | 16129 |
| 3 | cerebral infarction/ | 23028 |
| 4 | hemorrhagic stroke/ | 342 |
| 5 | ischemic stroke/ | 5642 |
| 6 | embolic stroke/ | 245 |
| 7 | stroke, lacunar/ | 663 |
| 8 | thrombotic stroke/ | 37 |
| 9 | "intracranial embolism and thrombosis"/ | 8679 |
| 10 | cerebral hemorrhage/ | 35558 |
| 11 | intracranial thrombosis/ | 2927 |
| 12 | Ischemic Attack, Transient/ | 21384 |
| 13 | infarction, anterior cerebral artery/ | 272 |
| 14 | infarction, middle cerebral artery/ | 10107 |
| 15 | infarction, posterior cerebral artery/ | 294 |
| 16 | brain ischemia/ | 59081 |
| 17 | brain infarction/ | 4807 |
| 18 | hypoxia-ischemia, brain/ | 6616 |
| 19 | vertebrobasilar insufficiency/ | 3543 |
| 20 | brain stem infarctions/ | 827 |
| 21 | intracranial embolism/ | 5612 |
| 22 | sinus thrombosis, intracranial/ | 3354 |
| 23 | cavernous sinus thrombosis/ | 408 |
| 24 | lateral sinus thrombosis/ | 190 |
| 25 | sagittal sinus thrombosis/ | 214 |
| 26 | basal ganglia hemorrhage/ | 219 |
| 27 | putaminal hemorrhage/ | 150 |
| 28 | cerebral intraventricular hemorrhage/ | 335 |
| 29 | carotid artery thrombosis/ | 3151 |
| 30 | moyamoya disease/ | 3795 |
| 31 | intracranial aneurysm/ | 30634 |
| 32 | intracranial hemorrhages/ | 7975 |
| 33 | intracranial hemorrhage, hypertensive/ | 424 |
| 34 | hematoma, subdural/ | 7012 |
| 35 | hematoma, subdural, acute/ | 778 |
| 36 | hematoma, subdural, chronic/ | 1640 |
| 37 | hematoma, subdural, intracranial/ | 304 |
| 38 | hemiplegia/ | 11845 |
| 39 | (stroke* or poststroke* or ((cerebral or brain or intracranial) adj4 (infarct* or ischemi* or embolism* or thrombo* or aneurysm* or hemorrhag*)) or ((cerebrovascular or brain vascular) adj4 accident*) or apoplex* or (brain adj4 hypoxi* ischemi*) or (hemorrhag* adj4 (gangli* or putaminal*)) or ischemic attack* or TIA or (sinus adj4 thrombos*) or (carotid artery adj4 thrombo*) or thromboembolism* or carotid stenos* or moyamoya disease* or (vertebrobasilar adj4 insufficien*) or (hematoma adj4 (epidural* or subdural*)) or hemiplegi*).tw,kw,kf. | 467375 |
| 40 | or/1-39 | 550002 |
| 41 | Telerehabilitation/ | 779 |
| 42 | telemetry/ | 10094 |
| 43 | computer-assisted instruction/ | 12385 |
| 44 | computer communication networks/ | 13849 |
| 45 | Information Technology/ | 665 |
| 46 | computers, handheld/ | 3973 |
| 47 | mobile applications/ | 10031 |
| 48 | Remote Consultation/ | 5522 |
| 49 | telephone/ | 12964 |
| 50 | cell phone/ | 9623 |
| 51 | smartphone/ | 7764 |
| 52 | wireless technology/ | 4277 |
| 53 | Telecommunications/ | 5007 |
| 54 | decision making, computer-assisted/ | 2873 |
| 55 | therapy, computer-assisted/ | 6962 |
| 56 | information systems/ | 19293 |
| 57 | health information systems/ | 1534 |
| 58 | Remote Sensing Technology/ | 3665 |
| 59 | Distance Counseling/ | 76 |
| 60 | user-centered design/ | 142 |
| 61 | Computer-Aided Design/ | 17118 |
| 62 | information processing/ | 13381 |
| 63 | medical informatics applications/ | 2550 |
| 64 | video games/ | 6713 |
| 65 | videoconferencing/ | 2225 |
| 66 | electronic mail/ | 2895 |
| 67 | internet/ | 79156 |
| 68 | internet access/ | 136 |
| 69 | "internet use"/ | 340 |
| 70 | internet-based intervention/ | 942 |
| 71 | computer/ | 52271 |
| 72 | microcomputer/ | 14362 |
| 73 | smart glasses/ | 137 |
| 74 | minicomputer/ | 982 |
| 75 | activity tracker/ | 1014 |
| 76 | accelerometry/ | 7015 |
| 77 | Actigraphy/ | 4511 |
| 78 | neural networks, computer/ | 39242 |
| 79 | artificial intelligence/ | 31558 |
| 80 | machine learning/ | 24895 |
| 81 | supervised machine learning/ | 1275 |
| 82 | unsupervised machine learning/ | 685 |
| 83 | deep learning/ | 11024 |
| 84 | support vector machine/ | 9108 |
| 85 | databases, factual/ | 95001 |
| 86 | Data Science/ | 604 |
| 87 | fuzzy logic/ | 5028 |
| 88 | user-computer interface/ | 39035 |
| 89 | ambient intelligence/ | 36 |
| 90 | augmented reality/ | 799 |
| 91 | virtual reality/ | 4379 |
| 92 | software/ | 120306 |
| 93 | computers/ | 52271 |
| 94 | medical informatics/ | 12774 |
| 95 | text messaging/ | 4112 |
| 96 | webcasts as topic/ | 408 |
| 97 | Biomedical Technology/ | 7132 |
| 98 | algorithms/ | 287225 |
| 99 | Decision Trees/ | 11950 |
| 100 | wearable electronic devices/ | 6078 |
| 101 | fitness trackers/ | 1014 |
| 102 | expert systems/ | 3471 |
| 103 | knowledge bases/ | 1813 |
| 104 | (telerehab* or tele-rehab* or telemetry or telehealth or tele-health or teleconsult* or telesupervisi* or telemonitor* or telecare or tele-care or telehomecare or tele-homecare or telestroke* or tele-stroke* or telenursing or teleconferenc* or tele-conferenc* or teleOT or tele-OT or telepractice or tele-practice or teletherap* or tele-therap* or virtual rehab* or ((remote* or distanc* or distant or electronic* or video or tele) adj4 (consult* or supervisi* or coaching or counsel* or rehab* or therap* or treatment* or physio* or communication or care or specialist* or monitor*)) or videoconsult* or tele-coaching or e-counsel* or (remote adj4 (care* or sens*)) or mrehab* or m-rehab* or m-health or mhealth or e-therap* or etherap* or e-intervention* or erehab* or e-rehab* or ehealth or e-health or digital rehab*).tw,kw,kf. | 93423 |
| 105 | (((machine or deep) adj4 learning) or algorithm* or expert system* or knowledge base* or decision tree*).tw,kw,kf. | 395703 |
| 106 | (((activity or fitness) adj4 tracker*) or acceleromet* or pedomet* or actigraph* or actimetr* or (wearable adj4 (electronic device* or sensor*)) or ((step* or walk*) adj4 (count* or meter* or daily)) or ((physical or physiology* or perform* or fit* or train* or active* or endur* or exercise) adj4 (track* or monitor* or measur* or device* or app*))).tw,kw,kf. | 433563 |
| 107 | (computer* or laptop* or internet* or online or web-based or mobile* or app or apps or application* or telephone* or phone* or smartphone* or cellphone* or text messag* or SMS or personal digital assistant* or PDA* or smart watch* or smart glasses or Bluetooth or videoconferenc* or video-conferenc* or web conference* or webconference* or webcast* or electronic mail* or e-mail* or email* or video game* or exergame* or software* or hybrid or interactive or asynchron* or synchron* or ((cell* or smart* or mobile or android or internet or web or tablet*) adj4 device*)).tw,kw,kf. | 2655481 |
| 108 | (((information or wireless or remote* or biomedical or medical or health or digital* or communication) adj4 technolog*) or (digital* adj4 platform*) or ((artificial or ambient) adj4 intelligence) or AI or ((augmented or virtual) adj4 realit*)).tw,kw,kf. | 133583 |
|  | (((user-centered or computer-aided) adj4 design*) or telecommunication* or ((information or intelligent) adj4 system*) or information processing or (decision support adj4 (system* or tech*)) or ((wireless or media*) adj4 communication*) or ((health or nursing or medical) adj4 informatic*)).tw,kw,kf. | 126893 |
| 110 | or/41-109 | 3772152 |
| 111 | Home Care Agencies/ | 1394 |
| 112 | Home Environment/ | 186 |
| 113 | Home Health Aides/ | 772 |
| 114 | Home Nursing/ | 8649 |
| 115 | Housing/ | 19407 |
| 116 | Housing for the Elderly/ | 1652 |
| 117 | House Calls/ | 4020 |
| 118 | Residential Treatment/ | 3278 |
| 119 | home care services/ | 35612 |
| 120 | home care services, hospital-based/ | 1978 |
| 121 | home health nursing/ | 372 |
| 122 | homemaker services/ | 451 |
| 123 | Hospital to Home Transition/ | 18 |
| 124 | Independent Living/ | 9724 |
| 125 | (home* or housing or house* or residential* or residence* or ((independent or communit* or assisted) adj4 living) or dwell* or domestic* or domicile* or habitati* or abode*).tw,kf,kw. | 1015507 |
| 126 | or/111-125 | 1038006 |
| 127 | 40 and 110 and 126 | 3321 |
| 128 | randomized controlled trial.pt. | 568620 |
| 129 | controlled clinical trial.pt. | 94872 |
| 130 | (random* or rct*).tw,kw,kf. | 1327844 |
| 131 | placebo.ab. | 228278 |
| 132 | clinical trials as topic.sh. | 199892 |
| 133 | trial.ab,ti. | 697095 |
| 134 | (control adj6 group*).ab. | 619086 |
| 135 | or/128-134 | 2401153 |
| 136 | 127 and 135 | 836 |

**Database**: Ovid Embase 1974 to 2022 May 06

**Date**: 19.04.2023

**Hits**: 2344

| **#** | **Searches** | **Results** |
| --- | --- | --- |
| 1 | cerebrovascular accident/ | 248066 |
| 2 | stroke rehabilitation/ | 5701 |
| 3 | brain infarction/ | 58699 |
| 4 | brain stem infarction/ | 1564 |
| 5 | brain hemorrhage/ | 118208 |
| 6 | ischemic stroke/ | 8379 |
| 7 | acute ischemic stroke/ | 4210 |
| 8 | cardioembolic stroke/ | 4285 |
| 9 | lacunar stroke/ | 3899 |
| 10 | thromboembolism/ | 71964 |
| 11 | occlusive cerebrovascular disease/ | 10612 |
| 12 | transient ischemic attack/ | 42779 |
| 13 | cerebral artery disease/ | 5234 |
| 14 | brain ischemia/ | 154343 |
| 15 | vertebrobasilar insufficiency/ | 2890 |
| 16 | brain embolism/ | 7822 |
| 17 | cerebral sinus thrombosis/ | 7810 |
| 18 | cavernous sinus thrombosis/ | 1910 |
| 19 | lateral sinus thrombosis/ | 641 |
| 20 | sagittal sinus thrombosis/ | 835 |
| 21 | basal ganglion hemorrhage/ | 706 |
| 22 | putaminal hemorrhage/ | 215 |
| 23 | brain ventricle hemorrhage/ | 1136 |
| 24 | carotid artery thrombosis/ | 2354 |
| 25 | moyamoya disease/ | 7075 |
| 26 | intracranial aneurysm/ | 16675 |
| 27 | brain artery aneurysm/ | 17723 |
| 28 | brain artery aneurysm rupture/ | 3731 |
| 29 | unruptured intracranial aneurysm/ | 2324 |
| 30 | subdural hematoma/ | 19241 |
| 31 | hemiplegia/ | 19245 |
| 32 | (stroke* or poststroke* or ((cerebral or brain or intracranial) adj4 (infarct* or ischemi* or embolism* or thrombo* or aneurysm* or hemorrhag*)) or ((cerebrovascular or brain vascular) adj4 accident*) or apoplex* or (brain adj4 hypoxi* ischemi*) or (hemorrhag* adj4 (gangli* or putaminal*)) or ischemic attack* or TIA or (sinus adj4 thrombos*) or (carotid artery adj4 thrombo*) or thromboembolism* or carotid stenos* or moyamoya disease* or (vertebrobasilar adj4 insufficien*) or (hematoma adj4 (epidural* or subdural*)) or hemiplegi*).tw,kw,kf. | 699033 |
| 33 | or/1-32 | 916310 |
| 34 | telerehabilitation/ | 1765 |
| 35 | telemetry/ | 19438 |
| 36 | remote sensing/ | 12160 |
| 37 | telephone telemetry/ | 474 |
| 38 | computer network/ | 14191 |
| 39 | information technology/ | 12271 |
| 40 | computer/ | 78828 |
| 41 | digital computer/ | 2516 |
| 42 | microcomputer/ | 14792 |
| 43 | minicomputer/ | 759 |
| 44 | personal computer/ | 1016 |
| 45 | personal digital assistant/ | 1709 |
| 46 | desktop computer/ | 324 |
| 47 | laptop/ | 1082 |
| 48 | tablet computer/ | 2403 |
| 49 | wearable computer/ | 928 |
| 50 | wearable sensor/ | 1195 |
| 51 | smart watch/ | 390 |
| 52 | head-mounted display/ | 332 |
| 53 | smart glasses/ | 194 |
| 54 | virtual reality head mounted display/ | 413 |
| 55 | activity tracker/ | 1647 |
| 56 | actigraph/ | 1997 |
| 57 | pedometer/ | 2632 |
| 58 | mobile application/ | 17603 |
| 59 | mobile health application/ | 2511 |
| 60 | telephone/ | 41862 |
| 61 | mobile phone/ | 19996 |
| 62 | smartphone/ | 20165 |
| 63 | wireless communication/ | 6567 |
| 64 | telecommunication/ | 26983 |
| 65 | telehealth/ | 12956 |
| 66 | teleconsultation/ | 13280 |
| 67 | electronic consultation/ | 232 |
| 68 | telemonitoring/ | 4471 |
| 69 | video consultation/ | 476 |
| 70 | telenursing/ | 325 |
| 71 | decision support system/ | 25113 |
| 72 | computer assisted therapy/ | 4807 |
| 73 | information system/ | 39372 |
| 74 | medical information system/ | 22446 |
| 75 | nursing information system/ | 93 |
| 76 | online system/ | 29069 |
| 77 | health information system/ | 22446 |
| 78 | e-counseling/ | 370 |
| 79 | user-centered design/ | 203 |
| 80 | computer aided design/ | 23017 |
| 81 | medical informatics/ | 22135 |
| 82 | nursing informatics/ | 1659 |
| 83 | video game/ | 4959 |
| 84 | videoconferencing/ | 6914 |
| 85 | e-mail/ | 26593 |
| 86 | internet/ | 117016 |
| 87 | web-based intervention/ | 1673 |
| 88 | internet access/ | 1455 |
| 89 | "internet use"/ | 501 |
| 90 | information technology device/ | 584 |
| 91 | computer system/ | 26153 |
| 92 | exp personal computer/ | 10710 |
| 93 | artificial neural network/ | 43089 |
| 94 | accelerometer/ | 15205 |
| 95 | accelerometry/ | 8941 |
| 96 | actimetry/ | 10832 |
| 97 | artificial intelligence/ | 41187 |
| 98 | ambient intelligence/ | 34 |
| 99 | automated reasoning/ | 27 |
| 100 | machine learning/ | 59314 |
| 101 | fuzzy system/ | 4198 |
| 102 | algorithm/ | 319206 |
| 103 | learning algorithm/ | 10922 |
| 104 | semi supervised machine learning/ | 191 |
| 105 | supervised machine learning/ | 2861 |
| 106 | unsupervised machine learning/ | 1607 |
| 107 | deep learning/ | 23936 |
| 108 | support vector machine/ | 30163 |
| 109 | factual database/ | 27763 |
| 110 | data science/ | 998 |
| 111 | fuzzy logic/ | 4572 |
| 112 | computer interface/ | 34415 |
| 113 | human machine interface/ | 284 |
| 114 | augmented reality/ | 1310 |
| 115 | virtual reality/ | 22137 |
| 116 | software/ | 96881 |
| 117 | biomedical software/ | 2327 |
| 118 | software design/ | 1094 |
| 119 | rehabilitation software/ | 37 |
| 120 | self-care software/ | 20 |
| 121 | communication software/ | 568 |
| 122 | healthcare software/ | 379 |
| 123 | communication technology/ | 263 |
| 124 | text messaging/ | 6607 |
| 125 | web conferencing/ | 260 |
| 126 | webcast/ | 417 |
| 127 | medical technology/ | 35128 |
| 128 | "decision tree"/ | 17265 |
| 129 | expert system/ | 5617 |
| 130 | knowledge base/ | 6470 |
| 131 | digital technology/ | 1790 |
| 132 | (telerehab* or tele-rehab* or telemetry or telehealth or tele-health or teleconsult* or telesupervisi* or telemonitor* or telecare or tele-care or telehomecare or tele-homecare or telestroke* or tele-stroke* or telenursing or teleconferenc* or tele-conferenc* or teleOT or tele-OT or telepractice or tele-practice or teletherap* or tele-therap* or virtual rehab* or ((remote* or distanc* or distant or electronic* or video or tele) adj4 (consult* or supervisi* or coaching or counsel* or rehab* or therap* or treatment* or physio* or communication or care or specialist* or monitor*)) or videoconsult* or tele-coaching or e-counsel* or (remote adj4 (care* or sens*)) or mrehab* or m-rehab* or m-health or mhealth or e-therap* or etherap* or e-intervention* or erehab* or e-rehab* or ehealth or e-health or digital rehab*).tw,kw,kf. | 120814 |
| 133 | (((machine or deep) adj4 learning) or algorithm* or expert system* or knowledge base* or decision tree*).tw,kw,kf. | 497355 |
| 134 | (((activity or fitness) adj4 tracker*) or acceleromet* or pedomet* or actigraph* or actimetr* or (wearable adj4 (electronic device* or sensor*)) or ((step* or walk*) adj4 (count* or meter* or daily)) or ((physical or physiology* or perform* or fit* or train* or active* or endur* or exercise) adj4 (track* or monitor* or measur* or device* or app*))).tw,kw,kf. | 570224 |
| 135 | (computer* or laptop* or internet* or online or web-based or mobile* or app or apps or application* or telephone* or phone* or smartphone* or cellphone* or text messag* or SMS or personal digital assistant* or PDA* or smart watch* or smart glasses or Bluetooth or videoconferenc* or video-conferenc* or web conference* or webconference* or webcast* or electronic mail* or e-mail* or email* or video game* or exergame* or software* or hybrid or interactive or asynchron* or synchron* or ((cell* or smart* or mobile or android or internet or web or tablet*) adj4 device*)).tw,kw,kf. | 3259675 |
| 136 | (((information or wireless or remote* or biomedical or medical or health or digital* or communication) adj4 technolog*) or (digital* adj4 platform*) or ((artificial or ambient) adj4 intelligence) or AI or ((augmented or virtual) adj4 realit*)).tw,kw,kf. | 169144 |
| 137 | (((user-centered or computer-aided) adj4 design*) or telecommunication* or ((information or intelligent) adj4 system*) or information processing or (decision support adj4 (system* or tech*)) or ((wireless or media*) adj4 communication*) or ((health or nursing or medical) adj4 informatic*)).tw,kw,kf. | 155386 |
| 138 | or/34-137 | 4616071 |
| 139 | home care/ | 66047 |
| 140 | home health agency/ | 388 |
| 141 | home monitoring/ | 5199 |
| 142 | home physiotherapy/ | 380 |
| 143 | home rehabilitation/ | 924 |
| 144 | home visit/ | 4125 |
| 145 | respite care/ | 1235 |
| 146 | visiting nursing service/ | 221 |
| 147 | community living/ | 5355 |
| 148 | home environment/ | 5913 |
| 149 | housing/ | 27838 |
| 150 | home for the aged/ | 11319 |
| 151 | residential care/ | 12733 |
| 152 | independent living/ | 6349 |
| 153 | home safety/ | 725 |
| 154 | home accident/ | 3190 |
| 155 | residential home/ | 7538 |
| 156 | home range/ | 669 |
| 157 | residential area/ | 7587 |
| 158 | home stress/ | 106 |
| 159 | community dwelling person/ | 7864 |
| 160 | (home* or housing or house* or residential* or residence* or ((independent or communit* or assisted) adj4 living) or dwell* or domestic* or domicile* or habitati* or abode*).tw,kf,kw. | 1302856 |
| 161 | or/139-160 | 1347828 |
| 162 | 33 and 138 and 161 | 6610 |
| 163 | exp randomized controlled trial/ | 708550 |
| 164 | "randomized controlled trial (topic)"/ | 225729 |
| 165 | randomization/ | 93723 |
| 166 | controlled clinical trial/ | 465536 |
| 167 | "controlled clinical trial (topic)"/ | 12321 |
| 168 | placebo/ | 379874 |
| 169 | placebo effect/ | 6908 |
| 170 | phase 1 clinical trial/ | 64117 |
| 171 | phase 2 clinical trial/ | 96477 |
| 172 | phase 3 clinical trial/ | 60337 |
| 173 | phase 4 clinical trial/ | 4742 |
| 174 | clinical trial protocol/ | 1787 |
| 175 | control group/ | 109940 |
| 176 | crossover procedure/ | 70225 |
| 177 | double blind procedure/ | 194618 |
| 178 | single blind procedure/ | 46022 |
| 179 | triple blind procedure/ | 329 |
| 180 | (random$ or RCT or RCTs).tw. | 1800529 |
| 181 | (controlled adj5 (trial$ or stud$)).tw. | 597403 |
| 182 | (clinical$ adj5 trial$).tw. | 671763 |
| 183 | ((control or treatment or experiment$ or intervention) adj5 (group$ or subject$ or patient$)).tw. | 2586000 |
| 184 | (quasi-random$ or quasi random$ or pseudo-random$ or pseudo random$).tw. | 7453 |
| 185 | ((control or experiment$ or conservative) adj5 (treatment or therapy or procedure or manage$)).tw. | 333054 |
| 186 | ((singl$ or doubl$ or tripl$ or trebl$) adj5 (blind$ or mask$)).tw. | 267522 |
| 187 | (cross-over or cross over or crossover).tw. | 116084 |
| 188 | (placebo$ or sham).tw. | 469441 |
| 189 | trial.ti. | 357492 |
| 190 | (assign$ or allocat$).tw. | 619826 |
| 191 | controls.tw. | 1283511 |
| 192 | or/163-191 | 6304130 |
| 193 | 162 and 192 | 2344 |

**Database:** The Cochrane Library via Wiley

**Date:**  19.04.2023

**Hits:** 1636

| **ID** | **Search** | **Hits** |
| --- | --- | --- |
| #1 | [mh ^stroke] | 10320 |
| #2 | [mh ^"stroke rehabilitation"] | 2894 |
| #3 | [mh ^"cerebral infarction"] | 1036 |
| #4 | [mh ^"hemorrhagic stroke"] | 18 |
| #5 | [mh ^"ischemic stroke"] | 253 |
| #6 | [mh ^"embolic stroke"] | 10 |
| #7 | [mh ^"stroke, lacunar"] | 47 |
| #8 | [mh ^"thrombotic stroke"] | 2 |
| #9 | [mh ^"intracranial embolism and thrombosis"] | 86 |
| #10 | [mh ^"cerebral hemorrhage"] | 1042 |
| #11 | [mh ^"intracranial thrombosis"] | 43 |
| #12 | [mh ^"Ischemic Attack, Transient"] | 816 |
| #13 | [mh ^"infarction, anterior cerebral artery"] | 7 |
| #14 | [mh ^"infarction, middle cerebral artery"] | 145 |
| #15 | [mh ^"infarction, posterior cerebral artery"] | 4 |
| #16 | [mh ^"brain ischemia"] | 1839 |
| #17 | [mh ^"brain infarction"] | 117 |
| #18 | [mh ^”hypoxia-ischemia, brain”] | 245 |
| #19 | [mh ^"vertebrobasilar insufficiency"] | 61 |
| #20 | [mh ^"brain stem infarctions"] | 14 |
| #21 | [mh ^"intracranial embolism"] | 177 |
| #22 | [mh ^"sinus thrombosis, intracranial"] | 19 |
| #23 | [mh ^"cavernous sinus thrombosis"] | 0 |
| #24 | [mh ^"lateral sinus thrombosis"] | 2 |
| #25 | [mh ^"sagittal sinus thrombosis"] | 0 |
| #26 | [mh ^"basal ganglia hemorrhage"] | 12 |
| #27 | [mh ^"putaminal hemorrhage"] | 7 |
| #28 | [mh ^"cerebral intraventricular hemorrhage"] | 22 |
| #29 | [mh ^"carotid artery thrombosis"] | 20 |
| #30 | [mh ^"moyamoya disease"] | 27 |
| #31 | [mh ^"intracranial aneurysm"] | 473 |
| #32 | [mh ^"intracranial hemorrhages"] | 301 |
| #33 | [mh ^"intracranial hemorrhage, hypertensive"] | 49 |
| #34 | [mh ^"hematoma, subdural"] | 80 |
| #35 | [mh ^"hematoma, subdural, acute"] | 11 |
| #36 | [mh ^"hematoma, subdural, chronic"] | 101 |
| #37 | [mh ^"hematoma, subdural, intracranial"] | 8 |
| #38 | [mh ^hemiplegia] | 792 |
| #39 | (stroke* or poststroke* or ((cerebral or brain or intracranial) NEAR/4 (infarct* or ischemi* or embolism* or thrombo* or aneurysm* or hemorrhag*)) or ((cerebrovascular or brain NEXT vascular) NEAR/4 accident*) or apoplex* or (brain NEAR/4 (hypoxi* NEXT ischemi*)) or (hemorrhag* NEAR/4 (gangli* or putaminal*)) or (ischemic NEXT attack*) or TIA or (sinus NEAR/4 thrombos*) or ((carotid NEXT artery) NEAR/4 thrombo*) or thromboembolism* or (carotid NEXT stenos*) or (moyamoya NEXT disease*) or (vertebrobasilar NEAR/4 insufficien*) or (hematoma NEAR/4 (epidural* or subdural*)) or hemiplegi*):ti,ab,kw | 83506 |
| #40 | {OR #1-#39} | 83506 |
| #41 | [mh ^Telerehabilitation] | 167 |
| #42 | [mh ^telemetry] | 259 |
| #43 | [mh ^”computer-assisted instruction”] | 1264 |
| #44 | [mh ^”computer communication networks”] | 54 |
| #45 | [mh ^”Information Technology”] | 16 |
| #46 | [mh ^”computers, handheld”] | 309 |
| #47 | [mh ^”mobile applications”] | 1054 |
| #48 | [mh ^”Remote Consultation”] | 387 |
| #49 | [mh ^telephone] | 2336 |
| #50 | [mh ^”cell phone”] | 779 |
| #51 | [mh ^smartphone] | 639 |
| #52 | [mh ^”wireless technology”] | 45 |
| #53 | [mh ^Telecommunications] | 89 |
| #54 | [mh ^”decision making, computer-assisted”] | 137 |
| #55 | [mh ^”therapy, computer-assisted”] | 1372 |
| #56 | [mh ^”information systems”] | 63 |
| #57 | [mh ^”health information systems”] | 13 |
| #58 | [mh ^”Remote Sensing Technology”] | 51 |
| #59 | [mh ^”Distance Counseling”] | 23 |
| #60 | [mh ^”user-centered design”] | 3 |
| #61 | [mh ^”Computer-Aided Design”] | 242 |
| #62 | [mh ^”information processing”] | 92 |
| #63 | [mh ^”medical informatics applications”] | 24 |
| #64 | [mh ^”video games”] | 798 |
| #65 | [mh ^”videoconferencing”] | 218 |
| #66 | [mh ^”electronic mail”] | 360 |
| #67 | [mh ^internet] | 4135 |
| #68 | [mh ^”internet access”] | 4 |
| #69 | [mh ^"internet use"] | 1 |
| #70 | [mh ^”internet-based intervention”] | 336 |
| #71 | [mh ^computer] | 592 |
| #72 | [mh ^microcomputer] | 248 |
| #73 | [mh ^”smart glasses”] | 8 |
| #74 | [mh ^minicomputer] | 6 |
| #75 | [mh ^”activity tracker”] | 148 |
| #76 | [mh ^accelerometry] | 545 |
| #77 | [mh ^Actigraphy] | 544 |
| #78 | [mh ^”neural networks, computer”] | 133 |
| #79 | [mh ^”artificial intelligence”] | 226 |
| #80 | [mh ^”machine learning”] | 149 |
| #81 | [mh ^”supervised machine learning”] | 4 |
| #82 | [mh ^”unsupervised machine learning”] | 1 |
| #83 | [mh ^”deep learning”] | 49 |
| #84 | [mh ^”support vector machine”] | 21 |
| #85 | [mh ^”databases, factual”] | 313 |
| #86 | [mh ^”Data Science”] | 0 |
| #87 | [mh ^”fuzzy logic”] | 38 |
| #88 | [mh ^”user-computer interface”] | 1292 |
| #89 | [mh ^”ambient intelligence”] | 0 |
| #90 | [mh ^”augmented reality”] | 21 |
| #91 | [mh ^”virtual reality”] | 462 |
| #92 | [mh ^software] | 1008 |
| #93 | [mh ^computers] | 592 |
| #94 | [mh ^”medical informatics”] | 83 |
| #95 | [mh ^”text messaging”] | 1119 |
| #96 | [mh ^”webcasts as topic”] | 27 |
| #97 | [mh ^”Biomedical Technology”] | 22 |
| #98 | [mh ^algorithms] | 3481 |
| #99 | [mh ^”Decision Trees”] | 167 |
| #100 | [mh ^”wearable electronic devices”] | 131 |
| #101 | [mh ^”fitness trackers”] | 148 |
| #102 | [mh ^”expert systems”] | 59 |
| #103 | [mh ^”knowledge bases”] | 12 |
| #104 | (telerehab* or tele-rehab* or telemetry or telehealth or tele-health or teleconsult* or telesupervisi* or telemonitor* or telecare or tele-care or telehomecare or tele-homecare or telestroke* or tele-stroke* or telenursing or teleconferenc* or tele-conferenc* or teleOT or tele-OT or telepractice or tele-practice or teletherap* or tele-therap* or (virtual NEXT rehab*) or ((remote* or distanc* or distant or electronic* or video or tele) NEAR/4 (consult* or supervisi* or coaching or counsel* or rehab* or therap* or treatment* or physio* or communication or care or specialist* or monitor*)) or videoconsult* or tele-coaching or e-counsel* or (remote NEAR/4 (care* or sens*)) or mrehab* or m-rehab* or m-health or mhealth or e-therap* or etherap* or e-intervention* or erehab* or e-rehab* or ehealth or e-health or (digital NEXT rehab*)):ti,ab,kw | 18267 |
| #105 | (((machine or deep) NEAR/4 learning) or algorithm* or (expert NEXT system*) or (knowledge NEXT base*) or (decision NEXT tree*)):ti,ab,kw | 17950 |
| #106 | (((activity or fitness) NEAR/4 tracker*) or acceleromet* or pedomet* or actigraph* or actimetr* or (wearable NEAR/4 ((electronic NEXT device*) or sensor*)) or ((step* or walk*) NEAR/4 (count* or meter* or daily)) or ((physical or physiology* or perform* or fit* or train* or active* or endur* or exercise) NEAR/4 (track* or monitor* or measur* or device* or app*))):ti,ab,kw | 72107 |
| #107 | (computer* or laptop* or internet* or online or web-based or mobile* or app or apps or application* or telephone* or phone* or smartphone* or cellphone* or (text NEXT messag*) or SMS or (personal NEXT digital NEXT assistant*) or PDA* or (smart NEXT watch*) or (smart NEXT glasses) or Bluetooth or videoconferenc* or video-conferenc* or (web NEXT conference*) or webconference* or webcast* or (electronic NEXT mail*) or e-mail* or email* or (video NEXT game*) or exergame* or software* or hybrid or interactive or asynchron* or synchron* or ((cell* or smart* or mobile or android or internet or web or tablet*) NEAR/4 device*)):ti,ab,kw | 208980 |
| #108 | (((information or wireless or remote* or biomedical or medical or health or digital* or communication) NEAR/4 technolog*) or (digital* NEAR/4 platform*) or ((artificial or ambient) NEAR/4 intelligence) or AI or ((augmented or virtual) NEAR/4 realit*)):ti,ab,kw | 15471 |
| #109 | (((user-centered or computer-aided) NEAR/4 design*) or telecommunication* or ((information or intelligent) NEAR/4 system*) or (information NEXT processing) or ((decision NEXT support) NEAR/4 (system* or tech*)) or ((wireless or media*) NEAR/4 communication*) or ((health or nursing or medical) NEAR/4 informatic*)):ti,ab,kw | 12587 |
| #110 | {OR #41-#109} | 295584 |
| #111 | [mh ^”Home Care Agencies”] | 13 |
| #112 | [mh ^”Home Environment”] | 0 |
| #113 | [mh ^”Home Health Aides”] | 23 |
| #114 | [mh ^”Home Nursing”] | 284 |
| #115 | [mh ^Housing] | 330 |
| #116 | [mh ^”Housing for the Elderly”] | 41 |
| #117 | [mh ^”House Calls”] | 583 |
| #118 | [mh ^”Residential Treatment”] | 178 |
| #119 | [mh ^”home care services”] | 1945 |
| #120 | [mh ^”home care services, hospital-based”] | 247 |
| #121 | [mh ^”home health nursing”] | 8 |
| #122 | [mh ^”homemaker services”] | 6 |
| #123 | [mh ^”Hospital to Home Transition”] | 0 |
| #124 | [mh ^”Independent Living”] | 655 |
| #125 | (home* or housing or house* or residential* or residence* or ((independent or communit* or assisted) NEAR/4 living) or dwell* or domestic* or domicile* or habitati* or abode*):ti,ab,kw | 88065 |
| #126 | {OR #111-#125} | 88065 |
| #127 | #40 and #110 and #126 | 1636 |

**Database:** Web of Science via Clarivate (Science Citation Index Expanded 1987-present; Social Sciences Citation Index 1987-present; Social Sciences Citation Index 1987-present; Arts & Humanities Citation Index 1987-present; Emerging Sources Citation Index 2017-present)

**Date:**  19.04.2023

**Hits:** 1101

| **#** | **Search** | **Results** |
| --- | --- | --- |
| 1 | TS=(stroke* or poststroke* or ((cerebral or brain or intracranial) NEAR/3 (infarct* or ischemi* or embolism* or thrombo* or aneurysm* or hemorrhag*)) or ((cerebrovascular or “brain vascular”) NEAR/3 accident*) or apoplex* or (brain NEAR/3 “hypoxi* ischemi*”) or (hemorrhag* NEAR/3 (gangli* or putaminal*)) or “ischemic attack*” or TIA or (sinus NEAR/3 thrombos*) or (“carotid artery” NEAR/3 thrombo*) or thromboembolism* or “carotid stenos*” or “moyamoya disease*” or (vertebrobasilar NEAR/3 insufficien*) or (hematoma NEAR/3 (epidural* or subdural*)) or hemiplegi*) | 554,705 |
| 2 | TS=(telerehab* or “tele-rehab*” or telemetry or telehealth or “tele-health” or teleconsult* or telesupervisi* or telemonitor* or telecare or “tele-care” or telehomecare or “tele-homecare” or telestroke* or “tele-stroke*” or telenursing or teleconferenc* or “tele-conferenc*” or teleOT or “tele-OT” or telepractice or “tele-practice” or teletherap* or “tele-therap*” or “virtual rehab*” or ((remote* or distanc* or distant or electronic* or video or tele) NEAR/3 (consult* or supervisi* or coaching or counsel* or rehab* or therap* or treatment* or physio* or communication or care or specialist* or monitor*)) or videoconsult* or “tele-coaching” or “e-counsel*” or (remote NEAR/3 (care* or sens*)) or mrehab* or “m-rehab*” or “m-health” or mhealth or “e-therap*” or etherap* or e-intervention* or erehab* or “e-rehab*” or ehealth or “e-health” or “digital rehab*”) | 217,675 |
| 3 | TS=(((machine or deep) NEAR/3 learning) or algorithm* or “expert system*” or “knowledge base*” or “decision tree*”) | 1,643,662 |
| 4 | TS=(((activity or fitness) NEAR/3 tracker*) or acceleromet* or pedomet* or actigraph* or actimetr* or (wearable NEAR/3 (“electronic device*” or sensor*)) or ((step* or walk*) NEAR/3 (count* or meter* or daily)) or ((physical or physiology* or perform* or fit* or train* or active* or endur* or exercise) NEAR/3 (track* or monitor* or measur* or device* or app*))) | 840,044 |
| 5 | TS=(computer* or laptop* or internet* or online or “web-based” or mobile* or app or apps or application* or telephone* or phone* or smartphone* or cellphone* or “text messag*” or SMS or “personal digital assistant*” or PDA* or “smart watch*” or “smart glasses” or Bluetooth or videoconferenc* or “video-conferenc*” or “web conference*” or webconference* or webcast* or “electronic mail*” or “e-mail*” or email* or “video game*” or exergame* or software* or hybrid or interactive or asynchron* or synchron* or ((cell* or smart* or mobile or android or internet or web or tablet*) NEAR/3 device*)) | 6,143,106 |
| 6 | TS=(((information or wireless or remote* or biomedical or medical or health or digital* or communication) NEAR/3 technolog*) or (digital* NEAR/3 platform*) or ((artificial or ambient) NEAR/3 intelligence) or AI or ((augmented or virtual) NEAR/3 realit*)) | 311,602 |
| 7 | TS=(((“user-centered” or “computer-aided”) NEAR/3 design*) or telecommunication* or ((information or intelligent) NEAR/3 system*) or “information processing” or (“decision support” NEAR/3 (system* or tech*)) or ((wireless or media*) NEAR/3 communication*) or ((health or nursing or medical) NEAR/3 informatic*)) | 347,160 |
| 8 | #7 OR #6 OR #5 OR #4 OR #3 OR #2 | 8,126,081 |
| 9 | TS=(home* or housing or house* or residential* or residence* or ((independent or communit* or assisted) NEAR/3 living) or dwell* or domestic* or domicile* or habitati* or abode*) | 1,645,867 |
| 10 | #9 AND #8 AND #1 | 3,866 |
| 11 | TS=(placebo* OR random* OR RCT* OR (blind* AND (single OR double OR treble OR triple))) OR TI=(trial) OR AB=(trial OR (control NEAR/5 group*)) | 3,226,557 |
| 12 | #11 AND #10 | 1,101 |

**Database:** Scopus via Elsevier

**Date:**  19.04.2023

**Hits:** 1,639

| **#** | **Search** | **Results** |
| --- | --- | --- |
| #1 | TITLE-ABS-KEY(stroke* or poststroke* or ((cerebral or brain or intracranial) W/3 (infarct* or ischemi* or embolism* or thrombo* or aneurysm* or hemorrhag*)) or ((cerebrovascular or “brain vascular”) W/3 accident*) or apoplex* or (brain W/3 “hypoxi* ischemi*”) or (hemorrhag* W/3 (gangli* or putaminal*)) or “ischemic attack*” or TIA or (sinus W/3 thrombos*) or (“carotid artery” W/3 thrombo*) or thromboembolism* or “carotid stenos*” or “moyamoya disease*” or (vertebrobasilar W/3 insufficien*) or (hematoma W/3 (epidural* or subdural*)) or hemiplegi*) | 919,419 |
| #2 | TITLE-ABS-KEY(telerehab* or “tele-rehab*” or telemetry or telehealth or “tele-health” or teleconsult* or telesupervisi* or telemonitor* or telecare or “tele-care” or telehomecare or “tele-homecare” or telestroke* or “tele-stroke*” or telenursing or teleconferenc* or “tele-conferenc*” or teleOT or “tele-OT” or telepractice or “tele-practice” or teletherap* or “tele-therap*” or “virtual rehab*” or ((remote* or distanc* or distant or electronic* or video or tele) W/3 (consult* or supervisi* or coaching or counsel* or rehab* or therap* or treatment* or physio* or communication or care or specialist* or monitor*)) or videoconsult* or “tele-coaching” or “e-counsel*” or (remote W/3 (care* or sens*)) or mrehab* or “m-rehab*” or “m-health” or mhealth or “e-therap*” or etherap* or e-intervention* or erehab* or “e-rehab*” or ehealth or “e-health” or “digital rehab*”) | 547,032 |
| #3 | TITLE-ABS-KEY(((machine or deep) W/3 learning) or algorithm* or “expert system*” or “knowledge base*” or “decision tree*”) | 4,011,453 |
| #4 | TITLE-ABS-KEY(((activity or fitness) W/3 tracker*) or acceleromet* or pedomet* or actigraph* or actimetr* or (wearable W/3 (“electronic device*” or sensor*)) or ((step* or walk*) W/3 (count* or meter* or daily)) or ((physical or physiology* or perform* or fit* or train* or active* or endur* or exercise) W/3 (track* or monitor* or measur* or device* or app*))) | 1,568,039 |
| #5 | TITLE-ABS-KEY(computer* or laptop* or internet* or online or “web-based” or mobile* or app or apps or application* or telephone* or phone* or smartphone* or cellphone* or “text messag*” or SMS or “personal digital assistant*” or PDA* or “smart watch*” or “smart glasses” or Bluetooth or videoconferenc* or “video-conferenc*” or “web conference*” or webconference* or webcast* or “electronic mail*” or “e-mail*” or email* or “video game*” or exergame* or software* or hybrid or interactive or asynchron* or synchron* or ((cell* or smart* or mobile or android or internet or web or tablet*) W/3 device*)) | 15,112,586 |
| #6 | TITLE-ABS-KEY(((information or wireless or remote* or biomedical or medical or health or digital* or communication) W/3 technolog*) or (digital* W/3 platform*) or ((artificial or ambient) W/3 intelligence) or AI or ((augmented or virtual) W/3 realit*)) | 1,232,432 |
| #7 | TITLE-ABS-KEY(((“user-centered” or “computer-aided”) W/3 design*) or telecommunication* or ((information or intelligent) W/3 system*) or “information processing” or (“decision support” W/3 (system* or tech*)) or ((wireless or media*) W/3 communication*) or ((health or nursing or medical) W/3 informatic*)) | 1,945,013 |
| #8 | #7 OR #6 OR #5 OR #4 OR #3 OR #2 | 19,244,247 |
| #9 | TITLE-ABS-KEY(home* or housing or house* or residential* or residence* or ((independent or communit* or assisted) W/3 living) or dwell* or domestic* or domicile* or habitati* or abode*) | 2,694,625 |
| #10 | #9 AND #8 AND #1 | 7,923 |
| #11 | TITLE-ABS-KEY({clinical-trial} OR {controlled-trial} OR randomi* OR randomly OR (random W/4 (allocat* OR distribut* OR assign*)) OR {placebo} OR {trial} OR (control W/5 group*) OR {subgroups}) OR TITLE (RCT) | 3,901,332 |
| #12 | #11 AND #10 | 1,639 |

**Database:** CINAHL via EBSCOhost

**Date:**  19.04.2023

**Hits:** 604

| **#** | **Query** | **Results** |
| --- | --- | --- |
| S1 | (MH "Stroke") | 75,891 |
| S2 | (MH "Stroke Patients") | 7,110 |
| S3 | (MH "Cerebral Infarction") | 161 |
| S4 | (MH "Hemorrhagic Stroke") | 77 |
| S5 | (MH "Embolic Stroke") | 16 |
| S6 | (MH "Stroke, Lacunar") | 292 |
| S7 | (MH "Ischemic Stroke") | 566 |
| S8 | (MH "Cerebral Hemorrhage") | 7,657 |
| S9 | (MH "Intracranial Embolism and Thrombosis") | 1,256 |
| S10 | (MH "Intracranial Thrombosis") | 682 |
| S11 | (MH "Intracranial Embolism") | 922 |
| S12 | (MH "Sinus Thrombosis, Intracranial") | 518 |
| S13 | (MH "Cavernous Sinus Thrombosis") | 93 |
| S14 | (MH "Intracranial Hemorrhage") | 3,649 |
| S15 | (MH "Hematoma, Subdural") | 1,140 |
| S16 | (MH "Hematoma, Subdural, Acute") | 151 |
| S17 | (MH "Hematoma, Subdural, Chronic") | 192 |
| S18 | (MH "Basal Ganglia Hemorrhage") | 50 |
| S19 | (MH "Cerebral Ischemia") | 13,897 |
| S20 | (MH "Cerebral Ischemia, Transient") | 5,103 |
| S21 | (MH "Hypoxia-Ischemia, Brain") | 1,429 |
| S22 | (MH "Carotid Artery Thrombosis") | 215 |
| S23 | (MH "Moyamoya Disease") | 624 |
| S24 | (MH "Cerebral Aneurysm") | 4,386 |
| S25 | (MH "Hemiplegia") | 6,509 |
| S26 | TI ((stroke* or poststroke* or ((cerebral or brain or intracranial) N3 (infarct* or ischemi* or embolism* or thrombo* or aneurysm* or hemorrhag*)) or ((cerebrovascular or “brain vascular”) N3 accident*) or apoplex* or (brain N3 hypoxi* ischemi*) or (hemorrhag* N3 (gangli* or putaminal*)) or “ischemic attack*” or TIA or (sinus N3 thrombos*) or (“carotid artery” N3 thrombo*) or thromboembolism* or “carotid stenos*” or “moyamoya disease*” or (vertebrobasilar N3 insufficien*) or (hematoma N3 (epidural* or subdural*)) or hemiplegi*) ) OR AB ((stroke* or poststroke* or ((cerebral or brain or intracranial) N3 (infarct* or ischemi* or embolism* or thrombo* or aneurysm* or hemorrhag*)) or ((cerebrovascular or “brain vascular”) N3 accident*) or apoplex* or (brain N3 hypoxi* ischemi*) or (hemorrhag* N3 (gangli* or putaminal*)) or “ischemic attack*” or TIA or (sinus N3 thrombos*) or (“carotid artery” N3 thrombo*) or thromboembolism* or “carotid stenos*” or “moyamoya disease*” or (vertebrobasilar N3 insufficien*) or (hematoma N3 (epidural* or subdural*)) or hemiplegi*) ) | 140,567 |
| S27 | S1 OR S2 OR S3 OR S4 OR S5 OR S6 OR S7 OR S8 OR S9 OR S10 OR S11 OR S12 OR S13 OR S14 OR S15 OR S16 OR S17 OR S18 OR S19 OR S20 OR S21 OR S22 OR S23 OR S24 OR S25 OR S26 | 168,788 |
| S28 | (MH "Telerehabilitation") | 436 |
| S29 | (MH "Telemetry") | 2,179 |
| S30 | (MH "Computer Assisted Instruction") | 8,218 |
| S31 | (MH "Computer Environment") | 263 |
| S32 | (MH "Computer Communication Networks") | 3,026 |
| S33 | (MH "Digital Technology") | 983 |
| S34 | (MH "Information Technology") | 14,847 |
| S35 | (MH "Computers, Hand-Held") | 4,653 |
| S36 | (MH "Computers, Portable") | 1,647 |
| S37 | (MH "Microcomputers") | 1,516 |
| S38 | (MH "Smartphone") | 3,633 |
| S39 | (MH "Computers and Computerization") | 10,840 |
| S40 | (MH "Computer Systems") | 2,042 |
| S41 | (MH "Mobile Applications") | 10,628 |
| S42 | (MH "Telehealth") | 12,013 |
| S43 | (MH "Remote Consultation") | 2,822 |
| S44 | (MH "Telephone") | 17,507 |
| S45 | (MH "Cellular Phone") | 2,136 |
| S46 | (MH "Text Messaging") | 3,777 |
| S47 | (MH "Videoconferencing") | 2,570 |
| S48 | (MH "Webcasts") | 743 |
| S49 | (MH "Telecommunications") | 2,593 |
| S50 | (MH "Internet") | 53,475 |
| S51 | (MH "Internet Access") | 306 |
| S52 | (MH "Internet-Based Intervention") | 405 |
| S53 | (MH "Wireless Communications") | 12,188 |
| S54 | (MH "Therapy, Computer Assisted") | 5,480 |
| S55 | (MH "Decision Making, Computer Assisted") | 1,372 |
| S56 | (MH "Health Information Systems") | 3,546 |
| S57 | (MH "Information Systems") | 4,860 |
| S58 | (MH "Computer Aided Design") | 3,938 |
| S59 | (MH "Medical Informatics") | 5,424 |
| S60 | (MH "Nursing Informatics") | 3,143 |
| S61 | (MH "Health Informatics") | 4,393 |
| S62 | (MH "Video Games") | 5,279 |
| S63 | (MH "Exergames") | 255 |
| S64 | (MH "Email") | 7,001 |
| S65 | (MH "Smart Glasses") | 22 |
| S66 | (MH "Accelerometers") | 2,732 |
| S67 | (MH "Wearable Sensors") | 2,486 |
| S68 | (MH "Pedometers") | 1,466 |
| S69 | (MH "Minicomputers") | 39 |
| S70 | (MH "Fitness Trackers") | 284 |
| S71 | (MH "Accelerometry") | 5,093 |
| S72 | (MH "Actigraphy") | 1,568 |
| S73 | (MH "Neural Networks (Computer)") | 3,049 |
| S74 | (MH "Artificial Intelligence") | 6,937 |
| S75 | (MH "Knowbots") | 58 |
| S76 | (MH "Expert Systems") | 534 |
| S77 | (MH "Knowledge Bases") | 727 |
| S78 | (MH "Machine Learning") | 3,094 |
| S79 | (MH "Deep Learning") | 982 |
| S80 | (MH "Support Vector Machine") | 152 |
| S81 | (MH "Data Science") | 187 |
| S82 | (MH "User-Computer Interface") | 11,110 |
| S83 | (MH "Software") | 31,239 |
| S84 | (MH "Communications Software") | 312 |
| S85 | (MH "Patient Portals") | 198 |
| S86 | (MH "Virtual Reality") | 6,522 |
| S87 | (MH "Augmented Reality") | 315 |
| S88 | (MH "Algorithms") | 42,604 |
| S89 | (MH "Decision Trees") | 2,767 |
| S90 | (MH "Communications Media") | 11,481 |
| S91 | (MH "Online Systems") | 1,868 |
| S92 | TI ( (telerehab* or tele-rehab* or telemetry or telehealth or tele-health or teleconsult* or telesupervisi* or telemonitor* or telecare or tele-care or telehomecare or tele-homecare or telestroke* or tele-stroke* or telenursing or teleconferenc* or tele-conferenc* or teleOT or tele-OT or telepractice or tele-practice or teletherap* or tele-therap* or “virtual rehab*” or ((remote* or distanc* or distant or electronic* or video or tele) N3 (consult* or supervisi* or coaching or counsel* or rehab* or therap* or treatment* or physio* or communication or care or specialist* or monitor*)) or videoconsult* or tele-coaching or e-counsel* or (remote N3 (care* or sens*)) or mrehab* or m-rehab* or m-health or mhealth or e-therap* or etherap* or e-intervention* or erehab* or e-rehab* or ehealth or e-health or digital rehab*) ) OR AB ( (telerehab* or tele-rehab* or telemetry or telehealth or tele-health or teleconsult* or telesupervisi* or telemonitor* or telecare or tele-care or telehomecare or tele-homecare or telestroke* or tele-stroke* or telenursing or teleconferenc* or tele-conferenc* or teleOT or tele-OT or telepractice or tele-practice or teletherap* or tele-therap* or “virtual rehab*” or ((remote* or distanc* or distant or electronic* or video or tele) N3 (consult* or supervisi* or coaching or counsel* or rehab* or therap* or treatment* or physio* or communication or care or specialist* or monitor*)) or videoconsult* or tele-coaching or e-counsel* or (remote N3 (care* or sens*)) or mrehab* or m-rehab* or m-health or mhealth or e-therap* or etherap* or e-intervention* or erehab* or e-rehab* or ehealth or e-health or digital rehab*) ) | 34,489 |
| S93 | TI ( (((machine or deep) N3 learning) or algorithm* or “expert system*” or “knowledge base*” or “decision tree*”) ) OR AB ( (((machine or deep) N3 learning) or algorithm* or “expert system*” or “knowledge base*” or “decision tree*”) ) | 61,483 |
| S94 | TI ( (((activity or fitness) N3 tracker*) or acceleromet* or pedomet* or actigraph* or actimetr* or (wearable N3 (“electronic device*” or sensor*)) or ((step* or walk*) N3 (count* or meter* or daily)) or ((physical or physiology* or perform* or fit* or train* or active* or endur* or exercise) N3 (track* or monitor* or measur* or device* or app*))) ) OR AB ( (((activity or fitness) N3 tracker*) or acceleromet* or pedomet* or actigraph* or actimetr* or (wearable N3 (“electronic device*” or sensor*)) or ((step* or walk*) N3 (count* or meter* or daily)) or ((physical or physiology* or perform* or fit* or train* or active* or endur* or exercise) N3 (track* or monitor* or measur* or device* or app*))) ) | 119,522 |
| S95 | TI ( (computer* or laptop* or internet* or online or web-based or mobile* or app or apps or application* or telephone* or phone* or smartphone* or cellphone* or text messag* or SMS or “personal digital assistant*” or PDA* or “smart watch*” or “smart glasses” or Bluetooth or videoconferenc* or video-conferenc* or “web conference*” or webconference* or webcast* or “electronic mail*” or e-mail* or email* or “video game*” or exergame* or software* or hybrid or interactive or asynchron* or synchron* or ((cell* or smart* or mobile or android or internet or web or tablet*) N3 device*)) ) OR AB ( (computer* or laptop* or internet* or online or web-based or mobile* or app or apps or application* or telephone* or phone* or smartphone* or cellphone* or text messag* or SMS or “personal digital assistant*” or PDA* or “smart watch*” or “smart glasses” or Bluetooth or videoconferenc* or video-conferenc* or “web conference*” or webconference* or webcast* or “electronic mail*” or e-mail* or email* or “video game*” or exergame* or software* or hybrid or interactive or asynchron* or synchron* or ((cell* or smart* or mobile or android or internet or web or tablet*) N3 device*)) ) | 504,454 |
| S96 | TI ( (((information or wireless or remote* or biomedical or medical or health or digital* or communication) N3 technolog*) or (digital* N3 platform*) or ((artificial or ambient) N3 intelligence) or AI or ((augmented or virtual) N3 realit*)) ) OR AB ( (((information or wireless or remote* or biomedical or medical or health or digital* or communication) N3 technolog*) or (digital* N3 platform*) or ((artificial or ambient) N3 intelligence) or AI or ((augmented or virtual) N3 realit*)) ) | 49,637 |
| S97 | TI ( (((“user-centered” or “computer-aided”) N3 design*) or telecommunication* or ((information or intelligent) N3 system*) or “information processing” or (“decision support” N3 (system* or tech*)) or ((wireless or media*) N3 communication*) or ((health or nursing or medical) N3 informatic*)) ) OR AB ( (((“user-centered” or “computer-aided”) N3 design*) or telecommunication* or ((information or intelligent) N3 system*) or “information processing” or (“decision support” N3 (system* or tech*)) or ((wireless or media*) N3 communication*) or ((health or nursing or medical) N3 informatic*)) ) | 38,956 |
| S98 | S28 OR S29 OR S30 OR S31 OR S32 OR S33 OR S34 OR S35 OR S36 OR S37 OR S38 OR S39 OR S40 OR S41 OR S42 OR S43 OR S44 OR S45 OR S46 OR S47 OR S48 OR S49 OR S50 OR S51 OR S52 OR S53 OR S54 OR S55 OR S56 OR S57 OR S58 OR S59 OR S60 OR S61 OR S62 OR S63 OR S64 OR S65 OR S66 OR S67 OR S68 OR S69 OR S70 OR S71 OR S72 OR S73 OR S74 OR S75 OR S76 OR S77 OR S78 OR S79 OR S80 OR S81 OR S82 OR S83 OR S84 OR S85 OR S86 OR S87 OR S88 OR S89 OR S90 OR S91 OR S92 OR S93 OR S94 OR S95 OR S96 OR S97 | 848,478 |
| S99 | (MH "Home Rehabilitation") | 1,490 |
| S100 | (MH "Home Environment") | 11,913 |
| S101 | (MH "Home Care Equipment and Supplies") | 479 |
| S102 | (MH "Home Health Care") | 24,885 |
| S103 | (MH "Home Health Agencies") | 5,251 |
| S104 | (MH "Home Physical Therapy") | 542 |
| S105 | (MH "Home Occupational Therapy") | 371 |
| S106 | (MH "Home Nutritional Support") | 1,184 |
| S107 | (MH "Home Apnea Monitoring") | 85 |
| S108 | (MH "Home Nursing") | 3,735 |
| S109 | (MH "Home Nursing, Professional") | 7,473 |
| S110 | (MH "Home Health Aides") | 1,437 |
| S111 | (MH "Housing") | 9,453 |
| S112 | (MH "Housing for the Elderly") | 0 |
| S113 | (MH "Residence Characteristics") | 14,410 |
| S114 | (MH "Home Ownership") | 36 |
| S115 | (MH "Home Safety") | 1,327 |
| S116 | (MH "Accidents, Home") | 1,158 |
| S117 | (MH "Home Visits") | 6,620 |
| S118 | (MH "Home Maintenance") | 1,823 |
| S119 | (MH "Home Modification") | 159 |
| S120 | (MH "Home Health Care Information Systems") | 125 |
| S121 | (MH "Residential Care") | 7,021 |
| S122 | (MH "Residential Facilities") | 5,123 |
| S123 | (MH "Community Living") | 18,220 |
| S124 | (MH "Assisted Living") | 3,297 |
| S125 | (MH "Homemaker Services") | 455 |
| S126 | TI ( (home* or housing or house* or residential* or residence* or ((independent or communit* or assisted) N3 living) or dwell* or domestic* or domicile* or habitati* or abode*) ) OR AB ( (home* or housing or house* or residential* or residence* or ((independent or communit* or assisted) N3 living) or dwell* or domestic* or domicile* or habitati* or abode*) ) | 332,192 |
| S127 | S99 OR S100 OR S101 OR S102 OR S103 OR S104 OR S105 OR S106 OR S107 OR S108 OR S109 OR S110 OR S111 OR S112 OR S113 OR S114 OR S115 OR S116 OR S117 OR S118 OR S119 OR S120 OR S121 OR S122 OR S123 OR S124 OR S125 OR S126 | 376,419 |
| S128 | (MH "Randomized Controlled Trials") | 127,989 |
| S129 | (MH "Double-Blind Studies") | 52,809 |
| S130 | (MH "Single-Blind Studies") | 15,577 |
| S131 | (MH "Random Assignment") | 73,548 |
| S132 | (MH "Pretest-Posttest Design") | 49,199 |
| S133 | (MH "Cluster Sample") | 4,969 |
| S134 | TI (randomised OR randomized) | 127,215 |
| S135 | AB (random*) | 372,439 |
| S136 | TI (trial) | 164,044 |
| S137 | (MH "Sample Size") AND AB (assigned OR allocated OR control) | 4,337 |
| S138 | (MH "Placebos") | 13,350 |
| S139 | PT (randomized controlled trial) | 141,306 |
| S140 | AB (control W5 group) | 133,316 |
| S141 | (MH "Crossover Design") | 21,277 |
| S142 | (MH "Comparative Studies") | 436,296 |
| S143 | AB (cluster W3 RCT) | 452 |
| S144 | S128 OR S129 OR S130 OR S131 OR S132 OR S133 OR S134 OR S135 OR S136 OR S137 OR S138 OR S139 OR S140 OR S141 OR S142 OR S143 | 953,536 |
| S145 | S27 AND S98 AND S127 AND S144 | 604 |

**Database:** Google Scholar

**Date:**  19.04.2023

**Hits:** 300

stroke|poststroke|"brain infarction"|"transient ischemic attack"|hemiplegia home|domestic|dwell|community RCT|randomized|randomised|random telerehabilitation|telestroke|software|digital|online|computer|internet|"web-based"|telehealth|mhealth|ehealth

#

# **SUPPLEMENTAL FILE 3: Data extraction form and risk of bias tool**

General information

| Title or ID: |  |
| --- | --- |
| Primary author: |  |
| Report ID: |  |
| Year: |  |
| Country: |  |
| Study author contact details: |  |

| Study Characteristics | Eligibility criteria  *(Insert inclusion criteria for each characteristic as defined in the Protocol)* | | Eligibility criteria met? | | | Location in text or source *(pg & /fig/table/other)* |
| --- | --- | --- | --- | --- | --- | --- |
|  |  | | **Yes** | **No** | **Unclear** |  |
| Type of study | Randomised controlled trial with control group | |  |  |  |  |
| Participants | Adult stroke survivors (age ≥ 18) living at home (sub-acute and chronic stroke) | |  |  |  |  |
| Types of intervention | Health information or intervention combined with synchronous communication technology (e.g. chat, video-consultation) used on the web or application on the phone  It can be combined with wearable assessment/telemonitoring (e.g. VR, gait sensors).  If mixed with other interventions (telemonitoring), at least 50% of the intervention must be provided via telerehabilitation | |  |  |  |  |
| Types of intervention | Interventions with activity/mobility training **:** | |  |  |  |  |
|  | and/or secondary prevention: | |  |  |  |  |
| Types of outcome measures | Functional or activity outcomes (primary for this review): | |  |  |  |  |
|  | Stroke risk factor outcomes: | |  |  |  |  |
|  | Self-reported outcomes: | |  |  |  |  |
| Types of control group: | Usual care, in-patient rehabilitation,  placebo, or no-treatment, waiting list  (control group with no digital follow-up) | | | | | |
| Reason for exclusion/Notes: | | | | | | |
| INCLUDE | | **EXCLUDE** | | | | |

**DO NOT PROCEED IF THE STUDY IS EXCLUDED FROM REVIEW**

Design

**RCT/pilot-RCT or feasibility design:**

☐**Parallel group** (each participant is randomly assigned to a group, and all the participants in the group receive (or do not receive) an intervention.)

**☐Crossover** (over time, each participant receives (or does not receive) an intervention in a random sequence.)

☐**Cluster** (pre-existing groups of participants (e.g., schools) are randomly selected to receive (or not receive) an intervention.)

☐**Factorial** (In a factorial trial, two (or more) intervention comparisons are carried out simultaneously and each participant is randomly assigned to a group that receives a particular combination of interventions or non-interventions)

☐**Other:……………………………………………………………………………………………………………………………….**

Type of control group:

☐Waiting list ☐Care as usual/in-or out-patient rehabilitation ☐Active control ☐ Placebo ☐ No treatment

|  | **Descriptions as stated in report/paper** |
| --- | --- |
| **Aim of study** |  |

Study population:

|  | Intervention | Control group |
| --- | --- | --- |
| Total population |  |  |
| Setting |  |  |
| Diagnostic criteria *(e.g., how were they diagnosed and level of illness (motor deficits, NIHSS etc.)* |  |  |
| Age |  |  |
| Sex/gender |  |  |
| Baseline imbalances |  |  |

Intervention/Comparison:

|  | Intervention | Control group *(some details would not be applicable for control group)* |
| --- | --- | --- |
| Intervention description:   - description of functionalities - technological description: bi-or unidirectional - asynchronized or synchronised |  |  |
| Intervention providers (who are delivering the intervention?) |  |  |
| Duration  Dose  Intensity (number of sessions, hours) |  |  |
| Intervention compliance/adherence: |  |  |

What type of problem does the eHealth intervention target (please add)?

☐ Activity of daily living ☐Alcohol use ☐ Cognition

☐ Nutrition ☐Smoking ☐ Lifestyle changes

☐ Physical activity ☐ Obesity/weight ☐ Pain

☐ Sedentary behaviour ☐Depression ☐Anxiety

☐ Cardiovascular risk ☐Stress ☐ Mobility

How was the eHealth intervention offered?

☐Purely digital ☐Blended (combination of online components and therapeutic (human) guidance)

Outcomes:

|  | Intervention and control |
| --- | --- |
| Outcome category and scale/name of measurement:  *(Primary and secondary*) |  |
| Total score and interpretation of measurement/scale:  Scales: upper and lower limits *(indicate whether high or low score is good)* |  |
| Time points: | ☐Baseline  ☐Post-intervention (up to 30 days after intervention):____  ☐Short term (2-3 months follow-up):______  ☐Intermediate (6 months follow-up):_______  ☐Long- term (12 months follow-up):_____  ☐Other |
| Reliability of the instruments:  (Cronbach alfa value if relevant. *Cronbach’s Alpha is usually used for testing the reliability of measurements given by questionnaires (ordinal or nominal data*)  Interclass correlation coefficient (ICC) |  |
| Is outcome/tool validated? | Yes No Unclear |
| Blinded tester? | Yes No Unclear |
| Person measuring/ reporting: |  |
| Validity of the instruments:  [Construct validity](https://www.scribbr.com/methodology/types-of-validity/#construct-validity): *Does the test measure the concept that it’s intended to measure?*  [Content validity](https://www.scribbr.com/methodology/types-of-validity/#content-validity): *Is the test fully representative of what it aims to measure?*  [Face validity](https://www.scribbr.com/methodology/types-of-validity/#face-validity): *Does the content of the test appear to be suitable to its aims?*  [Criterion validity:](https://www.scribbr.com/methodology/types-of-validity/#criterion-validity) *Do the results accurately measure the concrete outcome they are designed to measure?* |  |

Was the eHealth intervention effective?

☐Yes

Specify which outcome:

☐No differences

Specify which outcome:

☐Adverse effect

Results:

|  | Intervention | Control |
| --- | --- | --- |
| Sample size |  |  |
| Missing data/dropouts |  |  |
| Estimate of effect/power (p-value, CI-interval) |  |  |
| Methods of missing data imputation |  |  |

Dichotomous outcome

*Copy and paste the appropriate table for each outcome, including additional tables for each time point and subgroup as required.*

| Outcome | Time | Intervention group | | Control arm | | Notes | Location in text |
| --- | --- | --- | --- | --- | --- | --- | --- |
| *Note whether:*  *post-intervention OR*  *change from baseline*  *And whether*  *Adjusted OR*  *Unadjusted* |  | Observed events (No. Of events) | Sample size  (No. Of participants) | Observed events | Sample size |  |  |
|  |  |  | |  | |  |  |
|  |  |  | |  | |  |  |
| **Baseline data** |  |  | |  | |  |  |
| **Statistical methods used and appropriateness of these methods** |  |  | |  | |  |  |

Continuous data

| **Outcome** | **Time** | **Intervention group** | | | **Control arm** | | | **Notes** | **Location in text** |
| --- | --- | --- | --- | --- | --- | --- | --- | --- | --- |
| *Note whether:*  *post-intervention OR*  *change from baseline*  *And whether*  *Adjusted OR*  *Unadjusted* |  | Sample size (No. of participant) | Mean change (incl. range) | Standard deviation | Sample size | Mean change (incl. range) | Standard deviation |  |  |
|  |  |  | | |  | | |  |  |
|  |  |  | | |  | | |  |  |
|  |  |  | | |  | | |  |  |
| **Baseline data** |  |  | | |  | | |  |  |
|  |  |  | | |  | | |  |  |
| Statistical methods used and appropriateness of these methods |  |  | | |  | | |  |  |

## Risk of Bias assessment

*See* [*Chapter 8*](http://www.mrc-bsu.cam.ac.uk/cochrane/handbook/index.htm#chapter_8/8_assessing_risk_of_bias_in_included_studies.htm) *of the Cochrane Handbook. Additional domains may be required for non-randomised studies.*

| **Domain** | | **Risk of bias**  *Low/ High/Unclear* | **Support for judgement** | **Location in text**  *(pg & ¶/fig/table)* |
| --- | --- | --- | --- | --- |
| 1. Random sequence generation   *(selection bias)* | |  |  |  |
| 1. Allocation concealment   *(selection bias)* | |  |  |  |
| 1. Blinding of participants and personnel   *(performance bias)* | |  | **Outcome group: All/** |  |
| *(if required)* | |  | **Outcome group:** |  |
| 1. Blinding of outcome assessment   *(detection bias)* | |  | **Outcome group: All/** |  |
| *(if required)* | |  | **Outcome group:** |  |
| 1. Incomplete outcome data   *(attrition bias)* | |  |  |  |
| 1. Selective outcome reporting?   *(reporting bias)* | |  |  |  |
| 1. Other bias | |  |  |  |
| 1. Notes: |  | | | |

# **SUPPLEMENTARY FILE 4: Included and excluded studies**

**Study ID/Title Include/Exclude Main reasons Remarks**

| Adams 2023 Telehealth-Guided Virtual Reality for Recovery of Upper Extremity Function Following Stroke  **Include**  Virtual reality home exercise program (included a wearable glove) and asynchronous and synchronous supervision,  virtual reality home exercise program (included a wearable glove) and asynchronous and synchronous supervision | Allegue 2022 "Rehabilitation of Upper Extremity by Telerehabilitation Combined With Exergames in Survivors of Chronic Stroke: Preliminary Findings From a Feasibility Clinical Trial"  **Include**  Jintronix exergames for upper extremity rehabilitation and Reacts app with videoconference | Anwar 2022 Virtual Reality Training Using Nintendo Wii Games for Patients With Stroke: Randomized Controlled Trial  **Exclude**  No digital communication. Hospital-setting | Aphiphaksakul 2022 Home-based exercise using balance disc and smartphone inclinometer application improves balance and activity of daily living in individuals with stroke: A randomized controlled trial  **Exclude**  No digital communication | Aprile 2022 Robotic telerehabilitation: a feasibility study in patients with stroke  **Exclude**  No control group |
| --- | --- | --- | --- | --- |
| Baluz 2022 Motor Rehabilitation of Upper Limbs Using a Gesture-Based Serious Game: Evaluation of Usability and User Experience  **Exclude**  No mobility outcomes | Berg 2016 Early supported discharge by caregiver-mediated exercises and e-Health support after stroke  **Exclude**  24h-3 months post stroke Others. | Bhatnagar 2020 Comparing Home Upper Extremity Activity with Clinical Evaluations of Arm Function in Chronic Stroke  **Exclude**  a Cross-sectional study analysing baseline data from a larger RCT (cannot find the full study) + arm accelerometers | Bizovicar 2018 Tele-rehabilitation service at home for patients after stroke  **Exclude**  Unable to contact author/No response/Not published/unpublished results Others. | Blanton 2022 Evaluation of a CarePartner-Integrated Telehealth Rehabilitation Program for Persons With Stroke (CARE-CITE)  **Exclude**  Results published in clinicaltrial.gov |
| Burdea 2020 Feasibility of integrative games and novel therapeutic game controller for telerehabilitation of individuals chronic post-stroke living in the community  **Exclude**  No control group and not RCT | Burridge 2014 Development and Pilot Evaluation of a Web-supported Programme of Constraint Induced Therapy Following Stroke (LifeCIT)  **Exclude**  Unable to contact author/No response/Not published/unpublished results | Cameron 2022 A mixed-methods feasibility study of a new digital health support package for people after stroke: the Recovery-focused Community support to Avoid readmissions and improve Participation after Stroke (ReCAPS) intervention  **Exclude**  No mobility outcomes. Not right intervention. Only electronic support messages | Chang 2021 Effects of Mhealth App on the Improvement of Upper Limb Function for Patients With Chronic Stroke  **Exclude**  Unable to contact author/No response/Not published/unpublished results |  |
| Chaporrov 2018 Home-based physical activity incentive and education program in subacute phase of stroke recovery (Ticaa'dom): study protocol for a randomized controlled trial  **Exclude**  Unable to contact author/No response/Not published/unpublished results | Chau 2022 Effects of a social participation-focused virtual reality intervention for community-dwelling stroke survivors with physical disabilities: a randomised controlled trial protocol  **Exclude**  Protocol. Authors contacted. Study is still progress and the data may only be available next year | Chen 2021 Effectiveness of a home-based exercise program among patients with lower limb spasticity post-stroke: A randomized controlled trial  **Exclude**  Not digital. Nurse–guided home-based rehabilitation exercise program (HREPro) | Chen 2022 Internet+Continuing Nursing (ICN) programme promotes motor function rehabilitation of patients with ischemic stroke  **Include**  WeChat + Lantern Follow-up Management System – no active communication in telerehabilitation | Chen, Chien-Hsi 2022 Rehago - A Home-Based Training App Using Virtual Reality to Improve Functional Performance of Stroke Patients with Mirror Therapy and Gamification Concept: A Pilot Study  **Exclude**  No control group |
| Chen, J 2020 Effects of home-based telerehabilitation in patients with stroke: A randomized controlled trial  **Exclude**  acute patients within 1-3 weeks after stroke | Christie 2022 Remote constraint induced therapy of the upper extremity (ReCITE): A feasibility study protocol  **Exclude**  Protocol. Authors contacted. | Chumbler 2012 Effects of Telerehabilitation on Physical Function and Disability for Stroke Patients  **Exclude**  Experienced stroke within 24 months. | Conroy 2020 "Novel use of existing technology: A preliminary study of patient portal use for telerehabilitation"  **Include**  "A Web based patient portal (PP) enabled e-visits by supporting patient–provider messaging. Asynchronous communication." |  |
|  |  | Cramer 2019 "Efficacy of Home-Based Telerehabilitation vs In-Clinic Therapy for Adults After Stroke A Randomized Clinical Trial"  **Include**  System software supported videoconferencing and organized the 70 minutes of therapy, which consisted of exercises, functional games, and stroke education |  |  |
| De La Torre Costa 2021 A combination of computer-based and wearable systems to remotely promote and monitor recovery and arm use post-stroke: Preliminary results of a randomised controlled trial  **Exclude**  Unable to contact author/No response/Not published/unpublished results | Deng 2012 Complex Versus Simple Ankle Movement Training in Stroke Using Telerehabilitation: A Randomized Controlled Trial  **Exclude**  Lack of control group that fulfils the criteria. Both intervention and control utilised the computerised programme + teleconferencing | Duncan 2020 Randomized Pragmatic Trial of Stroke Transitional Care The COMPASS Study  **Exclude**  Not digital. Compass -transitional care. | English 2021 Secondary prevention of stroke – a study protocol of telehealth-delivered physical activity and diet pilot RCT  **Exclude**  Control group with digital follow-up. The control group will receive information (links to online resources) about physical activity and diet in addition to 2 telehealth sessions. | Escalante-Gonzalbo 2022 Changes in Motor Function and Brain Connectivity on Subacute Stroke Patients, Associated With Videogame Therapy  **Exclude**  Author contacted/no published results |
| Feng 2021 Application effect of the hospital-community integrated service model in home rehabilitation of stroke in disabled elderly: a randomised trial  **Exclude**  No significant digital intervention - chat group only. Others. | Fluet 2019 Utilizing Gaming Mechanics to Optimize Telerehabilitation Adherence in Persons With Stroke  **Exclude**  Author contacted/no published results. no usual care control group. Comparing two approaches to technology supported rehabilitation | Gaboury Effects of Telerehabilitation on Patient Adherence to a Rehabilitation Plan  **Exclude**  Author contacted/no published results | Gauthier 2021 "Video game rehabilitation for outpatient stroke (VIGoROUS): A multi-site randomized controlled trial of in-home, self-managed, upper-extremity therapy"  **Include**  Telegaming and video consults |  |
| Gensitz 2022 More than a training app - Does personalized coaching via Messenger promote stroke sufferers' compliance with their app-based training?  **Exclude**  Unable to contact author/No response/Not published/unpublished results | Grau-Pellicer 2019 Impact of mHealth technology on adherence to healthy PA after stroke: a randomized study  **Exclude**  Digital platform combined with rehabilitation at hospital | Guillaumier 2022 Evaluation of an online intervention for improving stroke survivors' health-related quality of life: A randomised controlled trial  **Exclude**  No HCP involved in intervention. Others. Control group had digitalised generic information given - considered non-active. No HCP involved in intervention - remote programme. | Haesebaert 2021 Development and Evaluation of a Patient-centred Transition Program for Stroke Patients, Combining Case Management and Access to an Internet Information Platform (NAVISTROKE)  **Exclude**  Unable to contact author/No response/Not published/unpublished results | Hernandez 2022 "Virtual Reality–Based Rehabilitation as a Feasible and Engaging Tool for the Management of Chronic Poststroke Upper-Extremity Function Recovery: Randomized Controlled Trial"  **Include**  "Jintronix system as a remotely supervised home-based program for upper extremity rehabilitation" |
| Honeycutt 2021 Addressing Socioeconomic Disparities in Post-stroke Upper-extremity Disability Through the Development of an Accessible, New Tool  **Exclude**  Author contacted/no published results | Hu 2022 Digital Graphic Follow-up Tool (Rehabkompassen) for Identifying Rehabilitation Needs Among People After Stroke: Randomized Clinical Feasibility Study  **Exclude**  No mobility outcomes. Outpatient setting | Jarbandhan 2022 Feasibility of a home-based physiotherapy intervention to promote post-stroke mobility: A randomized controlled pilot study  **Exclude**  Not digital program, only digital communication | Juanjuan 2021 Effect of sports training guidance based on Internet platform in home rehabilitation of stroke hemiplegic patients  **Exclude**  Chinese language | Kilbride 2022 Rehabilitation using virtual gaming for Hospital and home-based training for the Upper limb post stroke (Rhombus II): protocol of a feasibility randomised controlled trial  **Exclude**  No digital communication |
| Kizoni 2022 Evaluation of a Tele-Health System for Upper Extremity Stroke Rehabilitation  **Include**  "quasi home based Telemotion rehabilitation system Gertner system" |  |  |  |  |
| Krpic 2013 "Telerehabilitation: remote multimedia-supported assistance  and mobile monitoring of balance training outcomes can facilitate the clinical staff’s effort"  **Include** | Kwon 2022 Effects of balance training using a virtual reality program in hemiplegic patients  **Exclude**  No digital communication | Lansberg 2022 Home-based virtual reality therapy for hand recovery after stroke  **Exclude**  No control group | Lee 2018 Therapeutic potential of the home-based exercise program with the augmented reality system on balance in stroke patients: a preliminary report  **Exclude**  Unable to contact author/No response/Not published/unpublished results | Lee 2021 Effects of the home-based exercise program with an augmented reality system on balance in patients with stroke: a randomized controlled trial  **Exclude**  No digital communication |
| Li 2020 "A Mobile Health App for the Collection of Functional Outcomes After Inpatient Stroke Rehabilitation: Pilot Randomized Controlled Trial"  **Exclude**  Not the right intervention and aim. The aim was to test validity and reliability of functional assessment between these 2 modes of administration " | Li 2021 Long-term effectiveness and adoption of a cell phone augmented reality system on patients with stroke: randomized controlled trial  **Exclude**  "Combined acute and subacute patients. Chronicity 7-180 days.  Game-based cell phone augmented reality rehabilitation system (CARS) for improving both motor function and cognition function of stroke survivors. No digital communication." Others. | Lin 2011 Development of Smart Holistic Telerehabilitation System: An Application of Stroke Patients- 3 Years Study  **Exclude**  Unable to contact author/No response/Not published/unpublished results | Lindley 2016 Attend (family led rehabilitation after stroke in India) trial: potential for better stroke rehabilitation access in India  **Exclude**  No RCT/Unable to contact author | Llorens 2015 Effectiveness, usability, and cost-benefit of a virtual reality-based telerehabilitation program for balance recovery after stroke: A randomized controlled trial  **Exclude**  Focus was home-based vs in-clinic. Both received same digitalized VR intervention. Physical sessions for both as well. Unclear whether it was digital communication with HCP. Others. |
| Maier 2020 Adaptive conjunctive cognitive training (ACCT) in virtual reality for chronic stroke patients: A randomized controlled pilot trial  **Exclude**  "Focus was cognitively impaired pts (MoCA<26). The intervention took place in the same hospital and not at home. No digital communication." Others. | McCormick 2022 Technology-Dependent Rehabilitation Involving Action Observation and Movement Imagery for Adults with Stroke: Can It Work? Feasibility of Self-Led Therapy for Upper Limb Rehabilitation after Stroke  **Exclude**  No control group | Merchan-Baeza 2018 Clinical effect size of an educational intervention in the home and compliance with mobile phone-based reminders for people who suffer from stroke: protocol of a randomized controlled trial  **Exclude**  Unable to contact author/No response/Not published/unpublished results | Montiel 2019 "Farmalarm Application for Mobile Devices Improves Risk Factor Control After Stroke"  **Exclude**  Not RCT/author contacted/control group with digital intervention |  |
| Mulder 2022 Can telerehabilitation services combined with caregiver-mediated exercises improve early supported discharge services poststroke? A study protocol for a multicentre, observer-blinded, randomized controlled trial  **Exclude**  Unable to contact author/No response/Not published/unpublished results | Mura 2022 Bringing rehabilitation home with an e-health platform to treat stroke patients: study protocol of a randomized clinical trial (RGS@home)  **Exclude**  Protocol. Authors contacted. | Ormen 2021 The Effect of Individualized Home-Based Telerehabilitation Intervention on Physical Parameters and Activities of Daily Living in Individuals With Stroke  **Exclude**  Unable to contact author/No response/Not published/unpublished results | Ortiz-Fern 2019 Efficacy and Usability of eHealth Technologies in Stroke Survivors for Prevention of a New Stroke and Improvement of Self-Management: Phase III Randomized Control Trial  **Exclude**  Unable to contact author/No response/Not published/unpublished results | Paul 2018 Increasing physical activity in stroke survivors using STARFISH, an interactive smartphone application: Protocol for a randomised controlled trial  **Exclude**  Unable to contact author/No response/Not published/unpublished results |
| Piron 2009 "EXERCISES FOR PARETIC UPPER LIMB AFTER STROKE: A COMBINED VIRTUAL-REALITY AND TELEMEDICINE APPROACH"  **Include** | Poulsen 2022 Early web-based tele-rehabilitation in stroke patients: A randomised controlled pilot study  **Exclude**  Unable to contact author/No response/Not published/unpublished results | Pruitt 2023 Usage of RePlay as a Take-Home System to Support High-Repetition Motor Rehabilitation After Neurological Injury  **Exclude**  Combination of neurologic diseases. Not randomized. Not digital communication. Wrong outcome. | Rosbergen 2020 iN home Telerehabilitation to ENhance phySIcal activiTY after Stroke (iNTENSITY - Stroke)  **Exclude**  Unable to contact author/No response/Not published/unpublished results | Salguiro 2022 Telerehabilitation for balance rehabilitation in the subacute stage of stroke: A pilot-controlled trial  **Include**  Farmalarm App as a telerehabilitation tool to guide home-based core stability exercises. Neurological physiotherapist available for video calls using the App. |
| Saposnik 2022 iPad technology for home rehabilitation after stroke (iHOME): a proof-of-concept randomized trial  **Exclude**  Unable to contact author/No response/Not published/unpublished results | Saygili 2021 The Effectiveness of Modified-Constraint Induced Movement Therapy Based Telerehabilitation in Stroke Patients  **Exclude**  Unable to contact author/No response/Not published/unpublished results | Saywell 2016 Augmented community telerehabilitation intervention to improve outcomes for people with stroke AKTIV-a randomised controlled trial  **Exclude**  Thesis with RCT, ACTIV-intervention combined of landline telephone, messaging and home visits. Lack motor primary outcome. | Seregni 2022 Virtual coaching system for continuity of care and rehabilitation in patients with stroke  **Exclude**  No control group | Shaw 2021 RehabTouch: A Mixed-reality Gym for Rehabilitating the Hands, Arms, Trunk, and Legs After Stroke  **Exclude**  Unclear if population and intervention is eligible |
| Sheeny 2019 "Home-based virtual reality training after discharge from hospital-based stroke rehabilitation: a parallel randomized feasibility trial"  **Exclude**  "No communication. Home-based virtual reality training. Control group participants use an iPad with apps selected to rehabilitate cognition, hand fine motor skills and visual tracking/scanning." | Simpson 2022 Virtual Arm Boot Camp (V-ABC). Study protocol for a mixed-methods study to increase upper limb recovery after stroke with an intensive program coupled with a grasp count device  **Exclude**  Protocol. Authors contacted. Study still in progress. Expect this to be done by end of 2023 | Swanson 2023 Optimized Home Rehabilitation Technology Reduces Upper Extremity Impairment Compared to a Conventional Home Exercise Program  **Exclude**  No digital communication and supervision | Tenforde 2020 Evidence-Based Physiatry: Efficacy of Home-Based Telerehabilitation Versus In-Clinic Therapy for Adults After Stroke  **Exclude**  Not RCT Evidence-Based Physiatry: Efficacy of Home-Based Telerehabilitation Versus In-Clinic Therapy for Adults After Stroke (oslomet.no) | Termoz 2022 Co-design and evaluation of a patient-centred transition programme for stroke patients, combining case management and access to an internet information platform: study protocol for a randomized controlled trial – NAVISTROKE  **Exclude**  Unable to contact author/No response/Not published/unpublished results |
| Thompson-Butel 2022 A telehealth transfer package to improve upper limb rehabilitation post-stroke - The protocol  **Exclude**  Author contacted/no published results | Thurston 2023 Mobile health to promote physical activity in people post stroke or transient ischemic attack – study protocol for a feasibility randomised controlled trial  **Exclude**  Protocol | unknown Effectiveness of Tele-rehabilitation Intervention to Improve Performance, Participation and Quality of Life for People Post Stroke  **Exclude**  Unable to contact author/No contact information | Uswatte 2021 Tele-rehabilitation of upper-extremity hemiparesis after stroke: Proof-of-concept randomized controlled trial of in-home Constraint-Induced Movement therapy  **Include**  TeleCIMT with remote supervision. Implied no cognitive impairment. | van den Berg 2016 "Early Supported Discharge by Caregiver-Mediated Exercises and e-Health Support After Stroke A Proof-of-Concept Trial"  **Exclude**  acute patients |
| van der Ven 2017 "The influence of computer-based cognitive flexibility training on subjective cognitive wellbeing after stroke: A multi-center randomized controlled trial"  **Exclude**  Study with only cognitive or speech/language/communication therapy intervention, lack of communication technology + lack of functional intervention and outcomes | Vismara 2022 Tele-Monitoring and Tele-Rehabilitation of the Hand in Hemiplegic Patients  **Exclude**  Conference paper. No control group | Vloothuis 2019 "Caregiver-mediated exercises with e-health support for early supported discharge after stroke (CARE4STROKE): A randomized controlled trial"  **Exclude**  acute patients + In-patient rehabilitation setting | Wong 2020 Feasibility of Mobile Self-Management Intervention for Mild Stroke  **Exclude**  Unable to contact author/No response/Not published/unpublished results | Wu 2020 "Collaborative Care Model Based Telerehabilitation Exercise Training Program for Acute Stroke Patients in China: A Randomized Controlled Trial"  **Exclude**  Need to contact authors to check for eligibility of participants. The study starts after discharge from hospital, but the exact time since stroke is not stated anywhere, other than it is stated acute stroke |
| Wu, Na 2019 A Smart and Multifaceted Mobile Health System for Delivering Evidence-Based Secondary Prevention of Stroke in Rural China: Design, Development, and Feasibility Study  **Exclude**  qualitative study of the design and the development of SINEMA system with doctors and end-users | Yang 2022 Augmented reality for stroke rehabilitation during Covid-19  **Exclude**  No true control group. Control group is also testing same digital solution in hospital setting | Yeh et al 2022 "Clinical efficacy of aerobic exercise combined with computer-based cognitive training in stroke: a multicentre randomized controlled trial"  **Exclude**  Exclude due to combination of digital cognitive training and not digital physical training. Assumingly hospital-setting | Yosef 2022 A performance-based teleintervention for adults in the chronic stage after acquired brain injury  **Exclude**  No mobility outcomes |  |
| Zedda 2020 DoMoMEA: A home-based telerehabilitation system for stroke patients  **Exclude**  Author contacted, Trial not started and no published results Others. | Zhang 2022 Coaching-Based Teleoccupational Guidance for Home-Based Stroke Survivors and Their Family Caregivers: Study Protocol for a Superior Randomized Controlled Trial  **Exclude**  No true control group. Control group also gets standard telerehabilitation | Zhonghe 2023 Application of remote multidisciplinary continuous nursing care in stroke patients with home rehabilitation  **Exclude**  Protocol | Zhang 2023 Influence on Occupational Performance of Home-based Stroke Patients by Coaching-based Tele-occupational Guidance  **Exclude**  Protocol | Song 2023 Application of rehabilitation guidance on remote platform in patients with stroke  **Exclude**  Acute patients |
| Verschure 2023 ACCEPTABILITY AND USABILITY ANALYSIS OF A HOME-BASED UPPER LIMB TELEREHABILITATION SYSTEM BASED ON VIRTUAL REALITY FOR POST-STROKE PATIENTS: MIXED METHODS IN A RANDOMIZED CONTROLLED TRIAL  **Exclude**  Interesting conference paper. Mixed-method study, use TAM questionnaire. Assumingly wrong outcome due to feasibility trial | del Rocio 2023 Feasibility of the implementation of an intensive upper-limb rehabilitation system (NeuroVirt) intervention for stroke survivors  **Exclude**  protocol, ongoing project | Jiang 2023 Study on self-management of real-time and individualized support in stroke patients based on resilience  **Exclude**  protocol, wrong outcome | Kang 2023 Home-Based Virtual Reality Exergame Program after Stroke Rehabilitation for Patients with Stroke: A Study Protocol for a Multicenter, Randomized Controlled Trial  **Exclude**  protocol | Kashyap 2023 Therapeutic efficacy of telerehabilitation in stroke care Exclude Could not be retrieved |
| Kuo 2023 Internet of Things (IoT) Enables Robot-Assisted Therapy as a Home Program for Training Upper Limb Functions in Chronic Stroke: A Randomized Control Crossover Study  **Exclude**  Lack of digital supervision, cross-over groups, lack of program | Lee 2023 Telehealth Self-Management Support in Early Stroke Rehabilitation: A Feasibility Randomized Controlled Trial  **Exclude**  acute patients | Li 2023 Utilizing novel smart wearable assistive technology to facilitate sensorimotor training of stroke survivors: Towards home/community-based rehabilitation  **Exclude**  only monitoring intervention combined with supervision by app | HAESEBAERT 2023 Evaluation of the Feasibility of a Patient-centred Transition Program for Stroke Patients and Their Informal Caregivers, Combining Follow-up by a Case-manager and Access to an Internet Information Platform (P-NAVISTROKE)  **Exclude**  not digital, protocol |  |

# **SUPPLEMENTARY FILE 5: Categorization of studies**

#

# **SUPPLEMENTARY FILE 6: Risk of bias 1**


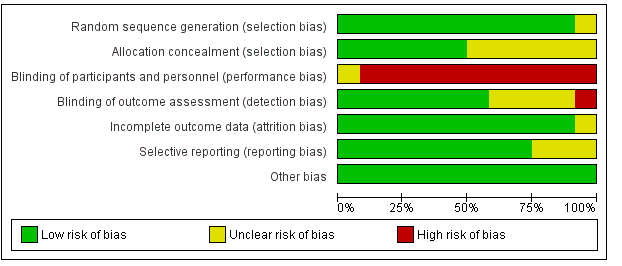


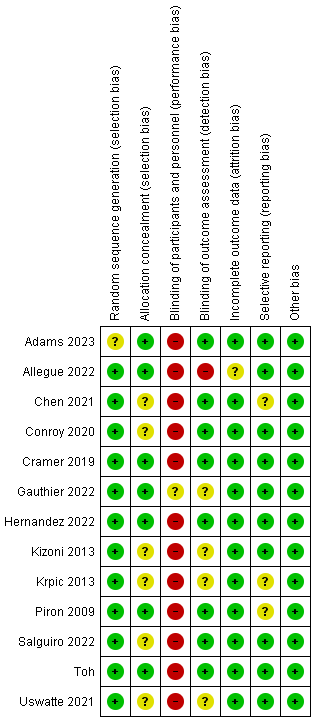


# **SUPPLEMENTARY FILE 7: Subgroup analysis motor ability of upper limb**


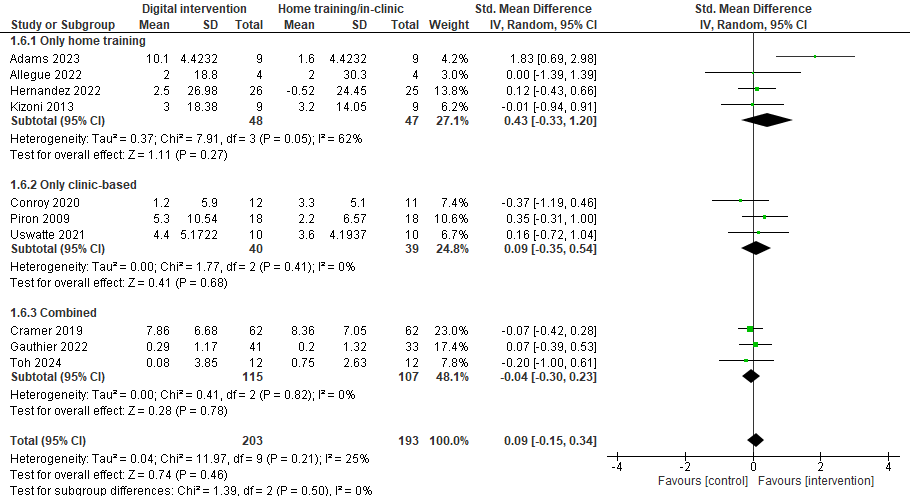


# **SUPPLEMENTARY FILE 8: Subgroup analysis stroke-related quality of life**


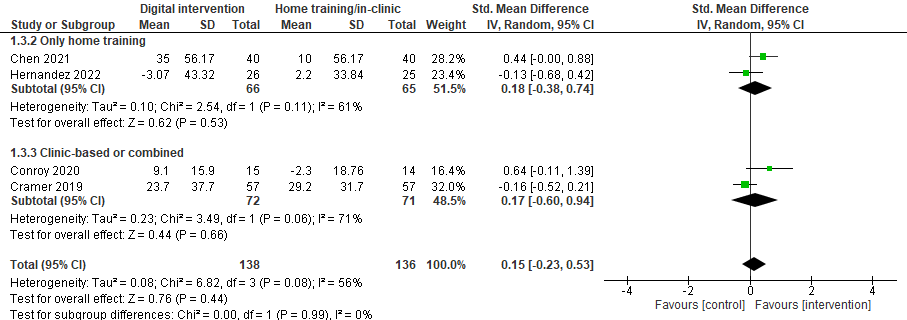


#

# **SUPPLEMENTARY FILE 9: Subgroup analysis self-reported arm function A. Comparator B. Instrument use C. Length of intervention time**

A)


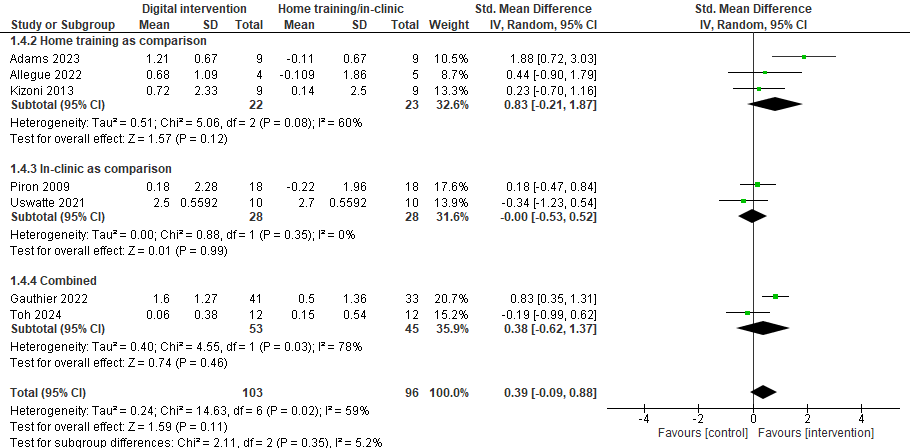


B)


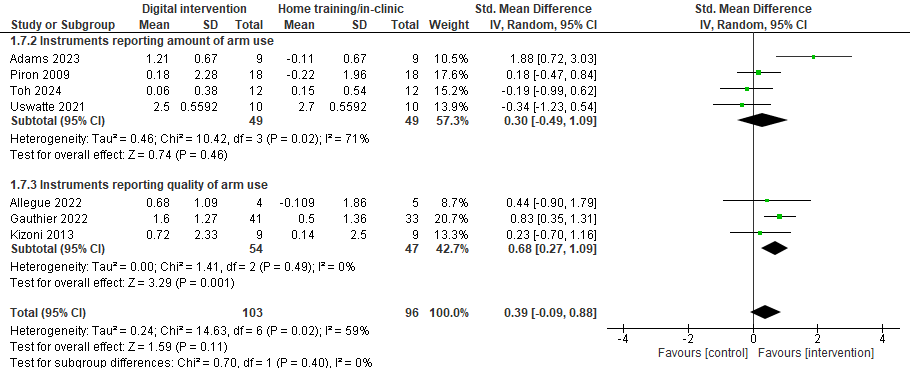


C)


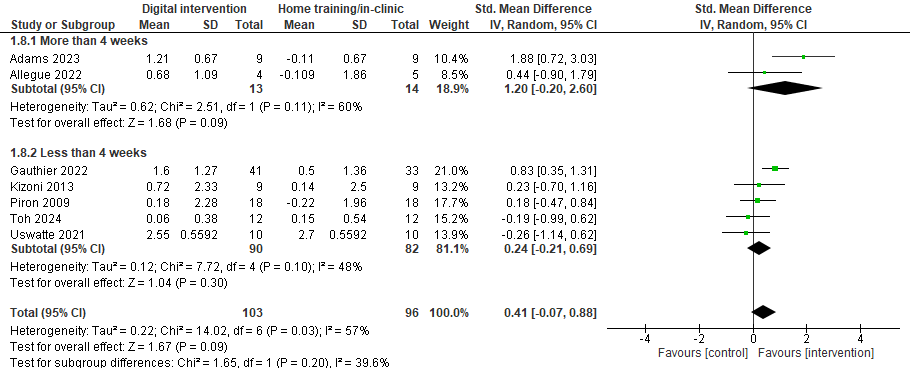

Supplement: sj-docx-1-dhj-10.1177_20552076241256861 - Supplemental material for Effectiveness of digital home rehabilitation and supervision for stroke survivors: A systematic review and meta-analysis [file sj-docx-1-dhj-10.1177_20552076241256861.docx]
